# Acai Berry (*Euterpe* sp.) Extracts Are Neuroprotective against L-Glutamate-Induced Toxicity by Limiting Mitochondrial Dysfunction and Cellular Redox Stress

**DOI:** 10.3390/life13041019

**Published:** 2023-04-15

**Authors:** Maryam N. ALNasser, Ayman M. AlSaadi, Alison Whitby, Dong-Hyun Kim, Ian R. Mellor, Wayne G. Carter

**Affiliations:** 1 Department of Biological Sciences, College of Science, King Faisal University, P.O. Box No. 400, Al-Ahsa 31982, Saudi Arabia; Maryam.al-nasser1@nottingham.ac.uk; 2 School of Life Sciences, Faculty of Medicine and Health Sciences, University of Nottingham, Nottingham NG7 2RD, UK; ian.mellor@nottingham.ac.uk; 3 School of Medicine, Royal Derby Hospital Centre, University of Nottingham, Derby DE22 3DT, UK; mzxaa24@exmail.nottingham.ac.uk; 4 Children’s Brain Tumour Research Centre, School of Medicine, Biodiscovery Institute, University of Nottingham, Nottingham NG7 2RD, UK; mszaw2@exmail.nottingham.ac.uk; 5 Centre for Analytical Bioscience, Advanced Materials and Healthcare Technologies Division, School of Pharmacy, University of Nottingham, Nottingham NG7 2RD, UK; dong-hyun.kim@nottingham.ac.uk

**Keywords:** acai berry, antioxidant, *Euterpe oleracea*, excitotoxicity, L-glutamate, neuroprotection, nutraceuticals

## Abstract

Aberrant accumulation of the neurotransmitter L-glutamate (L-Glu) has been implicated as a mechanism of neurodegeneration, and the release of L-Glu after stroke onset leads to a toxicity cascade that results in neuronal death. The acai berry (*Euterpe oleracea*) is a potential dietary nutraceutical. The aim of this research was to investigate the neuroprotective effects of acai berry aqueous and ethanolic extracts to reduce the neurotoxicity to neuronal cells triggered by L-Glu application. L-Glu and acai berry effects on cell viability were quantified using 3-(4,5-Dimethylthiazol-2-yl)-2,5-diphenyltetrazolium bromide (MTT) and lactate dehydrogenase (LDH) assays, and effects on cellular bioenergetics were assessed via quantitation of the levels of cellular ATP, mitochondrial membrane potential (MMP), and production of reactive oxygen species (ROS) in neuroblastoma cells. Cell viability was also evaluated in human cortical neuronal progenitor cell culture after L-Glu or/and acai berry application. In isolated cells, activated currents using patch-clamping were employed to determine whether L-Glu neurotoxicity was mediated by ionotropic L-Glu-receptors (iGluRs). L-Glu caused a significant reduction in cell viability, ATP, and MMP levels and increased ROS production. The co-application of both acai berry extracts with L-Glu provided neuroprotection against L-Glu with sustained cell viability, decreased LDH production, restored ATP and MMP levels, and reduced ROS levels. Whole-cell patch-clamp recordings showed that L-Glu toxicity is not mediated by the activation of iGluRs in neuroblastoma cells. Fractionation and analysis of acai berry extracts with liquid chromatography-mass spectrometry identified several phytochemical antioxidants that may have provided neuroprotective effects. In summary, the acai berry contains nutraceuticals with antioxidant activity that may be a beneficial dietary component to limit pathological deficits triggered by excessive L-Glu accumulations.

## 1. Introduction

L-glutamate (L-Glu) is an excitatory neurotransmitter involved in signaling and communication between neurons of the central nervous system (CNS) [1,2,3]. It is a non-essential amino acid and the most abundant free amino acid in the CNS [1,2,3]. Several critical roles for L-Glu in the development and function of the normal brain have been recognized; as well as it being a key regulator in the process of communication between neurons, it contributes to their plasticity, development, and energy supply [4,5]. Additionally, it contributes to learning and memory processes, as well as neural long-term potentiation [2].

Under normal conditions, L-Glu concentrations in plasma range from 50 to 100 µM, whereas in extracellular fluids it is in the much lower range of 0.5–2 µM [6]. The concentration of L-Glu within the excitatory synaptic cleft normally rises to the relatively high level of ≈1 mM after its release, but it only remains at this level for a few milliseconds and then returns to normal levels, due to high-affinity transporters expressed on neurons and glia [7,8].

Adverse health effects may arise if there is an aberrant increase in the concentration of L-Glu [4]. Mechanisms within neurons, astrocytes, and the blood–brain barrier abluminal membrane offer an effective means for the removal of excess L-Glu from the brain’s extracellular fluid [9]. The glutamate–glutamine cycle permits glial cells and presynaptic terminals to collectively ensure an adequate supply of L-Glu for synaptic transmission whilst also allowing postsynaptic L-Glu activity to be quickly terminated [4].

L-Glu neurotoxicity is correlated with several acute and chronic neurodegenerative diseases (NDDs) such as stroke or trauma [10,11], Alzheimer’s disease [12], Parkinson’s disease [13], multiple sclerosis [14], amyotrophic lateral sclerosis [15], and Huntington’s disease [16,17]. Furthermore, animal models and human clinical studies have demonstrated that neurodegeneration correlates with pathologically raised L-Glu levels [4,10,18].

In the case of abnormal accumulation, L-Glu can initiate excessive or prolonged activation of neuronal L-Glu receptors, which trigger intracellular signaling cascades that result in cell death [4,19,20,21]. As an alternative to receptor-mediated excitotoxicity, L-Glu toxicity can also be caused by non-receptor mediated oxidative stress, and both of these mechanisms are possible within neurons and can act synergistically [22,23,24]. Ionotropic receptor-mediated excitotoxicity occurs via L-Glu overactivation of α-amino-3-hydroxy-5-methyl-4-isoxazolepropionic acid receptor (AMPAR), N-methyl-D-aspartate (NMDAR), and kainic acid receptors (KARs), and this results in an excessive influx of extracellular calcium (Ca^2+^), together with Ca^2+^ release triggered from intracellular stores, resulting in increased cytosolic free Ca^2+^ and the initiation of a cascade-like effect leading to cell death [23,25,26,27]. Furthermore, L-Glu stimulates metabotropic glutamate receptors (mGluRs) that can increase the synthesis of inositol triphosphate (IP_3_), and this can increase the release of Ca^2+^ from endoplasmic reticulum (ER) stores [23]. Additionally, excessive L-Glu can indirectly trigger the activation of voltage-dependent Ca^2+^ channels (VDCC) resulting in more Ca^2+^ influx [28]. Excessive Ca^2+^ buildup induces mitochondrial malfunction and the production of pro-apoptotic proteins [19,23,29,30,31].

The other mechanism of L-Glu toxicity is via non-receptor-mediated oxidative stress, which can involve the cystine/glutamate antiporter system ($X_{c}^{-}$) [22,32]. An overabundance of extracellular L-Glu blocks the cystine/glutamate antiporter system resulting in cysteine depletion, and this is a primary component for the synthesis of the cellular antioxidant, glutathione (GSH) [22]. A reduction of GSH levels renders cells more vulnerable to reactive oxygen species (ROS) accumulation and redox stress [33]. This can contribute to oxidative L-Glu toxicity, also known as oxytosis, which causes cell death due to oxidative stress caused by the formation of free radicals [22]. A build-up of ROS disrupts mitochondrial function and intracellular Ca^2+^ homeostasis and ultimately leads to cell death [32]. Thus, targeting the pathways downstream of excessive L-Glu accumulation such as oxidative stress and mitochondrial dysfunction might reduce the adverse consequences of excessive L-Glu and its impact on neurodegeneration and stroke [34,35].

Acai berries (*Euterpe oleracea*) are small, round palm fruits that display strong antioxidant ability against ROS in vitro [36,37]. Traditional medicine has utilized different *Euterpe oleracea* plant parts to treat a number of illnesses, including fever, gastrointestinal and skin conditions, pain, and infectious diseases [38]. Furthermore, clinical, animal, and cell-based studies have also reported the potential health benefits of acai berry fruit [36,39,40].

Acai berries have neuroprotective properties [37], and berry extracts display antioxidant and anti-inflammatory characteristics, as well as the ability to preserve proteins, calcium homeostasis, and mitochondrial function [37]. Acai-berry-derived antioxidants may have the potential to prevent or treat diseases associated with pathologically high L-Glu levels such as NDDs and stroke by regulating pathological processes via neutralization of free radicals and regulation of antioxidant/pro-oxidant status, as well as reverse mitochondrial dysfunction and maintain adenosine triphosphate (ATP) and pro-apoptotic proteins levels [37,41]. In vitro experiments indicate that acai is an important agent to minimize the incidence of age-related NDDs as it efficiently decreases oxidative damage and inflammation in brain cells [36]. In addition, acai extract improves mitochondrial dysfunction induced by rotenone exposure, and it is also able to decrease cellular ROS levels and lipid peroxidation [42]. Acai pre-treatment on brain tissues subjected to neurotoxins resulted in decreased damage in brain proteins and lipids [40]. Thus, the acai berry is a promising dietary agent that may provide neuroprotection and/or prevention of the development of neuronal damage.

This study investigated the potential of acai berry extracts to provide neuroprotection against excessive L-Glu-induced cell death using the human neuroblastoma SH-SY5Y cell line. The SH-SY5Y cell line was chosen since it is a relatively homogeneous neuroblast-like cell line and has been extensively used as a model for neurotoxicity studies [43,44]. SH-SY5Y cells can also be manipulated to differentiate into cholinergic, adrenergic, or dopaminergic phenotypes [43,44]. To further substantiate research findings with alternative neurons, the viability of human cortical neuronal progenitor cells (ReNcell CX) was assessed after application of L-Glu and/or the acai berry aqueous extract. These cells are immortalized human fetal neural stem cells from the brain cortex and are a useful model for the study of NDDs [45]. The ability of acai berry extracts to prevent or limit L-Glu-induced mitochondrial damage and loss of function, and accumulation of ROS, were also examined, as well as whether L-Glu neurotoxic effects were mediated by activation of ionotropic Glu-receptors in SH-SY5Y cells using whole-cell patch-clamp assays. Experimental evidence has highlighted the importance of L-Glu receptors in cancer cell signaling and suggests that rhabdomyosarcoma cells (TE671) express active and functional L-Glu receptors [46,47,48,49]; hence, TE671 cells were used as a positive control for whole-cell patch-clamp assays. Lastly, acai berry extracts were fractionated and analyzed using liquid chromatography-mass spectrometer (LC-MS) to identify phytochemical antioxidants that may be responsible for neuroprotective effects.

## 2. Materials and Methods

### 2.1. Chemicals and Reagents

All chemicals and media were acquired from Sigma, Poole, UK unless otherwise specified. L-Glu (49621, Sigma-Aldrich, UK) stock solution was prepared by dissolving its monosodium salt monohydrate crystals in culture medium with final concentrations obtained using dilution of stock solutions in normal growth media, and pH was checked (7 to 7.5). The acai berry ethanolic extract was prepared using maceration of 300 mg/mL of commercially available freeze-dried acai berry pulp and skin powder purchased from NaturaleBio (Organic product under EU Directive 834/2007) in 70% ethanol for 48 h. The macerated sample was shaken three times daily to assist solvation. Then, the solution was filtered using a bottle top filter. Filtrates were dried at 45–50 °C for 24 h in a water bath to obtain the ethanolic dry extracts [42,50,51]. A stock solution was prepared by dissolving the dry ethanolic extract in culture media at a concentration of 2000 µg/mL and filtered using a 0.20 µm syringe filter to obtain a clear solution. An aqueous extract was prepared using methods as described in [52]. The freeze-dried acai berry pulp and skin powder were weighed and extracted by dissolving them directly in culture media with vigorous vortexing. Then, the extract was centrifuged at 400 rpm and filtered using a 0.20 µm syringe filter to obtain a clear solution. The final extract concentrations were obtained by diluting the stock solution (2000 µg/mL) in media.

### 2.2. Cell Culture and Treatments

#### 2.2.1. Human Neuroblastoma SH-SY5Y Cell Line Culture

The undifferentiated human neuroblastoma SH-SY5Y cell line (ECACC 94030304) was purchased from the European Collection of Authenticated Cell Cultures (ECACC). SH-SY5Y cells were grown in 1:1 Minimum Essential Medium Eagle (EMEM) and Ham’s F12 Nut mix (EMEM/F12) medium supplemented with 10% heat-inactivated fetal bovine serum (FBS), 2 mM L-glutamine, 1% non-essential amino acids (NEAA), 100 U/mL penicillin, and 100 U/mL streptomycin in a 95% humid atmosphere of 5% CO_2_ and 37 °C. These conditions were maintained for all the cellular assays. SH-SY5Y cells were cultured in T75 flasks (ThermoFisher scientific, Rochester, UK) until they reached 70–80% confluence at which time they were passaged by rinsing the cells with Hanks’ balanced salt solution (HBSS), and were experiments performed using cell passage numbers 13–15 to reduce morphological and genetic changes that can arise from cells that have been extensively passaged.

SH-SY5Y cells were differentiated according to published methods [53,54]. Briefly, cells were grown on poly-D-lysine (PDL) (50 µg/mL) pre-coated glass coverslips under the conditions described above and in the same media containing 10% FBS. Once cells reached 60% confluence, growth media was replaced with differentiation media consisting of 1:1 EMEM and EMEM/F12 medium containing 1% FBS, 1% NEAA, 2 mM glutamine, 1% penicillin–streptomycin solution, and 10 µM all-*trans*-retinoic acid. A light microscope was used to monitor differentiation of neurons, and this was maintained by regular replacement of differentiation media every other day for six days.

#### 2.2.2. Human Rhabdomyosarcoma Cell Line TE671 Culture

The human undifferentiated rhabdomyosarcoma cell line TE671 was obtained from the European Collection of Authenticated Cell Cultures (ECACC; catalogue no. 89071904). TE671 cells were grown as monolayer cultures in 75 cm^2^ flasks in Dulbecco’s modified Eagle’s medium (DMEM) supplemented with 10% (*v*/*v*) FBS, 2 mM glutamine, 1% penicillin–streptomycin solution at 37 °C in a humidified 5% CO_2_–95% air environment. Once cells reached 80–90% confluence, they were passaged by rinsing them with phosphate buffered saline (PBS); then they were detached by incubating with 0.25% trypsin-EDTA for 3 min at 37 °C. After that, trypsin-EDTA activity was terminated by adding an equal volume of DMEM media. In a 15 mL falcon tube, cells were harvested and centrifuged at 1000 rpm for 5 min to remove the supernatants, and the cells were resuspended in 2 mL of DMEM media. One hundred µL of cell suspension were placed on the center of a glass coverslip for whole-cell patch-clamp. Experiments were conducted using cell passages 1–5. Alternatively, when TE671 cells reached 60% confluency, they were differentiated in serum-free DMEM containing 2 mM L-glutamine, 1% Penicillin/Streptomycin, and 400 µM N6,2′-O-dibutyrylladenosine 3′,5′-cyclic monophosphate sodium salt (dbcAMP). Cells were monitored using a microscope to observe the differentiated cells, which exhibited characteristic features of neuronal subtypes.

#### 2.2.3. Human Cortical Neuronal Progenitor Cell Culture (ReNcell CX)

Undifferentiated ReNcell CX cells (SCC007) are an immortalized human neural progenitor cell line and were purchased from Merck Millipore (Poole, UK). The ReNcell CX neural stem cells were grown in maintenance medium, supplemented with 20 ng/mL fibroblast growth factor, 20 ng/mL epidermal growth factor, 100 μg/mL penicillin, and 100 μg/mL streptomycin (referred to as complete media) in a humid atmosphere of 5% CO_2_ and 95% air at 37 °C. The monolayer cultures were maintained by plating the cells at a density of 1.5 × 10^6^ cells in T75, 75 cm^2^ or 350,000 cells in T25, 25 cm^2^ plastic tissue culture flasks precoated with 20 μg/mL laminin in Dulbecco’s Modified Eagle Medium/F12. ReNcell CX cells were cultured until they reached (70–80%) confluence then detached by incubating with accutase for 3 min in the incubator. Cells were then resuspended in the maintenance medium and centrifuged at 300× *g* for 5 min to pellet the cells. Then, the supernatant was discarded, and the cells were resuspended in complete medium. The cells were counted using a Countess™ II FL automated cell counter and seeded in 96-well plates (3 × 10^4^ cells/well) in complete medium. The cells were incubated for 48 h before carrying out assays to ensure complete attachment to the plate and to obtain 70–80% confluence. In order to reduce potential morphological and genetic changes, studies were carried out using cells that had undergone 4–10 passages only.

#### 2.2.4. Cell Treatments with L-Glutamic Acid (L-Glu) and Aqueous and Ethanolic Extracts of Acai Berries

The toxicity of L-Glu to undifferentiated SH-SY5Y cells was evaluated by incubating cells with a broad concentration range of L-Glu (0.137–100 mM) for either 24 or 48 h. To evaluate the most effective concentration of acai berry extract, the SH-SY5Y cells were exposed to a broad concentration range (0.001 µg/mL–1000 µg/mL or 0.001 µg/mL–10 µg/mL) in the lactate dehydrogenase (LDH) assay under two periods of incubation, 24 and 48 h. Acai berry extract was added in combination with L-Glu concentrations of 11 mM or 100 mM to test its neuroprotective capability.

ReNcell CX cells were exposed to L-Glu at a concentration range of 0.03–100 mM for 24 and 48 h. The effect of acai berry aqueous extract was examined at concentrations of 0.0001–1000 µg/mL for 24 and 48 h. The neuroprotective activity of different concentrations of acai berry aqueous extracts in ReNcell CX cells was examined using co-application with L-Glu at 0.1 mM or 0.3 mM.

### 2.3. (3-(4,5-Dimethylthiazol-2-yl)-2,5-diphenyltetrazolium Bromide (MTT) Cell Viability Assays

The MTT assay was used to determine the cell viability of SH-SY5Y cells and ReNcell CX cells following exposure to L-Glu, acai extracts, or the combination of both agents. Briefly, cells were plated in 96-well plates (1 × 10^4^ cells/well for SH-SY5Y cells or 3 × 10^4^ cells/well for ReNcell CX cells) and after 48 hours were treated with specific compounds for predetermined times as detailed above. MTT was added to each well at a final concentration of 0.5 mg/mL, and the plates were incubated for 2 h at 37 °C and 5% CO_2_. The MTT solution with culture medium was then removed, dimethyl sulfoxide (DMSO) was added, and the plates were shaken for 5–10 min to dissolve the formazan crystals. The absorbance was measured at 570 nm with DMSO as the blank using a Varioskan™ LUX multimode microplate reader (ThermoFisher, Waltham, MA, USA). The absorbance of the control group (absence of test compound) was considered 100% of cell viability.

### 2.4. Lactate Dehydrogenase (LDH) Assay

The toxicity of L-Glu, acai berry extracts, or the combination of both was determined with the quantitative determination of LDH that was released into the cell culture media as a result of cell plasma membrane damage [55]. The level of LDH released from damaged cells into the medium was measured using an LDH cytotoxicity assay kit (C20300, Invitrogen, Thermo-Fisher Scientific, UK) according to the manufacturer’s instructions. Briefly, SH-SY5Y cells were seeded at 1 × 10^4^ cells/well and treated with L-Glu and acai berry extract as described above, with each assay point conducted in three replicates and three independent assays performed. A volume of 50 µL of the cell-free culture media from each treatment was transferred into a new 96-well plate. Then, 50 µL of reaction mixture provided by the LDH assay kit was added to each sample well and gently mixed, and the mixture was incubated at room temperature for 30 min in the dark, after which 50 µL of stop solution was added to each sample well and mixed gently. In the same plate, an LDH positive control from the kit was used. The plates were read at 490 nm and 680 nm using a spectrophotometer (Varioskan™ LUX multimode microplate reader, ThermoFisher, Waltham, MA, USA). The 680-nm absorbance value (instrument background signal) was subtracted from the 490-nm absorbance value to calculate LDH activity. The OD values for each of the treatments were normalized to the mean of the negative control, and the % LDH production was determined.

### 2.5. Adenosine 5′-Triphosphate (ATP) Bioluminescent Assay

The measurement of cellular adenosine 5′-triphosphate (ATP) content using firefly luciferase enzyme was used to indicate mitochondrial function and associated cellular bioenergetic activity of the cells [56]. In the case of loss of cell membrane integrity by injury, ATP synthesis in the cells is decreased, and endogenous ATPase is rapidly released to deplete any remaining ATP from the cytoplasm [56]. Briefly, SH-SY5Y cells were seeded at 1 × 10^5^ cells/well in 6-well plates, grown to 80–90% confluency, and then treated with L-Glu or acai berry extracts or both as detailed in Section 2.2.4. After treating the cells for the desired time, the cells were rinsed three times with ice-cold PBS before being scraped into 500 µL of Tris/EDTA assay buffer (100 mM Trizma base and 4 mM ethylenediaminetetraacetic acid (EDTA), pH 7.75) through agitation on ice for 5 min then scraping the cells into the buffer. The isolated cell suspension was then added to 9 mL of boiling Tris/EDTA buffer and incubated at 100 °C for 10 min. Samples were then centrifuged at 1000× *g*, and the supernatant was transferred to a new Eppendorf and kept on ice until needed. ATP levels were determined in control and treated SH-SY5Y cells using an ATP Bioluminescence Assay Kit, CLS II (11699695001, Sigma-Aldrich, Poole, UK). As described in the manufacturer’s instructions, ATP standards were prepared over a concentration range of 1 × 10^−5^ to 1 × 10^−10^ M. Assay samples (100 µL volume) were transferred into a white opaque 96-well microplate for quantitation, 100 µL of luciferase reagent was added to each well, and then well luminescence was measured using a Varioskan™ LUX multimode microplate reader (ThermoFisher, Waltham, MA, USA) with an integration time of 1 s. The ATP content in control and treated samples was interpolated from the ATP standard curve. Lysis assay buffer alone was used as a blank, with values subtracted from the test samples. The corrected luminometric measurements from test samples were normalized to the mean of the control and expressed as a percentage relative to the negative control. Three separate experiments were carried out for each assay data point, and each assay was performed in triplicate.

### 2.6. Measurements of Mitochondrial Membrane Potential (MMP) Assay

The MitoTracker^®^ probe, a cell-permeable fluorescent dye that accumulates in mitochondrial membranes, was used to determine functional mitochondria. The measurement of functionally active mitochondrial membranes was performed with minor modifications [57]. SH-SY5Y cells were seeded in 96-well plates at 1 × 10^4^ cells/well and grown to 80–90% confluency before treatment with L-Glu, acai berry extracts, or both, as described in Section 2.2.4. After the cells were treated for 24 or 48 h, media were removed, and cells were incubated with a staining solution of 50 nM MitoTracker^®^ Green FM (7514, ThermoFisher Scientific, Eugene, OR, USA) at 37 °C for 30 min. The staining solution was removed, and fresh PBS was added. The fluorescence was measured using a Varioskan™ LUX multimode microplate reader (ThermoFisher, Waltham, MA, USA), using 490 nm excitation and 516 nm emission filters. Stained untreated cells with 50 nM MitoTracker^®^ Green FM were used for negative controls, and wells with non-stained cells were used as blanks. A positive control, 300 µM carbonyl cyanide-4-(trifluoromethoxy) phenylhydrazone (FCCP), was incubated with cells for 24 h to induce membrane uncoupling. Fluorescent measurements were calculated after a subtraction of the means of blank values for each treatment and normalized to the mean of the negative control and expressed as a percentage.

### 2.7. 2,7-Dichlorodihydrofluorescein Diacetate (DCFDA) Assay

The generation of cellular ROS was used as an indicator of neurotoxicant-induced oxidative stress. Neurons exposed to L-Glu, acai berry extracts, or a combination were assayed for ROS production. SH-SY5Y cells were seeded at 1 × 10^4^ cells/well in 96-well black edge plates and grown to 80–90% confluency. Cells were incubated with L-Glu and acai berry extracts at concentrations detailed above, and then 50 μM DCFDA fluorescent dye was added in normal media for 3 h at 37 °C. Hydrogen peroxide (H_2_O_2_) at 500 μM for 30 min was used as a positive control for ROS generation, stained untreated cells with 50 μM DCFDA were used as a negative control, and non-stained cells were used as blanks. Cells were then washed with PBS, and the florescence signals emitted at 535 nm with a 485 nm excitation were measured in PBS using a Varioskan™ LUX multimode microplate reader (ThermoFisher, Waltham, MA, USA). The treatment and control measurements were corrected via subtraction of the blank and normalized to the mean value from the negative control wells. ROS production was expressed as a percentage relative to the negative control. Data were generated from three separate experiments with each assay point performed in triplicate.

### 2.8. Whole-Cell Patch-Clamp Assay

Whole-cell patch-clamp electrophysiology was performed on SH-SY5Y cells at a holding potential of −50 mV using an Axopatch 200A patch-clamp amplifier (Axon Instruments, USA) and recorded to a PC disk using a data acquisition device (National Instruments, NI PCI-6221/BNC-2110) and WinEDR V3.9.1 software (Dr. John Dempster, Institute of Pharmacy and Biomedical Sciences, University of Strathclyde, Glasgow, UK). SH-SY5Y cells were placed in a perfusion chamber and continuously perfused with mammalian Ringer (135 mM NaCl, 5.4 mM KCl, 1 mM CaCl_2_, 5 mM 4-(2-Hydroxyethyl)piperazine-1-ethanesulfonic acid (HEPES), 10 mM D-glucose, pH 7.4 with NaOH) at a flow rate of ~5 mL/min. A programmable micropipette puller (P-97, Sutter Instrument Co., US) was used to create patch-pipettes using borosilicate glass capillaries (1B150F-4, World Precision Instruments, UK). A solution of 140 mM caesium chloride (CsCl), 11 mM ethylene glycol-bis(β-aminoethyl ether)-N,N,N′,N′-tetraacetic acid (EGTA), 1 mM CaCl_2_, 5 mM NaCl, 5 mM HEPES, and pH 7.2 with CsOH, was used to fill patch-pipettes, creating resistances of ~5 MΩ. A DAD-12 Superfusion system (Adams & List Associates, New York, NY, USA) was used to perfuse 3 mM L-Glu + 10 µM glycine (Gly) solution as 1–2 s pulses. Compressed nitrogen was used to pressurize the perfusion system, and solutions were applied at a pressure of 200 mm/Hg. As a positive control, 400 µM N6,2′-O-Dibutyrylladenosine 3′,5′-cyclic monophosphate sodium salt (dbcAMP)-differentiated TE671 cell inward currents were evoked by application of 3 mM L-Glu + 10 µM Gly [48]. An amount of 20 cells were examined for each cell line.

### 2.9. Liquid-Chromatography Mass-Spectrometry (LC-MS)

Acai berry aqueous and ethanolic extractions were prepared as described in a previous publication [50], but the initial concentration used was 500 mg/mL. Final products were prepared for analysis in methanol at a concentration range of 10–300 mg/mL. Acai berry extracts were analyzed using a Dionex UltiMate 3000 high-performance liquid chromatography (HPLC) system coupled to a Q-Exactive Plus hybrid quadrupole-Orbitrap mass spectrometer with a heated electrospray ionization (HESI) source (Thermo Fisher Scientific, Hemel Hempstead, UK) as previously described [58]. Injection volumes of 10 μL were used for sample analysis and were maintained at 4 °C during the analysis. The samples were injected for chromatographic separation into a ZIC-pHILIC column (150 × 4.6 mm; 5 µm particle size; Merck SeQuant, Darmstadt, Germany) while the temperature was maintained at 45 °C. Mobile phase A was composed of 20 mM ammonium carbonate in water (pH 9.1), and mobile phase B was 100% acetonitrile. The initial mobile phase was 20% A with a flow rate of 300 µL/min, which increased to 95% A after 8 min. After 11 min, the flow rate was raised to 400 µL/min, and the mobile phase A proportion was reduced to 20%. The proportion of solvent A was then maintained for re-equilibration, and between 13–14 min, the flow rate was decreased again to 300 µL/min.

Full MS profiling with simultaneous ESI+ and ESI– switching was used over the *m*/*z* range of 70–1050 and at a resolution of 70,000. The probe and capillary temperatures were maintained at 150 and 275 °C, respectively. The following setups were employed: sheath gas 40, auxiliary gas 5, sweep gas 1, 1 × 10^6^ automatic gain control (AGC) target, and maximum injection time 100 ms. The positive and negative modes of ionization used were with a spray voltage of +4 kV or −4 kV, respectively. Parallel reaction monitoring (PRM) data-independent tandem MS/MS spectra were produced on the ions in the inclusion list, which consisted of all of the compounds of interest in both positive and negative ionization mode at a resolution of 17,500, with AGC target 2 × 10^5^, maximum injection time 100 ms, normalized collision energy 35, isolation window 4.0 *m*/*z*, and default charge state 1 (Xcalibur 4.2.28.14 (Thermo Fisher Scientific, Hemel Hempstead, UK)). The compounds in the acai berry extracts were identified using Compound Discoverer 3.3 software (Thermo Scientific, UK) by matching accurate masses in the PlantCyc database, the retention times and accurate masses in the authentic standards (within 0.5 min shift), and MS^2^ fragmentation patterns in the mzCloud database.

### 2.10. Statistical Analysis

Results were expressed as means ± standard error of mean (SEM) in each of the control and treatment groups. Non-linear regression analysis using PRISM v 7.04 (GraphPad Software Inc., San Diego, CA, USA) was used to calculate the concentration of L-Glu producing 50% of maximum inhibitory (IC_50_) effects. Statistical analysis comparing different groups was performed using one-way ANOVA tests with Tukey’s or Dunnett’s multiple comparisons post-test using PRISM v 7.04 (GraphPad Software Inc., San Diego, CA, USA). A *p* value of <0.05 was defined as the level of statistical significance for all analyses.

## 3. Results

### 3.1. Exposure of Neuronal Cells to L-Glu Reduces Cell Viability

**Figure 1:**
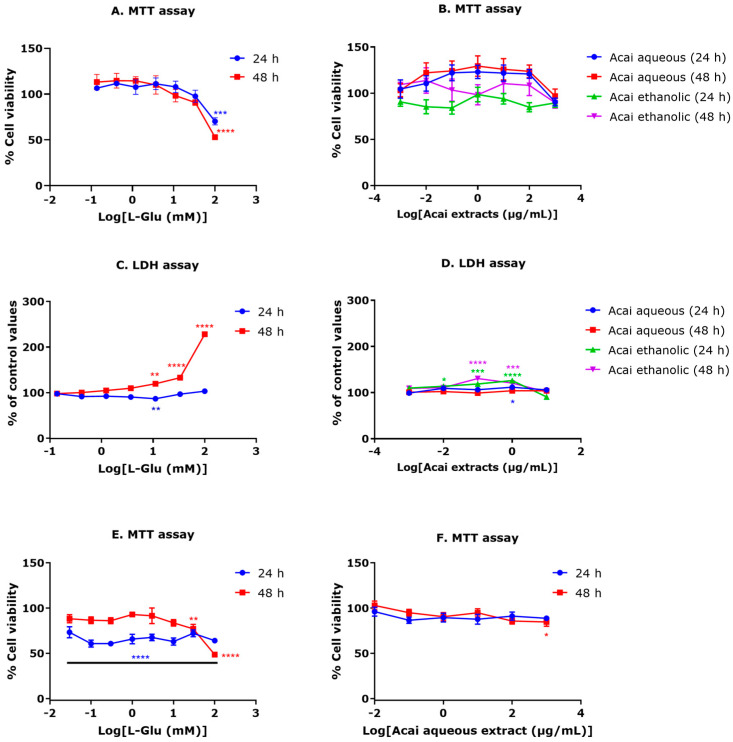
Cytotoxicity of L-Glu and acai berry extracts. SH-SY5Y cells were treated with L-Glu or acai berry extracts for 24 or 48 h and cytotoxicity determined using MTT (**A**,**B**) or LDH assays (**C**,**D**). ReNcell CX cells were treated with L-Glu (**E**) or acai berry aqueous extract (**F**) for 24 or 48 h. Data points are means ± SEM of three independent experiments (*n* = 3). Statistically significant changes were assessed using one-way ANOVA tests with Dunnett’s multiple comparisons post-test. For significance marked on Figures: * *p* < 0.05, ** *p* < 0.01, *** *p* < 0.001, and **** *p* < 0.0001 vs. control untreated cells.

Exposure of SH-SY5Y cells to L-Glu at a concentration range of 0.137–100 mM for 24 or 48 h reduced cell viability from 11.1 mM, but this did not reach significance until 100 mM. Cell viability was significantly reduced after a 100 mM L-Glu exposure for 24 h (29.7% reduction, *p* < 0.001) and 48 h (47% reduction, *p* < 0.0001) (Figure 1A). The IC_50_ for L-Glu treatment was estimated as 148.6 mM after 24 h and 93.16 mM after 48 h. Interestingly, low L-Glu concentrations of 0.137–11.11 mM for 24 h and 0.137–3.7 mM for 48 h promoted a moderate but insignificant increase (approximately 10–14%) in absorbance values for the MTT assay compared to vehicle-treated cells, consistent with increased cell proliferation (Figure 1A).

In general, 24 and 48 h exposures to acai berry aqueous or ethanolic extracts were not toxic to neuronal cells at concentrations ranging from 0.001–1000 µg/mL (Figure 1B). Relatively low concentrations of aqueous extracts (0.01–100 µg/mL) triggered increased absorbance values in MTT assays, indicative of increased cell proliferation, although this did not reach significance (Figure 1B).

The neurotoxicity of L-Glu was confirmed via the induction of extracellular LDH (Figure 1C). After a 24 h exposure, L-Glu increased LDH release at a concentration of 100 mM, and after a 48 h exposure, L-Glu at 11.1, 33.3, and 100 mM induced significantly increased LDH production of 19% (*p* < 0.001), 32% (*p* < 0.0001), and 128% (*p* < 0.001), respectively (Figure 1C).

Mostly, acai berry extracts were not toxic to SH-SY5Y cells; however, a slight increase of LDH production (approximately 11%) from control levels was observed after a 24 h exposure to 1 µg/mL of the aqueous extract (Figure 1D). Likewise, the acai berry ethanolic extract significantly increased LDH release at concentrations of 0.01 to 1 µg/mL after 24 h exposures (13–20% increase) and at 0.1 and 1 µg/mL after 48 h (30 and 20% increase, respectively) (Figure 1D).

An MTT assay was performed to assess the cytotoxic effects of L-Glu on ReNcell CX cell viability. L-Glu treatments (0.03–100 mM) for 24 h caused a significant decrease in cell viability of approximately 27–40% when compared to controls (Figure 1E). By comparison, there were no significant effects of 0.03–10 mM L-Glu concentrations on survival after 48 h when compared with controls. Cell viability declined after a 48 h exposure to 30 and 100 mM L-Glu by approximately 23% and 51%, respectively. L-Glu treatment resulted in an approximate IC_50_ of 86.88 mM after 24 h and 88.67 mM after 48 h.

**Figure 2:**
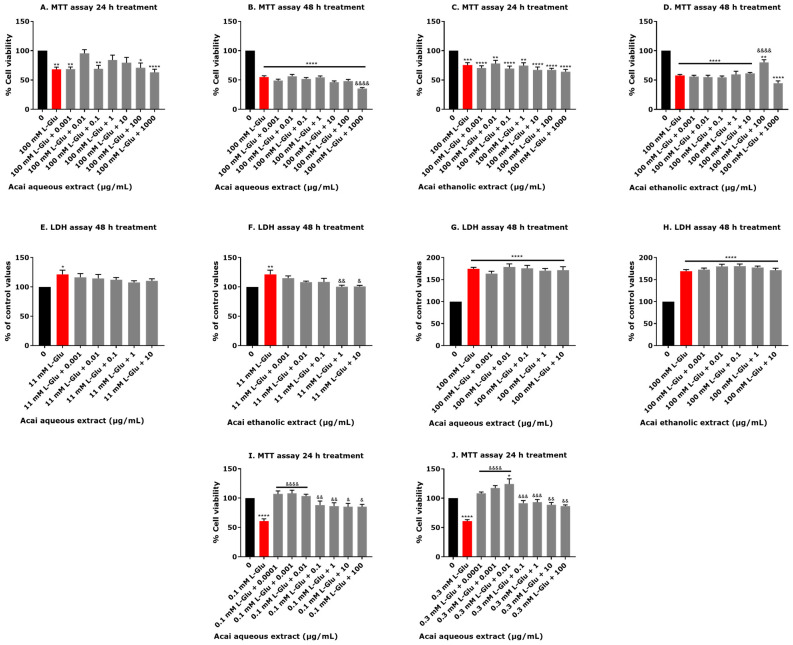
Neuroprotective effects of acai berry extracts. The cytotoxicity of 11 or 100 mM L-Glu in combination with acai berry extracts to undifferentiated SH-SY5Y cells were determined after 24 or 48 h using MTT (**A**–**D**) or LDH assays (**E**–**H**). Acai berry aqueous extract was applied in combination with L-Glu at concentrations of 0.1 mM (**I**) and 0.3 mM (**J**) over the course of 24 h in ReNcell CX cells. Data points are means ± SEM of three independent experiments (*n* = 3 per experiment). Statistically significant changes were assessed using one-way ANOVA tests followed by Tukey’s multiple comparisons post-test. For significance marked on Figures: * *p* < 0.05, ** *p* < 0.01, *** *p* < 0.001, **** *p* < 0.0001 vs. control in the absence of L-Glu and acai extract. & *p* < 0.05, && *p* < 0.01, &&& *p* < 0.001, and &&&& *p* < 0.0001 vs. L-Glu alone.

The cell viability response of ReNcell CX cells to different concentrations (0.01–1000 µg/mL) of acai berry aqueous extract was analyzed using an MTT assay. Generally, 24 and 48 h exposure to acai berry aqueous extract exhibited no toxic impacts on neuron cells at concentrations ranging from 0.01 µg/mL–100 µg/mL (Figure 2B). However, application of acai berry aqueous extract to ReNcell CX cells at 1000 µg/mL for 24 h caused a non-significant decrease of approximately 12% in neuron viability, and this reached a significant 16% reduction after a 48 h exposure (Figure 1F).

### 3.2. Certain Concentrations of Acai Berry Extracts Provide Neuroprotection against L-Glu-Induced Decreased Cell Viability

The neuroprotective capability of acai berry aqueous and ethanolic extracts against neurotoxicity induced by 100 mM L-Glu exposure for 24 and 48 h were evaluated using an MTT assay in SH-SY5Y cells. Acai berry aqueous extract at concentrations of 0.01, 1, and 10 µg/mL increased cell viability by 11–27% after a 24 h exposure (Figure 2A). The acai aqueous extract at concentrations ranging from 0.001–100 µg/mL was not neuroprotective against L-Glu after a 48 h exposure, and the highest concentration of acai berry aqueous extract (1000 µg/mL) caused a further reduction in cell viability by approximately 20% when compared with L-Glu only (Figure 2B). Acai ethanolic extracts were not protective against 100 mM L-Glu after 24 h; however, after 48 h of exposure, 100 µg/mL significantly protected against L-Glu neurotoxicity and increased cell viability by 23% (*p* < 0.0001) (Figure 2C,D).

LDH levels were not significantly affected by L-Glu exposure after 24 h (results not included); therefore neuroprotection experiments at this time point were not conducted. There was a concentration-dependent inhibition of 11 mM L-Glu-induced LDH production after a 48 h co-application with both acai aqueous or ethanolic extracts (Figure 2E,F). The acai berry ethanolic extract co-incubated with L-Glu caused a significant reduction of LDH production induced by L-Glu of approximately 20% at concentrations of 1 and 10 µg/mL (*p* < 0.01 and *p* < 0.05, respectively) (Figure 2F). However, neither of the acai berry extracts was neuroprotective against the higher concentration of 100 mM L-Glu (Figure 2G,H).

Acai berry aqueous extracts (0.0001 µg/mL–100 µg/mL) exhibited protection from the toxicity of 0.1 or 0.3 mM L-Glu for 24 h in ReNcell CX cells. All concentrations of acai berry extract significantly increased cell viability by approximately 15–40% after a 24 h exposure when compared with 0.1 mM L-Glu alone (Figure 2I). Likewise, significant increases in neuron viability were observed after the co-incubation of acai berry aqueous extracts (0.0001–100 µg/mL) with 0.3 mM L-Glu for 24 h, with a concentration-dependent increase in cell viability observed over the concentration range of 0.0001–0.01 µg/mL (Figure 2J).

### 3.3. Acai Berry Extracts Preserve ATP Levels That Were Diminished after L-Glu Treatment

**Figure 3:**
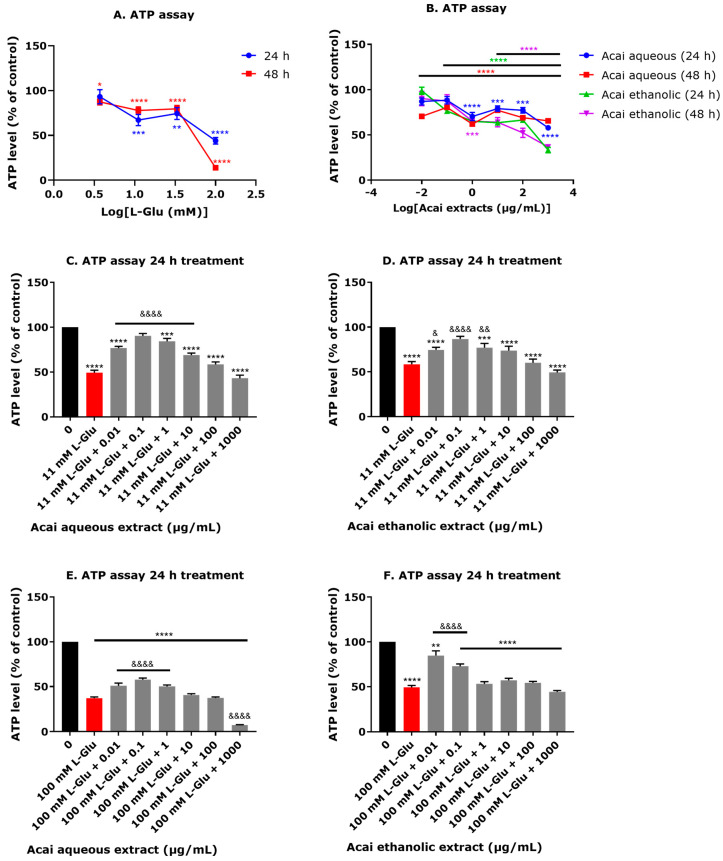
The effects of L-Glu and acai berry extracts on ATP levels. SH-SY5Y cells were exposed to L-Glu (**A**) or acai berry extracts (**B**) for 24 or 48 h or exposed to 11 or 100 mM L-Glu and different concentrations of acai berry extracts for 24 h (**C**–**F**), and ATP levels quantified. Values were normalized to the negative control providing an ATP level percentage relative to the vehicle control. Results were expressed as the mean ± SEM for three separate experiments at each concentration (*n* = 3 per experiment) and analyzed using one-way ANOVA with Tukey’s multiple comparisons. For significance marked on Figures: * *p* < 0.05, ** *p* < 0.01, *** *p* < 0.001, and **** *p* < 0.0001 vs. control (0) in the absence of L-Glu and acai berry extracts. & *p* < 0.05, && *p* < 0.01, and &&&& *p* < 0.0001 vs. L-Glu alone.

The application of L-Glu at 11.1–100 mM for 24 h caused significant reductions in cellular ATP levels, with a 33–55% decline and an estimated IC_50_ of 85.72 ± 26.82 mM (Figure 3A). A 48 h incubation with L-Glu induced a higher depletion of cellular ATP and one initiated at a lower concentration of L-Glu (3.7 mM) to 87.3% ± 3.3 (*p* < 0.05), and this was reduced to 13.9% ± 0.64 (*p* < 0.0001) at the higher concentration of 100 mM L-Glu, with an estimated IC_50_ of 52.17 ± 4.26 mM (Figure 3A). A slight reduction in ATP levels was observed after acai berry extract application (Figure 3B). A 24 h exposure to acai berry aqueous extract at 0.01 and 0.1 µg/mL had no effect on ATP levels, but after 48 h these caused a significant reduction in ATP levels by approximately 30% (Figure 3B). Acai berry ethanolic extract similarly reduced ATP levels at concentrations above 0.01 μg/mL for a 24 h exposure or above 0.1 μg/mL after 48 h (Figure 3B).

Generally, acai berry extracts provided protection from the decline in ATP levels caused by 11 or 100 mM L-Glu. Greater protection from 11 mM L-Glu was observed with the acai aqueous extract at the relatively low concentration range of 0.01–10 µg/mL, which significantly increased ATP levels by approximately 20%, and similarly, the acai ethanolic extract preserved ATP levels over the concentration range of 0.01–1 µg/mL (Figure 3C,D). Protection against 100 mM L-Glu was evident with the acai berry aqueous extract at concentrations ranging from 0.01–1 µg/mL and with the acai berry ethanolic extract at concentrations of 0.01 and 0.1 µg/mL (Figure 3E,F). However, the highest concentration of the acai berry aqueous extract (1000 µg/mL) further reduced ATP levels (Figure 3E).

### 3.4. Acai Berry Extracts Restored the MMP Level That Was Reduced after L-Glu Treatment

**Figure 4:**
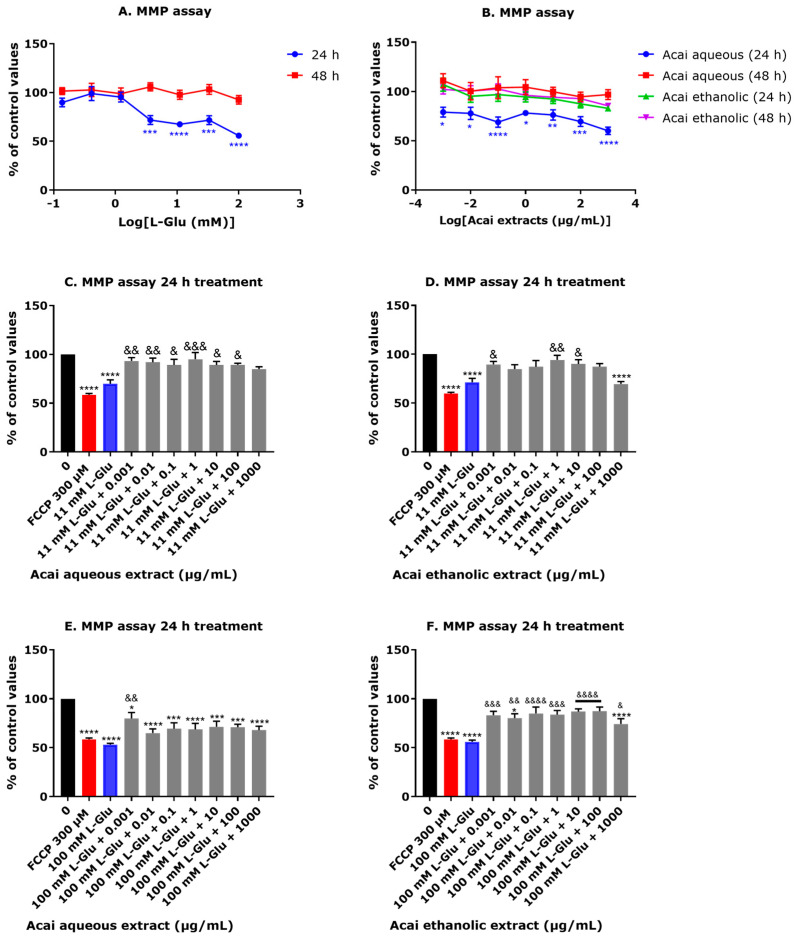
The effects of L-Glu and acai berry extracts on MMP levels. SH-SY5Y cells were exposed to L-Glu (**A**) or acai berry extracts (**B**) for 24 or 48 h or exposed to 11 or 100 mM L-Glu with different concentrations of acai berry extracts for 24 h (**C**–**F**), and the MMP level quantified. As a positive control, cells were treated for 24 h with 300 µM FCCP. Data values presented are means ± SEM for three separate experiments at each concentration (*n* = 3 per experiment) and analyzed using one-way ANOVA with Tukey’s multiple comparisons. For significance marked on Figures: * *p* < 0.05, ** *p* < 0.01, *** *p* < 0.001, and **** *p* < 0.0001 vs. control (0) in the absence of L-Glu, acai berry extracts and FCCP. & *p* < 0.05, && *p* < 0.01, &&& *p* < 0.001, and &&&& *p* < 0.0001 vs. L-Glu alone.

L-Glu at concentrations of 3.7–100 mM for 24 h caused a significant reduction of MMP values whereas an extension of the incubation period to 48 h resulted in a return to control values (Figure 4A). For the acai berry extracts, only the aqueous extracts (0.001–1000 µg/mL) reduced the MMP (by approximately 30% after 24 h), but values returned to control levels after a 48 h exposure (Figure 4B). Acai berry extracts were protective against the reduced MMP in response to 11 and 100 mM L-Glu. A 24 h exposure of the acai berry aqueous extract at concentrations of 0.001–100 µg/mL significantly restored MMP to control levels when compared to 11 mM L-Glu alone (Figure 4C). Likewise, the acai berry ethanolic extract restored the MMP to approaching control levels after a 24 h 11 mM L-Glu treatment, with significant changes at concentrations of 0.001, 1, and 10 µg/mL (Figure 4D). For reductions of the MMP after 100 mM L-Glu, only the lowest concentration (0.001 µg/mL) of the acai berry aqueous extract resulted in a significant restoration of the MMP level (Figure 4E), whereas all the acai berry ethanolic extract concentrations showed protection against 100 mM L-Glu effects on the MMP (Figure 4F).

### 3.5. Acai Berry Extracts Significantly Reduced ROS Production Induced by L-Glu

**Figure 5:**
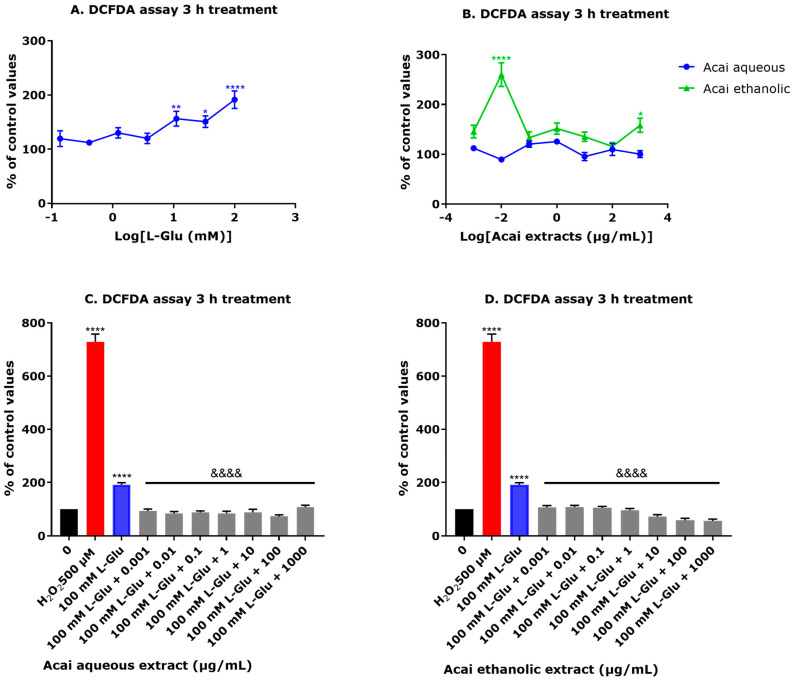
L-Glu and acai berry extract effects on cellular ROS levels. SH-SY5Y cells were treated with L-Glu (**A**), acai berry extracts (**B**), or 100 mM L-Glu with different concentrations of acai berry extracts (**C**,**D**), and the levels of ROS quantified using a DCFDA assay. Hydrogen peroxide (H_2_O_2_) at 500 μM for 30 min was used as a positive control for ROS generation. Data values were analyzed using one-way ANOVA with Tukey’s multiple comparisons and presented as means ± SEM for three separate experiments at each concentration (*n* = 3 per experiment). For significance marked on Figures: * *p* < 0.05, ** *p* < 0.01, and **** *p* < 0.0001 vs. control in the absence of L-Glu, acai berry extracts and H_2_O_2_. &&&& *p* < 0.0001 vs. L-Glu alone.

The levels of ROS induced after L-Glu exposure to SHSY-5Y cells were quantified using a DCFDA assay. Treatment of cells with L-Glu for 3 h induced ROS in a concentration-dependent way, which reached significance at concentrations of 11.1, 33.3, and 100 mM (56, 50, and 91% increase from controls, respectively) (Figure 5A). In general, an incubation with acai berry extracts for 3 h showed no increases in ROS production except for the 0.01 and 1000 µg/mL concentrations of the ethanolic extract (Figure 5B). The acai berry extracts exhibited antioxidant effects and reduced the ROS induced by L-Glu to levels similar to controls, and for the ethanolic extracts, co-exposure displayed a concentration-dependent reduction of ROS levels (Figure 5C,D).

### 3.6. Whole-Cell Patch-Clamp Assay

**Figure 6:**
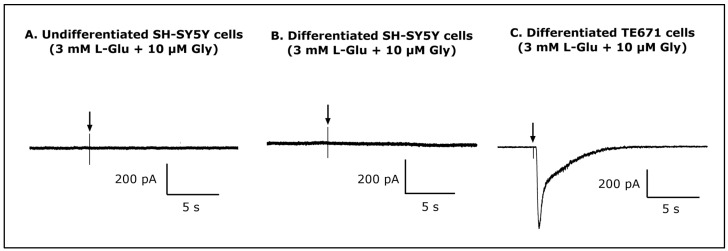
Patch-clamp recordings of whole-cell current responses. (**A**) No inward current was recorded after the application of 3 mM L-Glu + 10 μM glycine (Gly) to undifferentiated SH-SY5Y cells, or (**B**) differentiated SH-SY5Y cells. (**C**) A positive control current response was achieved from differentiated TE671 cells exposed to brief applications of 3 mM L-Glu + 10 μM Gly using whole-cell patch-clamp recordings at a holding potential of −50 mV (*n* = 20).

In order to assay for the presence of functionally active ionotropic glutamate receptors (iGluRs), undifferentiated and differentiated SH-SY5Y cells (20) were examined via the whole-cell patch-clamp technique with brief exposure to 3 mM L-Glu + 10 µM glycine (Gly). Whole-cell patch-clamp recordings were obtained at a holding potential of −50 mV in Mg^2+^-free perfusion solution. Typical inward currents did not appear following the administration of the agonists to undifferentiated or differentiated SH-SY5Y cells (Figure 6A,B). By contrast, in differentiated TE671 cells, response currents were detected after brief exposure to 3 mM L-Glu + 10 µM Gly (Figure 6C).

### 3.7. Liquid Chromatography-Mass Spectrometry (LC-MS)

LC-MS/MS analysis of acai berry extracts revealed the presence of fifty phytochemicals, including phenolic compounds, flavonoids, lignans, proanthocyanidins, monoterpenoids, and norisoprenoids, as well as fatty acids and amino acids (refer to Table 1, Supplementary Table S1). Differences in the compound profiles between phosphate buffered saline (PBS) and ethanolic extractions were evident, with improved recovery of phenolic compounds and phenolic acids and amino acids in PBS, in contrast to flavonoid and fatty acid enrichment in the ethanolic extracts (Table 1).

## 4. Discussion

The pathological process of several NDDs and stroke has been linked to abnormal over-stimulation of glutamatergic neurotransmitter systems [10,11,12,13,14,15,16,17,18]. Previous studies have reported that high concentrations of L-Glu may trigger neural death via excitotoxicity and/or oxidative injury [22,23]. In the present study, L-Glu induced a significant decline of the survival of SH-SY5Y cells at the elevated concentration of 100 mM for 24- and 48-h, as observed using an MTT assay. However, using an LDH assay, a more definitive method able to distinguish cytostatic from cytocidal effects, significant toxicity was observed at lower concentrations (from 11 mM) of L-Glu. Similarly, in the ReNcell CX cells, L-Glu (0.03–100 mM) exposure for 24 h induced a significant decrease in cell viability; however, only high concentrations of L-Glu (30 and 100 mM) significantly reduced cell viability after a 48 h exposure. L-Glu also impacted cellular bioenergetics with depletion of ATP levels and the MMP, and induction of ROS in SH-SY5Y cells. Acai berry extracts displayed little or no significant neurotoxicity and were able to counter the L-Glu neurotoxicity, with increased survival of L-Glu treated cells after 24 and 48 h. The neuroprotective character of the acai berry extracts was further validated via their ability to limit the L-Glu depletion of cellular ATP levels and MMP, as well as counter L-Glu induced redox stress, monitored as elevated ROS. Lastly, the toxicity of L-Glu was not mediated via excitotoxicity and activation of iGluRs, and this was confirmed using whole-cell patch clamping that recorded no current responses to a combined L-Glu and Gly stimulus.

Other independent studies have confirmed that L-Glu is toxic to neuronal cells including SH-SY5Y cells and induces cell death at a concentration range from 0.1–400 mM [63,64,65,66,67,68,69,70,71]. By comparison, SH-SY5Y cell incubation with the acai berry extracts alone at a concentration range of 0.01–100 µg/mL increased the absorbance values in the MTT assay. This is consistent with the presence of agents capable of stimulating cell proliferation, and this observation is supported by other studies [42,72]. Selective concentrations of the acai berry extracts, however, did retain chemical(s) that were neurotoxic under these incubation conditions, most notably the 1 µg/mL aqueous extract and concentrations of 0.01 to 1 µg/mL for ethanolic extracts, as these evoked slight increases in extracellular LDH production. Likewise, a high concentration of acai berry aqueous extract caused a minor decrease in viability of ReNcell CX cells. The rationale for why only selective concentrations of either aqueous or ethanolic extracts are able to induce mild toxicity and without a concentration response effect is yet to be established and will need to be assessed after further fractionation of the extracts. Other studies have validated acai berry extract safety [36,73,74,75]; however, similar to our findings, certain concentrations of extracts reduced cell viability [36,73,74,75]. A limited number of the berry extracts were neuroprotective against the L-Glu-induced decline in cell viability measured via MTT or LDH assays, but this was also not concentration-dependent (Figure 2A–D). However, in ReNcell CX cells, all concentrations of acai berry aqueous extract were protective against L-Glu toxicity when combined with L-Glu at 0.1 or 0.3 mM for 24 h (Figure 2I,J).

The neurotoxicity of L-Glu was in part mediated by an impact on cellular bioenergetics with reduced ATP production, consistent with other studies [65,67,70]. The decline in ATP levels correlated with L-Glu concentration and similarly, the low-level toxicity of acai berry extracts and reduced ATP levels was concentration-dependent (Figure 3A,B). Hence, neuroprotection provided by the acai extracts was predominantly provided by relatively low extract concentrations, with further toxicity evident from the co-incubation of L-Glu with the highest acai berry extract concentrations (Figure 3C–F).

L-Glu-induced damage to mitochondria was evident from lowered MMP values with 24 h effects that correlated with L-Glu concentration. Other studies have similarly demonstrated L-Glu effects on MMP levels and functionality of mitochondria [65,66,67,70,71,76,77]. Mild toxic effects of the aqueous extracts were apparent from reduced MMP levels, and these were predominantly concentration-dependent. However, the majority of the aqueous extracts and several of the ethanolic extracts were neuroprotective and able to restore the reduced MMP in response to 11 mM L-Glu, and surprisingly, the ethanolic extracts ably restored the MMP with the 100 mM L-Glu treatments (Figure 4A–F).

Damage to mitochondria promotes the release of ROS that can damage cellular proteins, lipids, and DNA and contribute to signaling for cell death [78,79]. In keeping with L-Glu-induced oxytosis, ROS levels were directly correlated with L-Glu concentration. The acai berry ethanolic extracts induced more ROS than their aqueous extract counterparts at each of the assayed concentrations, but aside from a single anomalous data point at 0.01 µg/mL and at the highest concentration of 1000 µg/mL, these did not reach significance. Both aqueous and hydroethanolic extracts of the acai berry have potent antioxidant activity [50,72,74,75], in keeping with their abilities to ameliorate the L-Glu-induced production of ROS (Figure 5A–D) and oxidative stress [40,42].

In several studies, acai berry extracts have demonstrated antioxidant properties and are able to maintain mitochondrial function, illustrating the potential neuroprotective properties of the berries [37,41,80,81]. Collectively, the acai berry aqueous and ethanolic extracts displayed differential neuroprotective effects, some of which were concentration dependent. This likely reflects differences in phytochemical recoveries between the solvents (Table 1). Highly polar solvents such as methanol and ethanol are effective at extracting phenolic compounds from plants, some of which are useful antioxidants [82]. However, the yield of polyphenols and flavonoids from acai berries was higher with water than with methanol or ethanol alone [83]. This potential for differential recovery of phytochemicals with the two solvents provides a rationale for altered neuroprotective properties; for example, the ethanolic extract provides greater protection against the loss of the MMP at the higher L-Glu treatment than the aqueous extract (Figure 4E,F). Likewise, the anomalous data point (0.01 µg/mL of the ethanolic extract) that induced relatively high levels of ROS after 3 h (Figure 5B) presumably reflects a response from a specific phytochemical(s).

The results of this study have established that L-Glu toxicity was not through excitotoxicity mediated by activation of iGluRs since no current responses to a combined L-Glu and Gly stimulus were observed. Likewise, the administration of a N-methyl-D-aspartate (NMDA) agonist could not evoke current responses in SH-SY5Y cells, suggesting that iGluRs are not expressed or not functional in these cells [84]. Furthermore, MK-801, an NMDAR antagonist, was not effective in restoring cell viability decreased by L-Glu administration, suggesting that the L-Glu cytotoxic effects arise from mechanisms other than NMDAR or other iGluRs [85]. Our patch-clamp studies considered undifferentiated and differentiated SH-SY5Y cells, and neither produced cell currents in response to L-Glu + L-Gly, and likewise, other studies using differentiated SH-SY5Y cells confirmed an absence of functional NMDA receptors [84]. Hence, it remains equivocal as to whether the human SH-SY5Y neuroblastoma cells express L-Glu receptors, since earlier studies involving this cell line suggested potentially active iGluRs and mGluRs [86,87,88,89], but differences between study results could reflect cell culturing conditions, including those employed to stimulate differentiation [43,44].

Further investigation of the phytochemical composition of the acai berry extracts was conducted using LC-MS analysis. This revealed several compounds found in acai berry PBS and/or ethanolic extracts with recognized antioxidant activities such as protocatechuic acid, syringic acid, vanillic acid, 4-hydroxybenzoic acid, chlorogenic acid, quercetin, taxifolin, quercetin-3-O-rutinoside, isoorientin, and (+)-lariciresinol [50,59,60,61,62]. Moreover, many studies have previously reported that certain phytochemicals have neuroprotective properties such as protocatechuic acid [90,91], syringic acid [91,92], vanillic acid [93], gallic acid [91], 4-hydroxybenzoic acid [94], chlorogenic acid [91], luteolin [95], quercetin [95], taxifolin [96], quercetin-3-O-rutinoside (rutin) [95], and isoorientin [97]. Furthermore, in silico studies have predicted that certain plant phytochemicals have NMDAR antagonist activity and could prevent L-Glu excitotoxicity, such as chlorogenic acid [98], quercetin, gallic acid, protocatechuic acid, and vanillic acid [99], and the LC-MS results from this study confirm that acai berry extracts contain these phytochemicals (Table 1). This implies that acai berry extracts may retain a range of phytochemicals that have antioxidant and anti-excitotoxicity effects that could be neuroprotective and reduce vulnerability to excessive L-Glu levels such as those associated with NDDs and stroke. However, these acai berry extracts and phytochemicals will require further in vivo testing in order to assess their therapeutic potential.

## 5. Conclusions

To conclude, although acai berry extracts alone exhibited mild adverse effects on SH-SY5Y cells, co-incubation of certain extracts with L-Glu was neuroprotective and limited the L-Glu-induced loss of cell viability, restored mitochondrial function, and ameliorated oxidative stress. Given the important role of excessive L-Glu accumulation and toxicity in NDDs and stroke, extraction of the active neuroprotective agent(s) from acai berry extracts may prove useful chemicals able to limit disease development and/or provide a therapeutic treatment. However, a limitation of the current study is that it is preliminary and only provides in vitro characterization data with whole plant extracts. Therefore, future individual testing of the active ingredients within acai berries, such as those identified by the LC-MS is needed to confirm the neuroprotective data. Once this has been completed and active ingredient(s) identified, experiments could be extended to in vivo experimental models, and then to consideration of agent application to humans.

## Figures and Tables

**Table 1:** LC-MS results of acai berry extracts and compound antioxidant activities.

Name of Compound	Formula	Exact Mass	Acai Berry Extracts		Antioxidant Activity
			PBS	Ethanol	
Phenolic compounds and phenolic acids:					
Protocatechuic acid	C_7_H_6_O_4_	154.0268	Detected	Detected	✓
Syringic acid	C_9_H_10_O_5_	198.0530	Detected	Detected	✓
Vanillic acid	C_8_H_8_O_4_	168.0425	Detected	Detected	✓
Gallic acid	C_7_H_6_O_5_	170.0217	Detected	Not Detected	✓
4-Hydroxybenzoic acid	C_7_H_6_O_3_	138.0319	Detected	Detected	✓
Benzoic acid	C_7_H_6_O_2_	122.0371	Detected	Not Detected	✓
2,5-Dihydroxybenzoic acid	C_7_H_6_O_4_	154.0268	Detected	Detected	✓
Chlorogenic acid	C_16_H_18_O_9_	354.0951	Detected	Detected	✓
Flavonoids:					
Dihydrokaempferol	C_15_H_12_O_6_	288.0634	Detected	Detected	✓
Luteolin	C_15_H_10_O_6_	286.0478	Not Detected	Detected	✓
Quercetin	C_15_H_10_O_7_	302.0426	Not Detected	Detected	✓
Taxifolin deoxyhexose or Taxifolin	C_15_H_12_O_7_	304.0584	Detected	Detected	✓
Quercetin-3-O-rutinoside (rutin)	C_27_H_30_O_16_	610.1519	Not Detected	Detected	✓
Quercetin 3-O-glucoside (Isoquercitrin)	C_21_H_20_O_12_	464.0950	Not Detected	Detected	✓
Kaempferol rhamnoside	C_21_H_20_O_10_	432.1048	Detected	Not Detected	✓
Isoorientin	C_21_H_20_O_11_	448.0996	Not Detected	Detected	✓
Lignans:					
(+)-Isolariciresinol	C_20_H_24_O_6_	360.1575	Detected	Detected	✓
(+)-lariciresinol	C_20_H_24_O_6_	360.1575	Detected	Detected	✓
Dihydroconiferyl alcohol	C_10_H_14_O_3_	182.0945	Detected	Detected	-
Proanthocyanidins:					
(+)-Catechin	C_15_H_14_O_6_	290.0785	Not Detected	Detected	✓
Monoterpenoids:					
(+)-menthiafolic acid	C_10_H_16_O_3_	184.1100	Detected	Not Detected	-
(E,Z)-2,6-dimethyl-2,6-octadiene-1,8-diol	C_10_H_18_O_2_	170.1309	Not Detected	Detected	-
Norisoprenoids:					
(-)-loliolide	C_11_H_16_O_3_	196.1099	Not Detected	Detected	✓
Major fatty acids:					
Monounsaturated fatty acids					
Oleic acid	C_18_H_34_O_2_	282.2558	Detected	Detected	✓
Palmitoleic acid	C_16_H_30_O_2_	254.2247	Not Detected	Detected	-
Polyunsaturated fatty acids					
Linoleic acid	C_18_H_32_O_2_	280.2401	Not Detected	Detected	✓
Linolenic acid	C_18_H_30_O_2_	278.2245	Not Detected	Detected	✓
Saturated fatty acids					
Palmitic acid	C_16_H_32_O_2_	256.2403	Not Detected	Detected	-
Stearic acid	C_18_H_36_O_2_	284.2714	Not Detected	Detected	-
Amino acids:					
Alanine	C_3_H_7_NO_2_	89.0477	Detected	Detected	-
Lysine	C_6_H_14_N_2_O_2_	146.1055	Detected	Not Detected	✓
Arginine	C_6_H_14_N_4_O_2_	174.1116	Not Detected	Detected	✓
Methionine	C_5_H_11_NO_2_S	149.0511	Detected	Not Detected	✓
Phenylalanine	C_9_H_11_NO_2_	165.0790	Detected	Detected	-
Proline	C_5_H_9_NO_2_	115.0632	Detected	Detected	-
Glutamic acid	C_5_H_9_NO_4_	147.0532	Detected	Detected	-
Serine	C_3_H_7_NO_3_	105.0426	Detected	Detected	-
Glycine	C_2_H_5_NO_2_	75.0320	Detected	Detected	-
Threonine	C_4_H_9_NO_3_	119.0582	Detected	Detected	-
Histidine	C_6_H_9_N_3_O_2_	155.0694	Detected	Detected	✓
Tryptophan	C_11_H_12_N_2_O_2_	204.0901	Detected	Detected	✓
Tyrosine	C_9_H_11_NO_3_	181.0741	Detected	Detected	✓
Isoleucine	C_6_H_13_NO_2_	131.0946	Detected	Detected	-
Valine	C_5_H_11_NO_2_	117.0788	Detected	Detected	-
Leucine	C_6_H_13_NO_2_	131.0948	Not Detected	Detected	-
Other compounds:					
Cellotetraose	C_24_H_42_O_21_	666.2225	Not Detected	Detected	-
Sucrose	C_12_H_22_O_11_	342.1155	Detected	Detected	✓
Quinic acid isomer 1	C_7_H_12_O_6_	192.0635	Detected	Detected	✓
Ascorbic acid (Vitamin C)	C_6_ H_8_ O_6_	176.0322	Detected	Detected	✓

Abbreviation: PBS, phosphate buffered saline; (-), no data; (✓) Recognized antioxidant activity [50,59,60,61,62].

## Data Availability

Data is available on request from the first author.

## Supplementary Materials

The following supporting information can be downloaded at: https://www.mdpi.com/article/10.3390/life13041019/s1, Figure S1: LC-MS profiling for phytochemicals from acai berry aqueous and ethanolic extracts; Table S1: Identificaition of major phytochemicals from acai berry aqueous and ethanolic extracts.
