# Supplementary material for: Acai Berry (Euterpe sp.) Extracts Are Neuroprotective against L-Glutamate-Induced Toxicity by Limiting Mitochondrial Dysfunction and Cellular Redox Stress
Source: Life (Basel). 2023 Apr 15;13(4):1019. doi: 10.3390/life13041019 (PMC10144606; doi:10.3390/life13041019)
Supplement: Supplementary file 1 [file life-13-01019-s001.zip › Supplementary Figure S1.pdf]

Supplementary Figure S1: LC-MS profiling for phytochemicals from acai berry aqueous and ethanolic extracts.

Acai berry aqueous and ethanolic extractions were resolved by high performance liquid chromatography coupled to mass spectrometer (LC-MS). Compounds were identified by comparing the retention time, parent ion, and mass fragments with authentic standards and MS^2 fragmentation patterns.

Compound	Chromatogram and mass spectrum
Protocatechuic acid	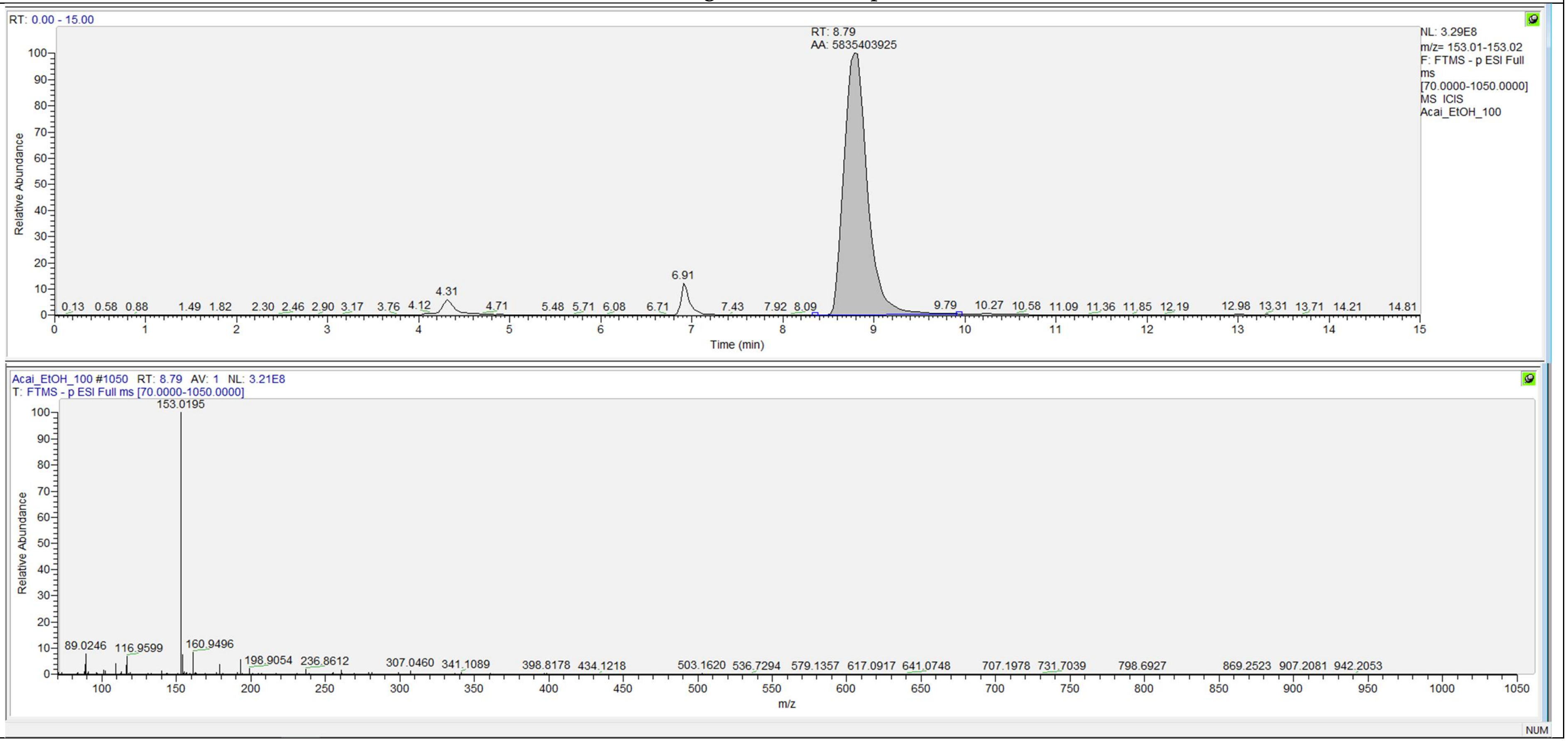

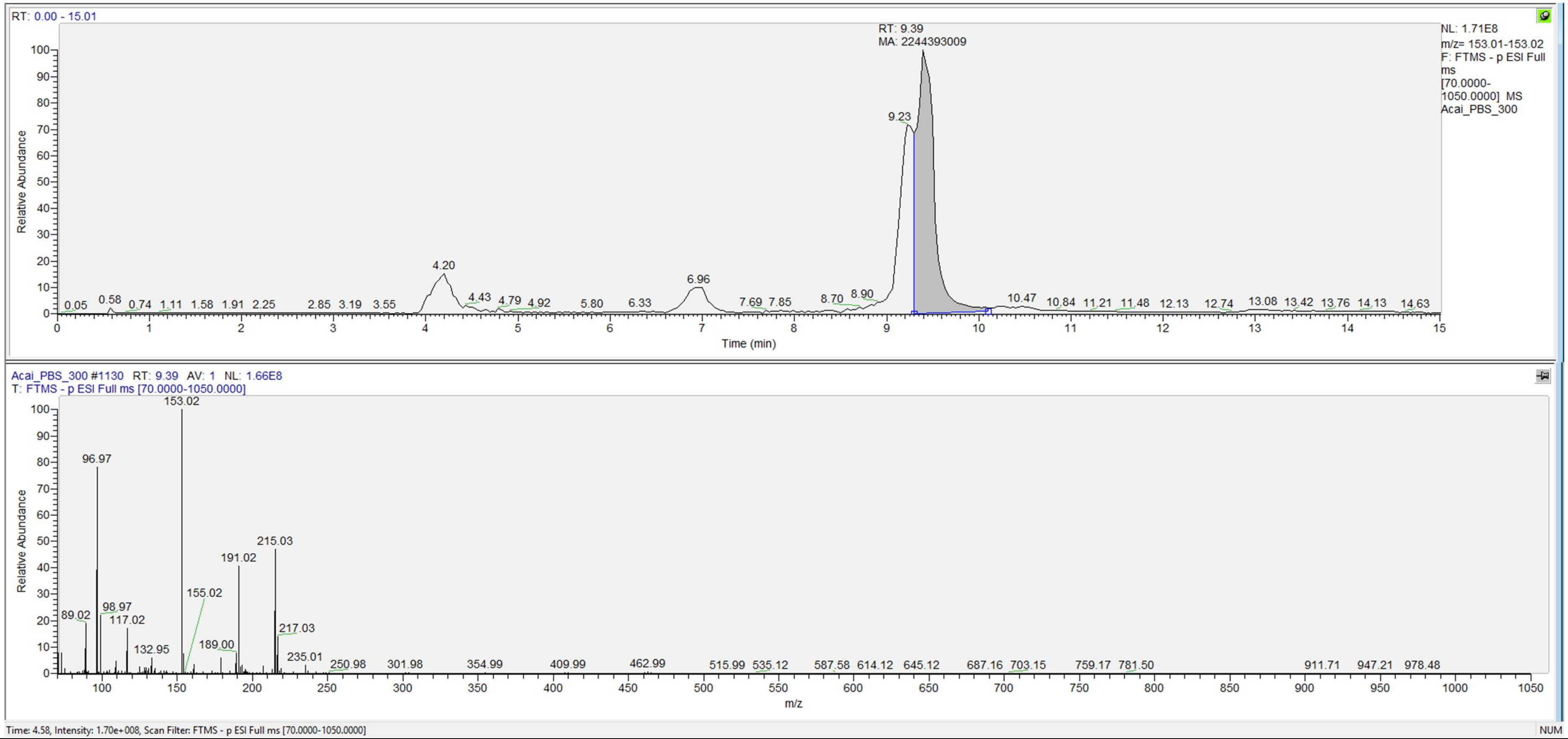
RT: 0.00 - 15.01
RT: 9.39
MA: 2244393009
NL: 1.71E8
m/z= 153.01-153.02
F: FTMS - p ESI Full ms [70.0000-1050.0000] MS
Acai_PBS_300
Relative Abundance
Time (min)
Acai_PBS_300 #1130 RT: 9.39 AV: 1 NL: 1.66E8
T: FTMS - p ESI Full ms [70.0000-1050.0000]
153.02
96.97
215.03
191.02
155.02
89.02
98.97
117.02
217.03
132.95
189.00
235.01
m/z
Time: 4.58, Intensity: 1.70e+008, Scan Filter: FTMS - p ESI Full ms [70.0000-1050.0000]

Syringic acid	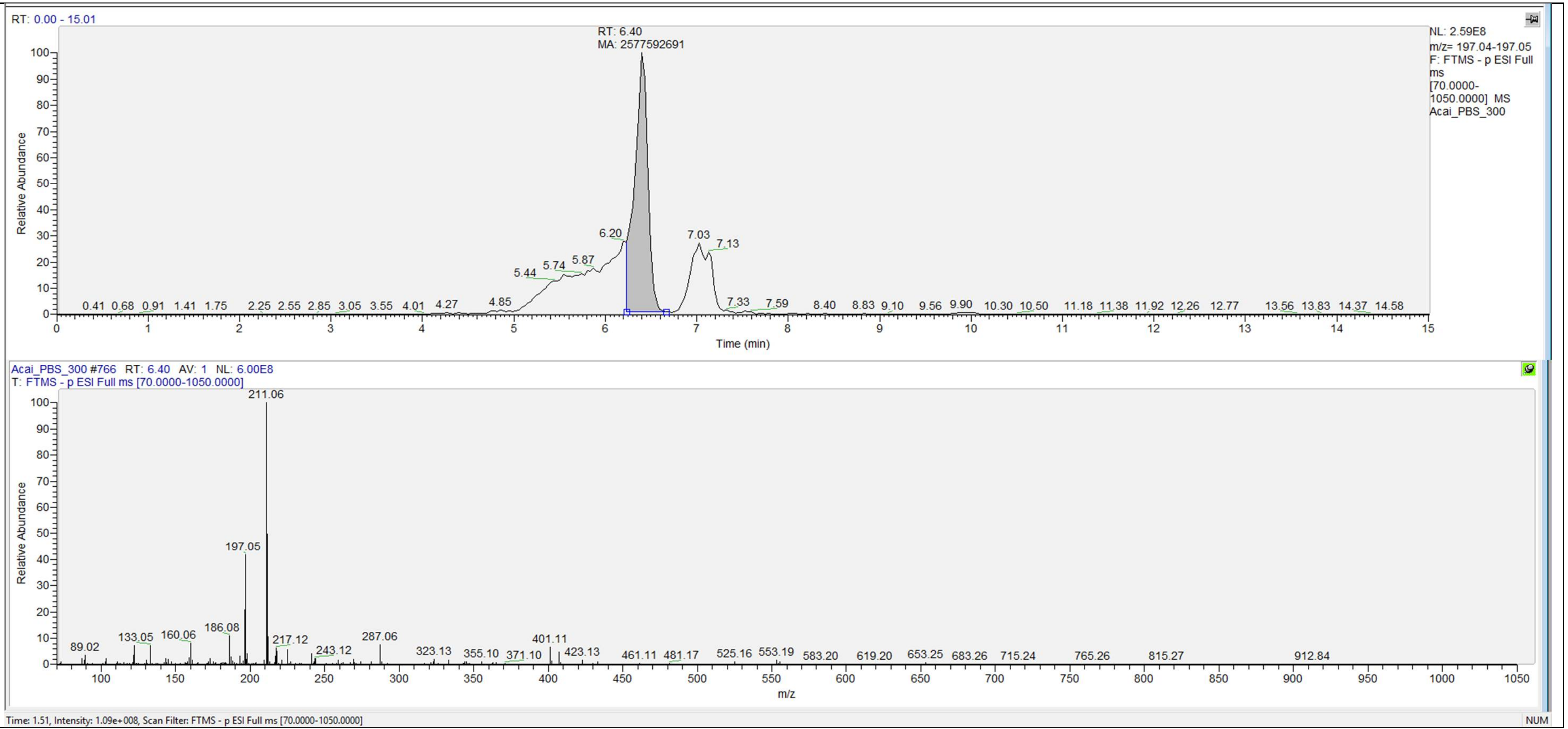

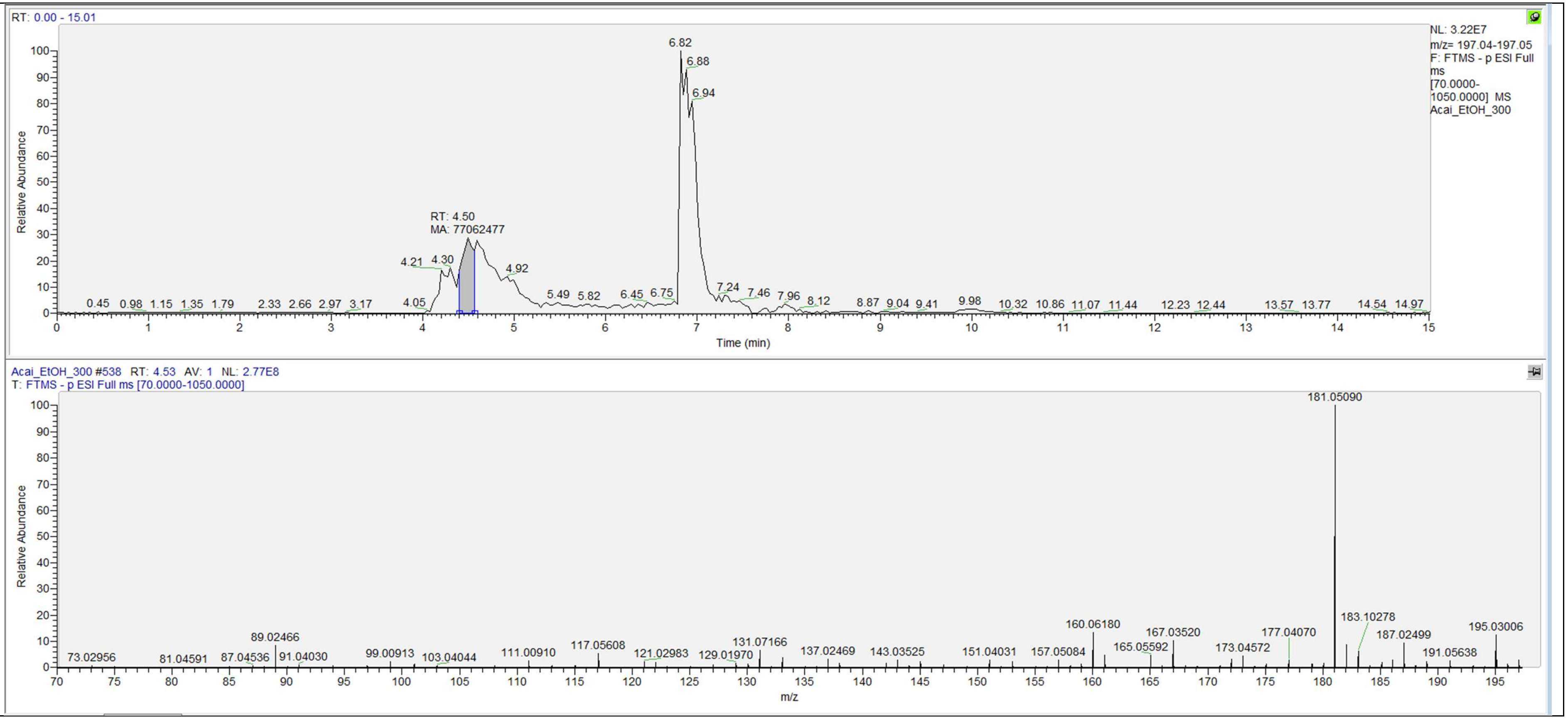

RT: 0.00 - 15.01
NL: 3.22E7
m/z= 197.04-197.05
F: FTMS - p ESI Full ms [70.0000-1050.0000] MS Acai_EtOH_300
RT: 4.50
MA: 77062477
Relative Abundance
Time (min)
Acai_EtOH_300 #538 RT: 4.53 AV: 1 NL: 2.77E8
T: FTMS - p ESI Full ms [70.0000-1050.0000]
181.05090
Relative Abundance
m/z

Vanillic acid

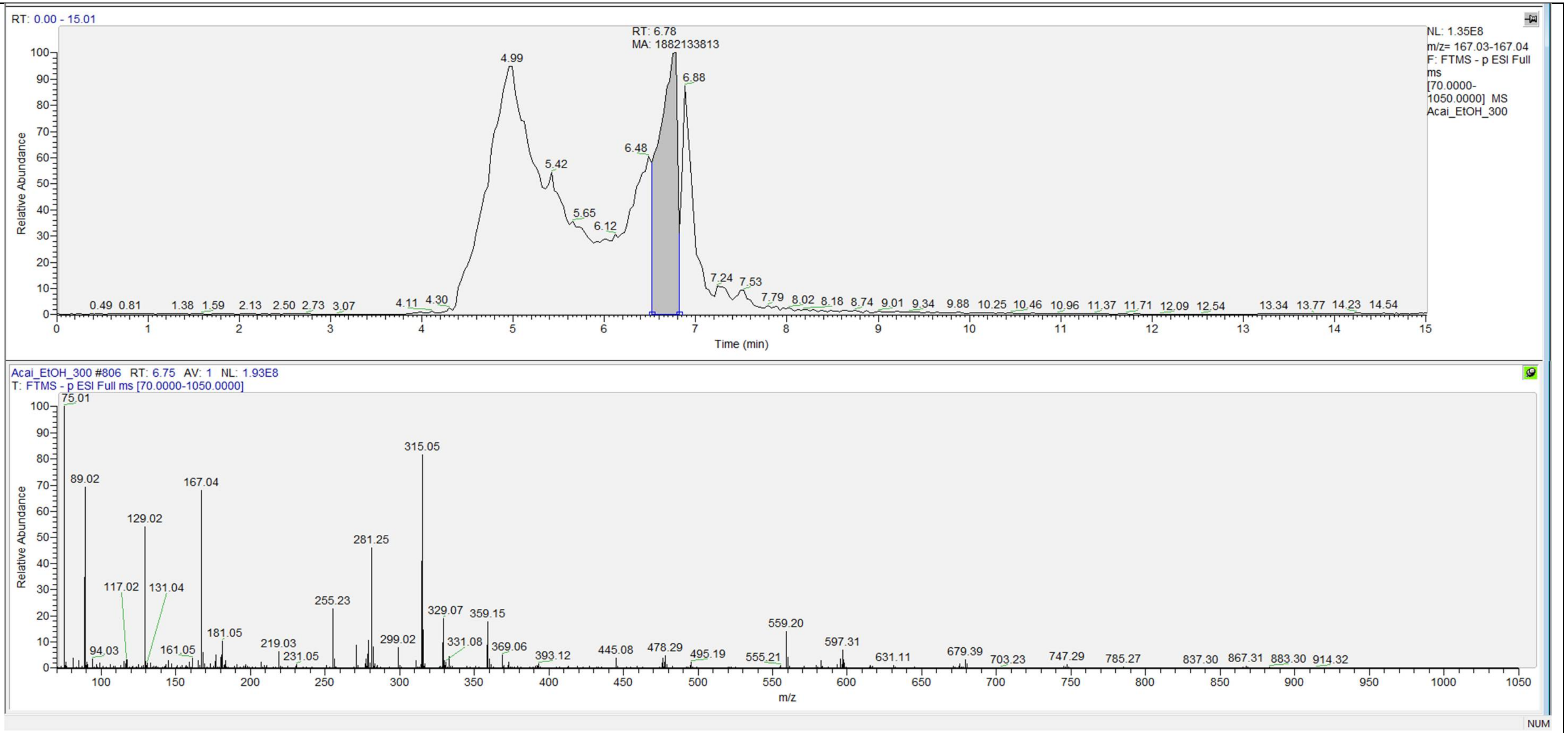

	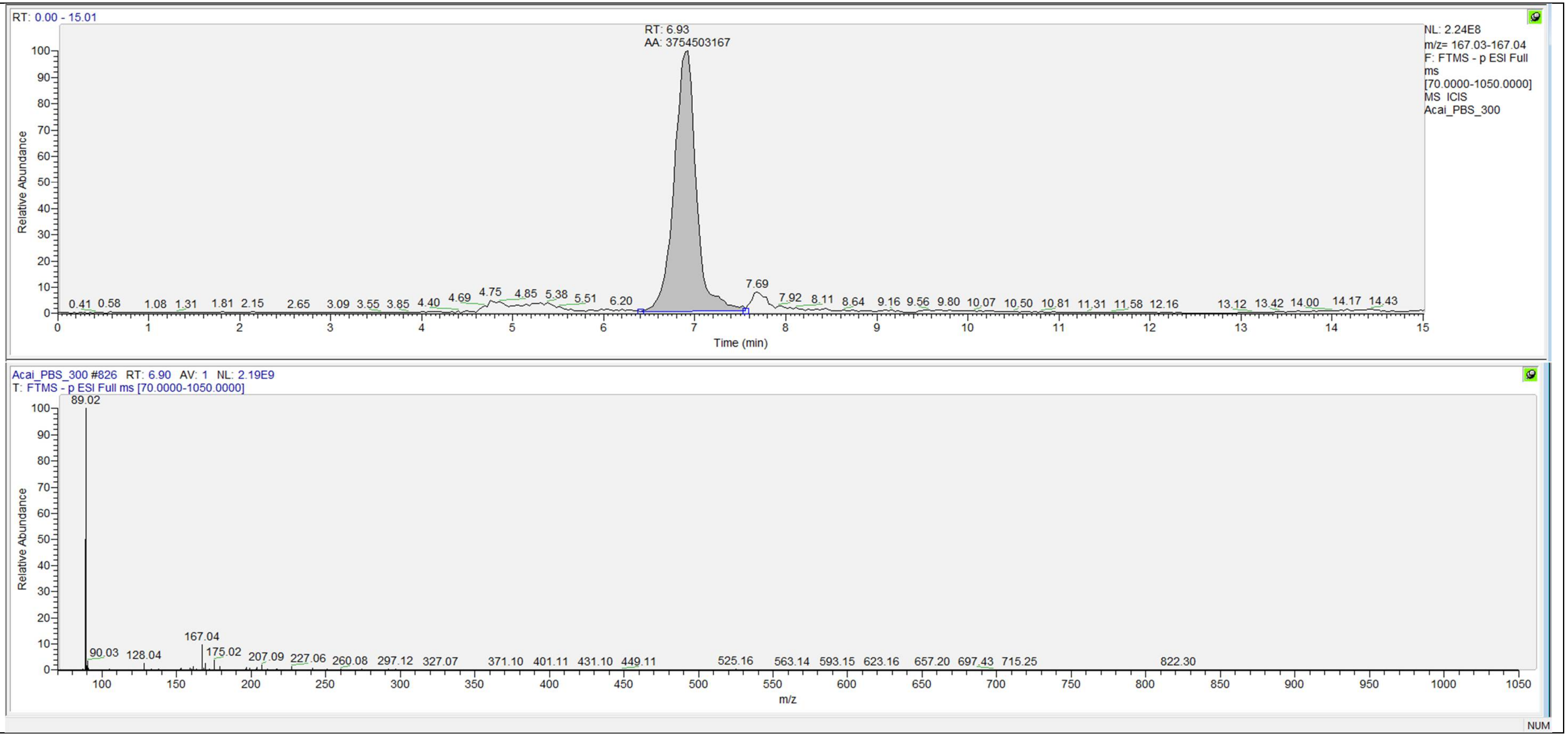RT: 0.00 - 15.01
RT: 6.93
AA: 3754503167
NL: 2.24E8
m/z= 167.03-167.04
F: FTMS - p ESI Full ms [70.0000-1050.0000]
MS ICIS
Acai_PBS_300
Relative Abundance
Time (min)
7.69
Acai_PBS_300 #826 RT: 6.90 AV: 1 NL: 2.19E9
T: FTMS - p ESI Full ms [70.0000-1050.0000]
89.02
167.04
m/z

Gallic acid	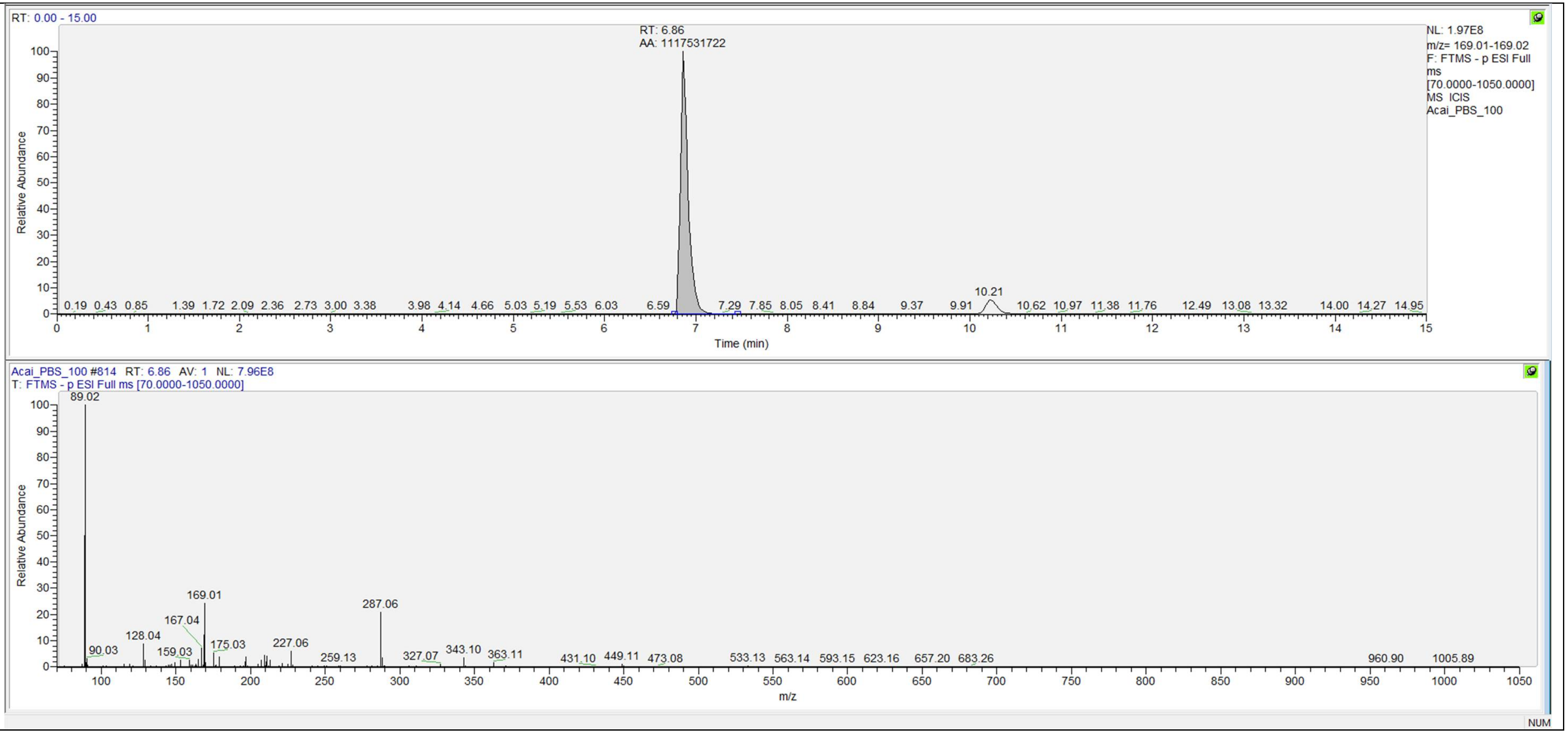

RT: 0.00 - 15.00
RT: 6.86
AA: 1117531722
NL: 1.97E8
m/z= 169.01-169.02
F: FTMS - p ESI Full ms [70.0000-1050.0000]
MS ICIS
Acai_PBS_100
Relative Abundance
10.21
Time (min)
Acai_PBS_100 #814 RT: 6.86 AV: 1 NL: 7.96E8
T: FTMS - p ESI Full ms [70.0000-1050.0000]
89.02
169.01
287.06
167.04
128.04
175.03
227.06
159.03
90.03
259.13
327.07
343.10
363.11
431.10
449.11
473.08
533.13
563.14
593.15
623.16
657.20
683.26
960.90
1005.89
m/z

4-Hydroxybenzoic acid	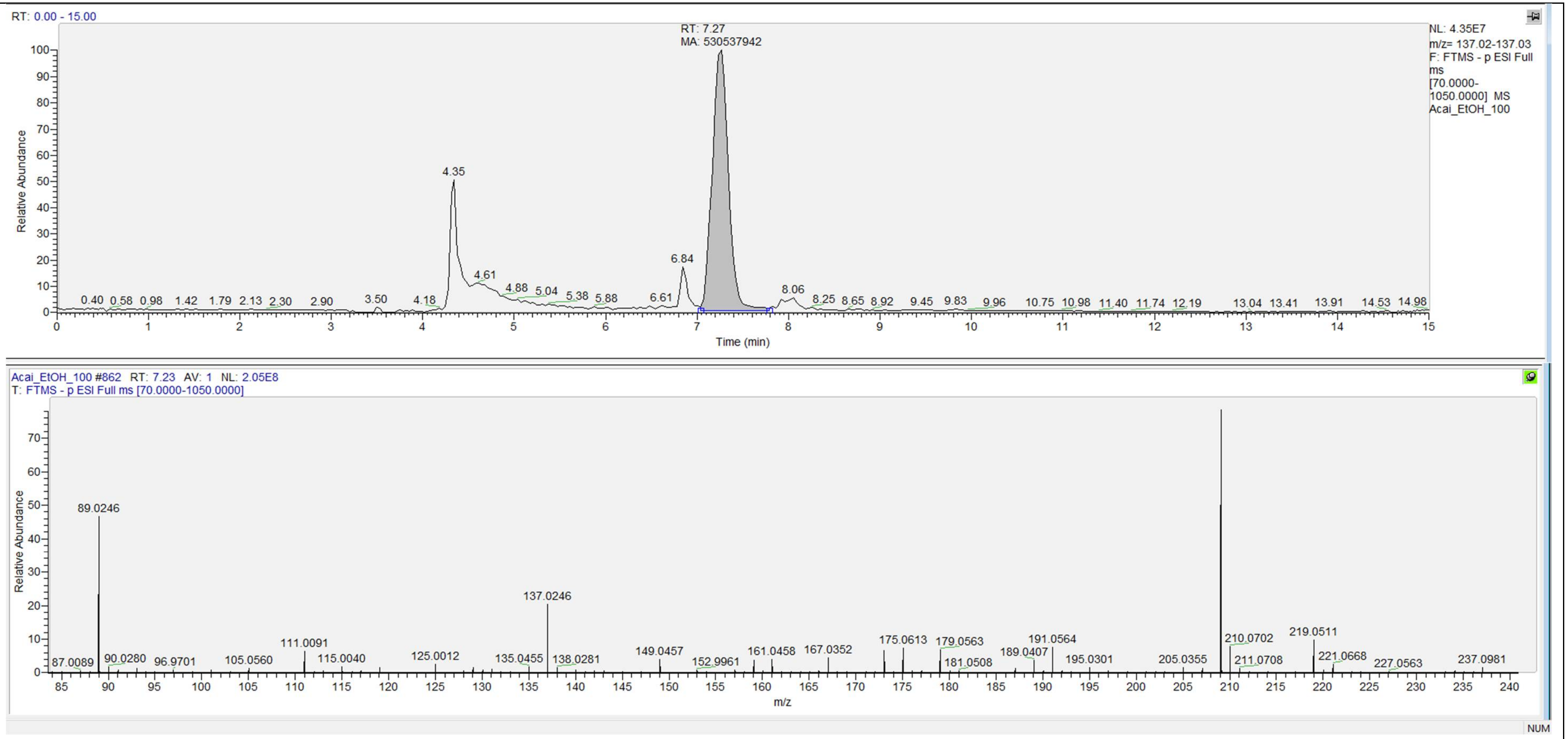

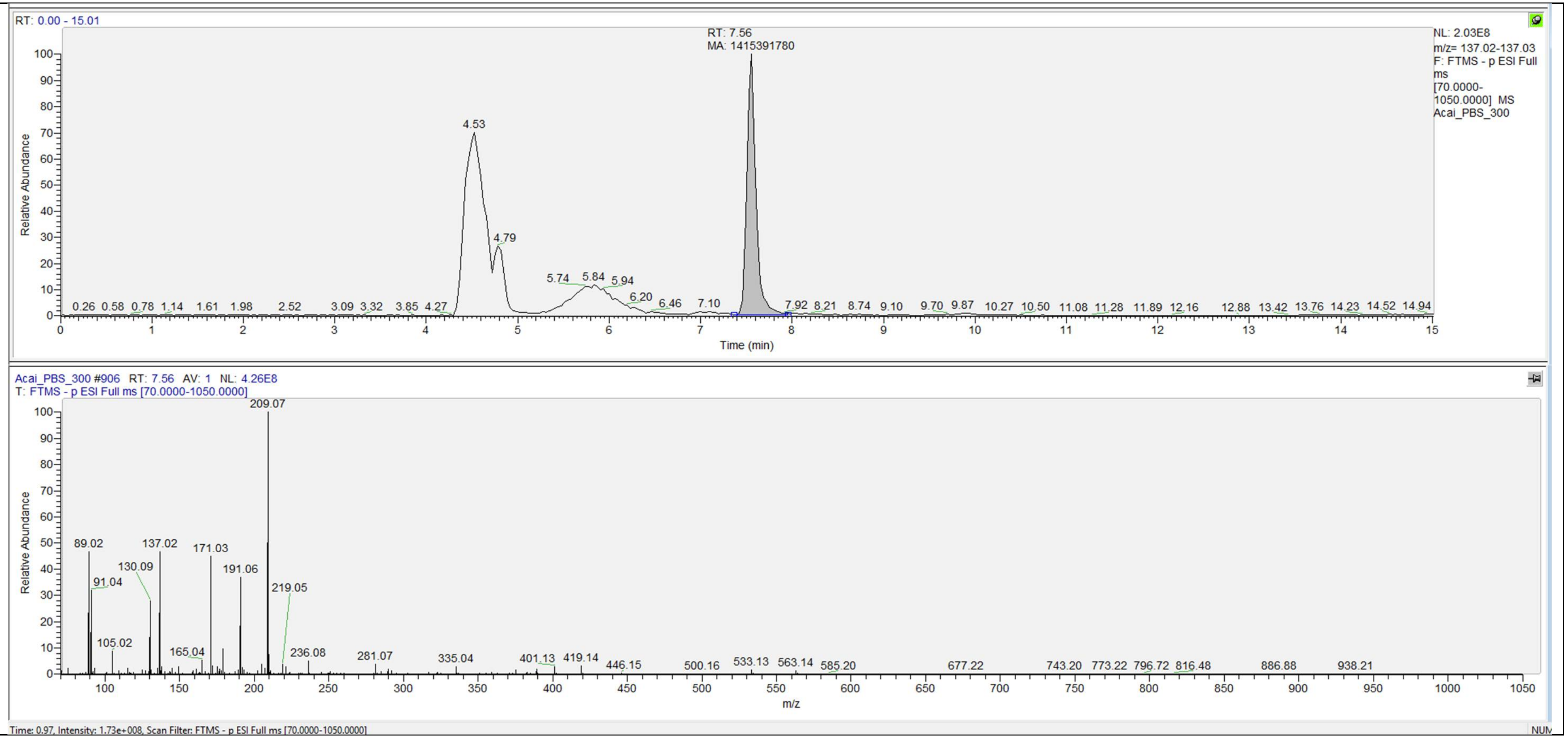

RT: 0.00 - 15.01
RT: 7.56
MA: 1415391780
NL: 2.03E8
m/z= 137.02-137.03
F: FTMS - p ESI Full ms [70.0000-1050.0000] MS Acai_PBS_300
Relative Abundance
Time (min)
4.53
4.79
5.74 5.84 5.94
6.20
6.46
7.10
7.92 8.21 8.74 9.10 9.70 9.87 10.27 10.50 11.08 11.28 11.89 12.16 12.88 13.42 13.76 14.23 14.52 14.94
0.26 0.58 0.78 1.14 1.61 1.98 2.52 3.09 3.32 3.85 4.27
Acai_PBS_300 #906 RT: 7.56 AV: 1 NL: 4.26E8
T: FTMS - p ESI Full ms [70.0000-1050.0000]
209.07
89.02
137.02
171.03
130.09
191.06
91.04
219.05
105.02
165.04
236.08
281.07
335.04
401.13 419.14
446.15
500.16
533.13 563.14
585.20
677.22
743.20 773.22 796.72 816.48
886.88
938.21
m/z
Time: 0.97, Intensity: 1.73e+008, Scan Filter: FTMS - p ESI Full ms [70.0000-1050.0000]

Benzoic acid	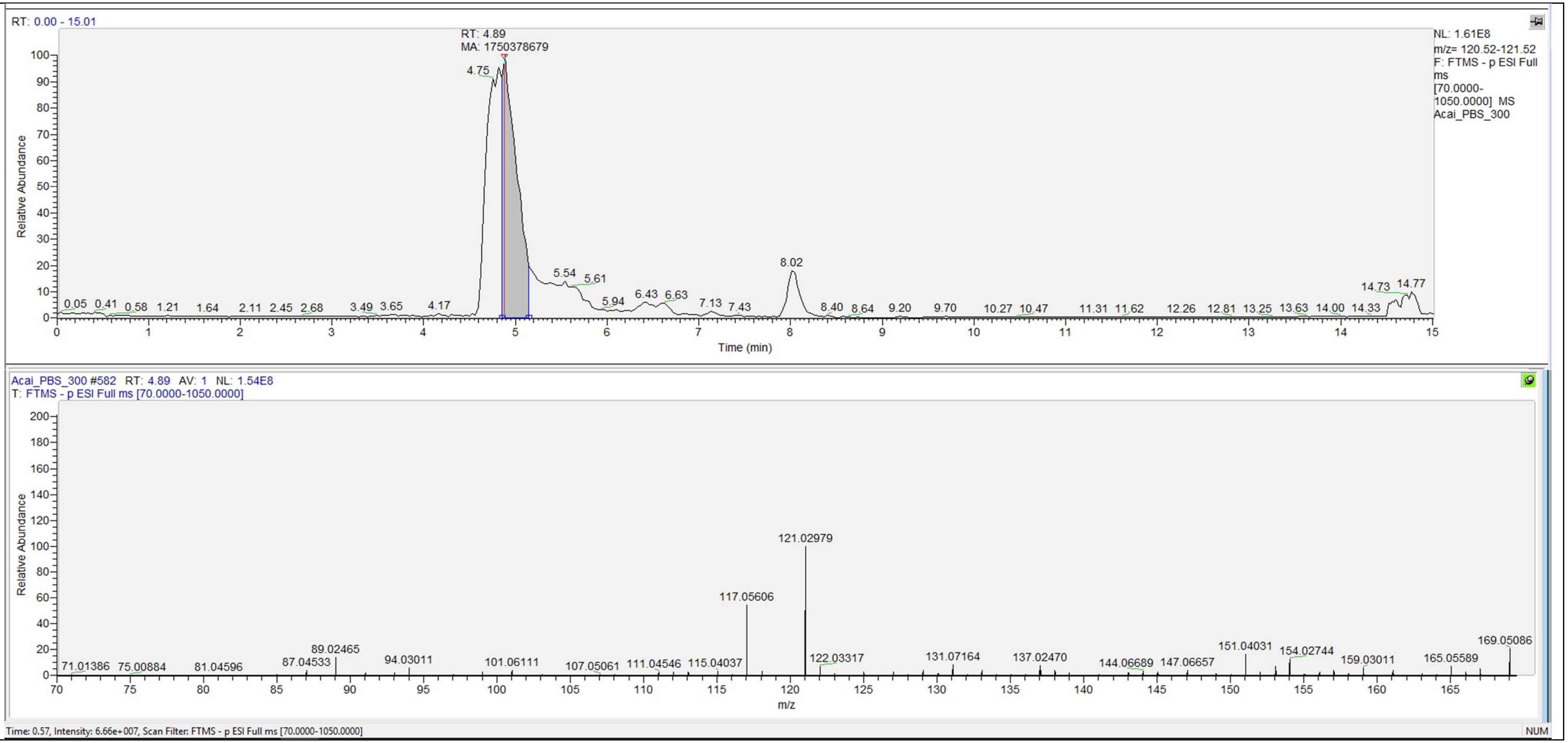

2,5-Dihydroxybenzoic acid	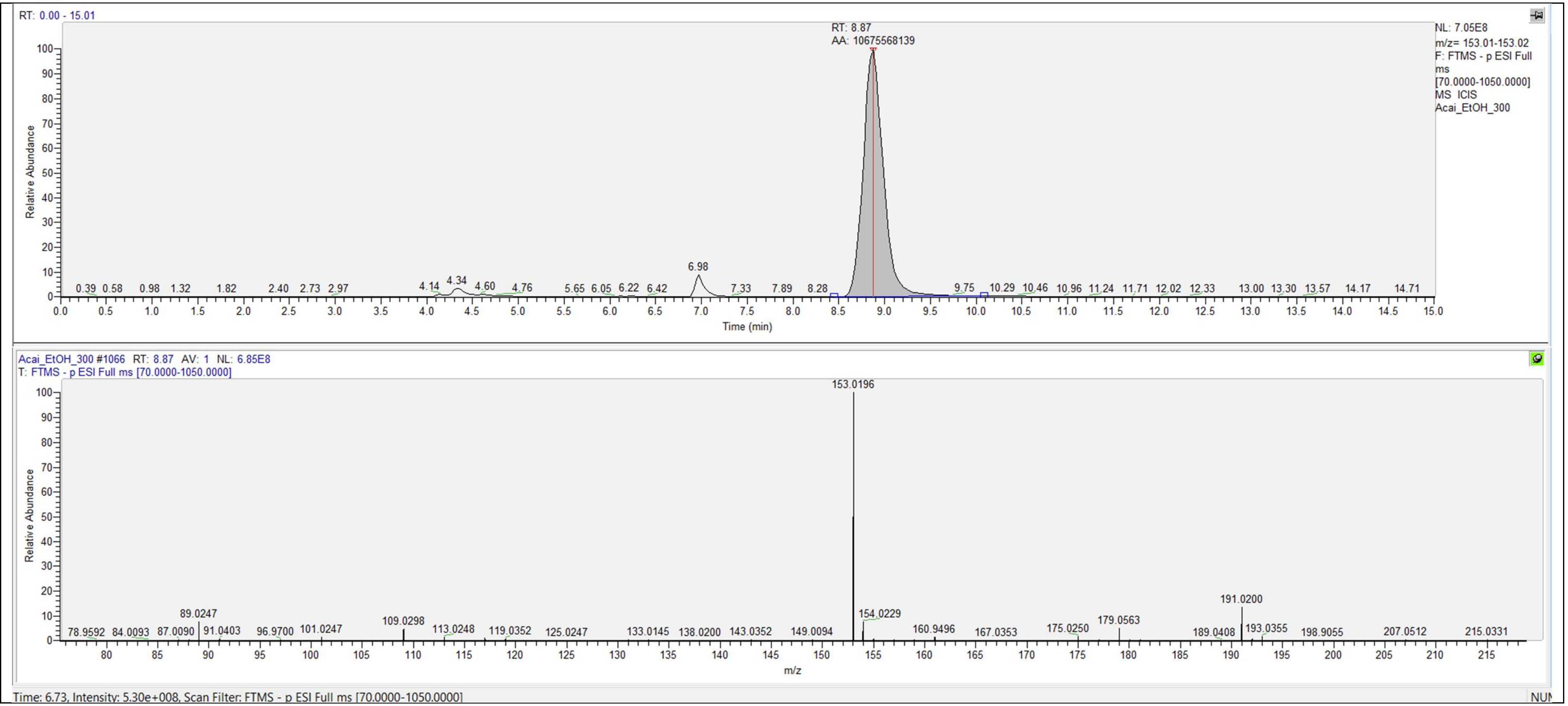

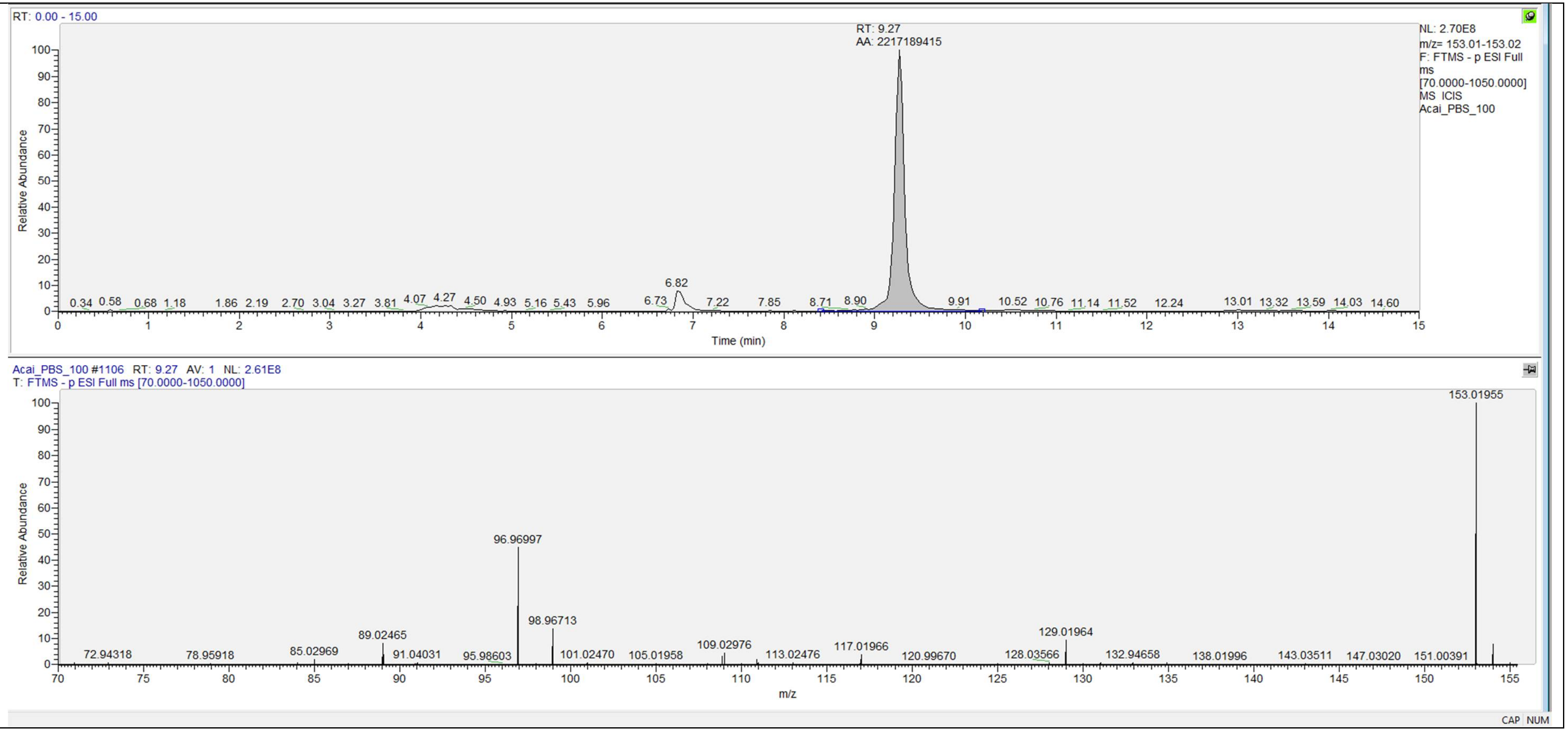

RT: 0.00 - 15.00
RT: 9.27
AA: 2217189415
NL: 2.70E8
m/z= 153.01-153.02
F: FTMS - p ESI Full ms [70.0000-1050.0000]
MS ICIS
Acai_PBS_100
Relative Abundance
Time (min)
6.82
Acai_PBS_100 #1106 RT: 9.27 AV: 1 NL: 2.61E8
T: FTMS - p ESI Full ms [70.0000-1050.0000]
153.01955
96.96997
98.96713
89.02465
129.01964
109.02976
117.01966
m/z

Chlorogenic acid

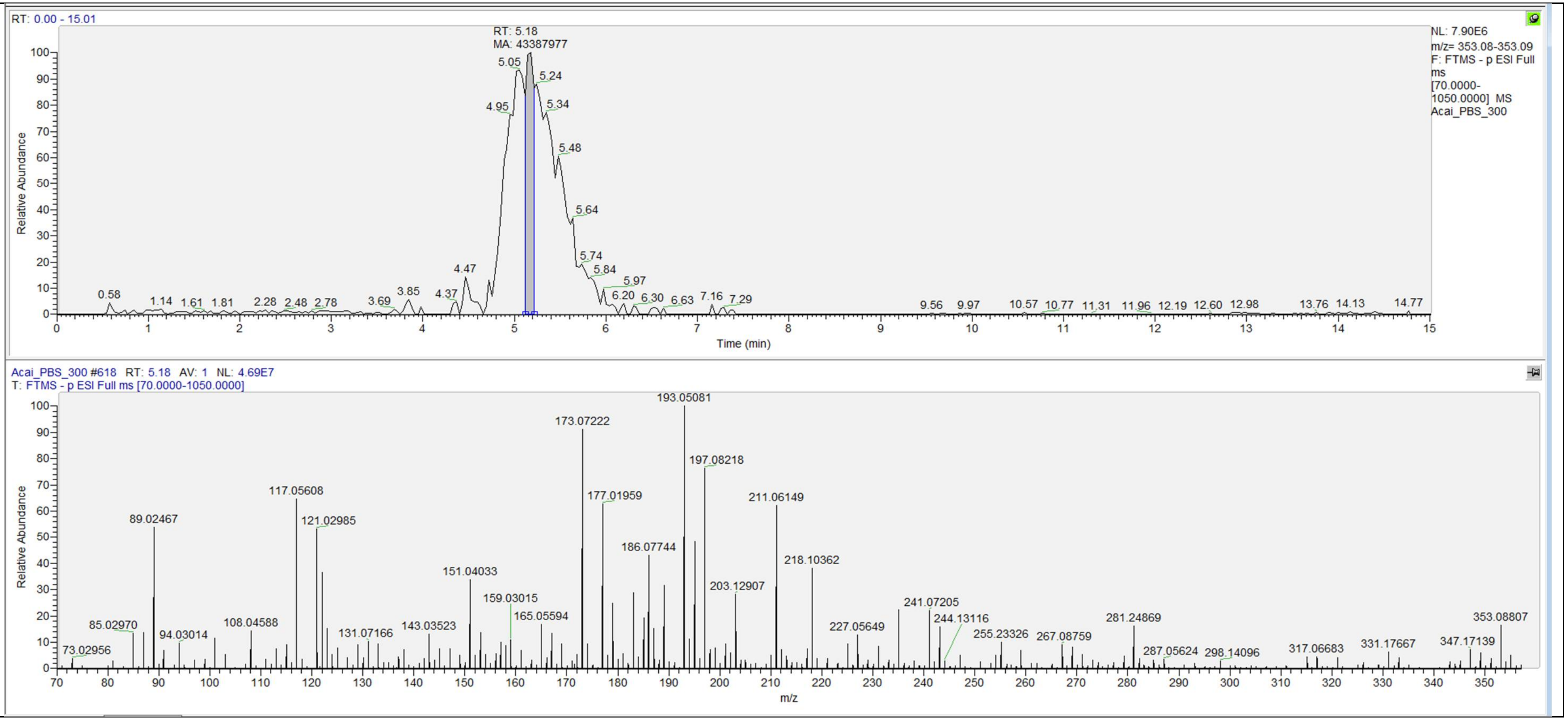

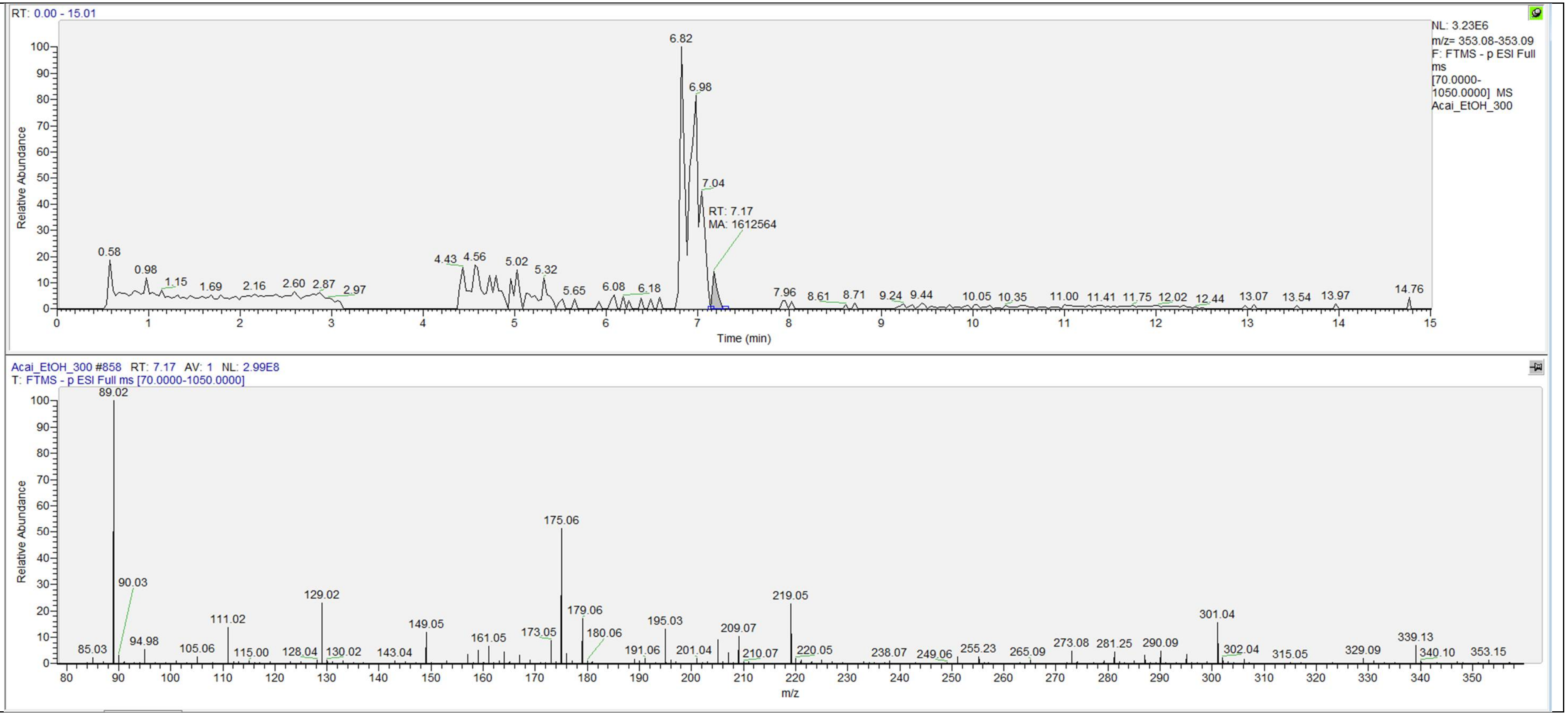
RT: 0.00 - 15.01
NL: 3.23E6
m/z= 353.08-353.09
F: FTMS - p ESI Full ms [70.0000-1050.0000] MS
Acai_EtOH_300
Relative Abundance
Time (min)
6.82
6.98
7.04
RT: 7.17
MA: 1612564
0.58
0.98
1.15
1.69
2.16
2.60
2.87
2.97
4.43
4.56
5.02
5.32
5.65
6.08
6.18
7.96
8.61
8.71
9.24
9.44
10.05
10.35
11.00
11.41
11.75
12.02
12.44
13.07
13.54
13.97
14.76
Acai_EtOH_300 #858 RT: 7.17 AV: 1 NL: 2.99E8
T: FTMS - p ESI Full ms [70.0000-1050.0000]
m/z
89.02
90.03
85.03
94.98
105.06
111.02
115.00
128.04
129.02
130.02
143.04
149.05
161.05
173.05
175.06
179.06
180.06
191.06
195.03
201.04
209.07
210.07
219.05
220.05
238.07
249.06
255.23
265.09
273.08
281.25
290.09
301.04
302.04
315.05
329.09
339.13
340.10
353.15

Dihydrokaempferol	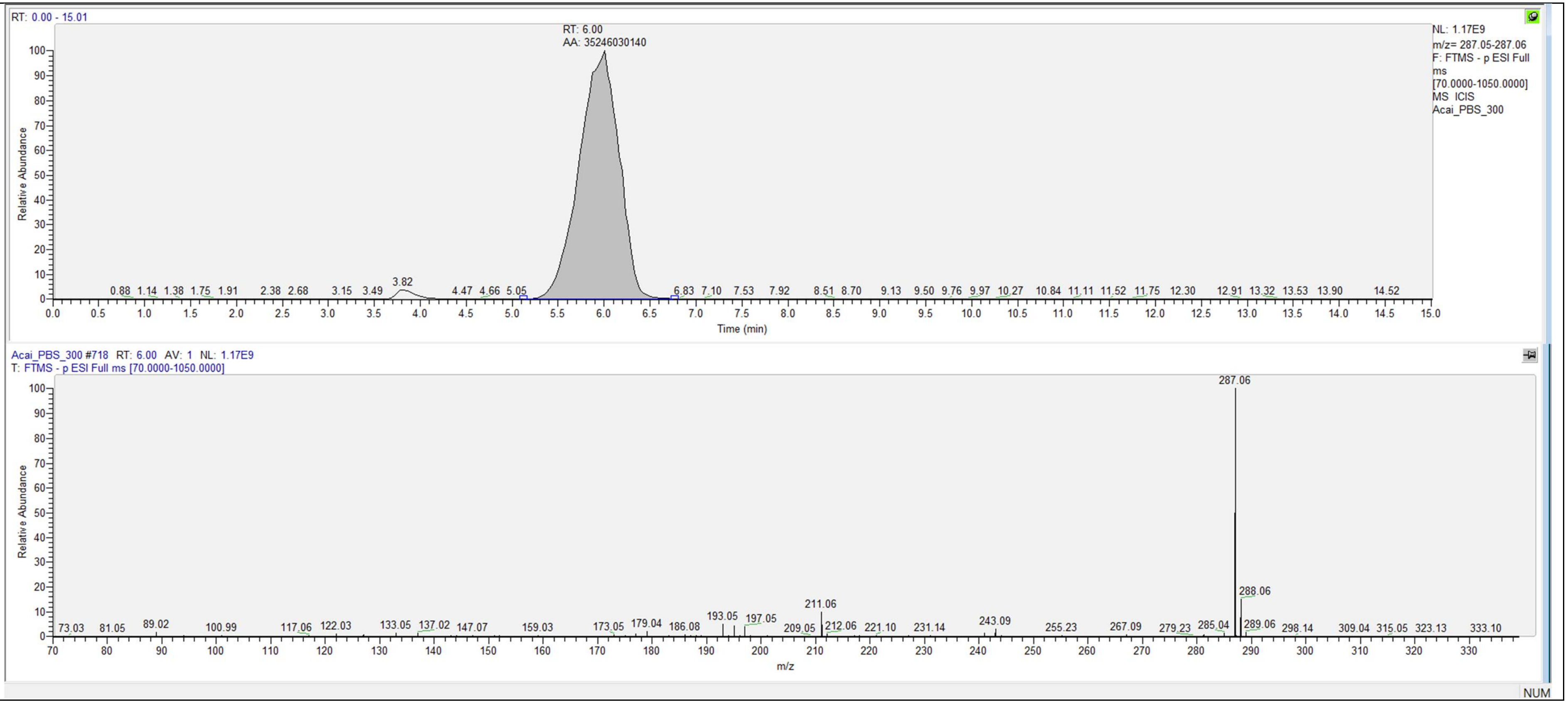

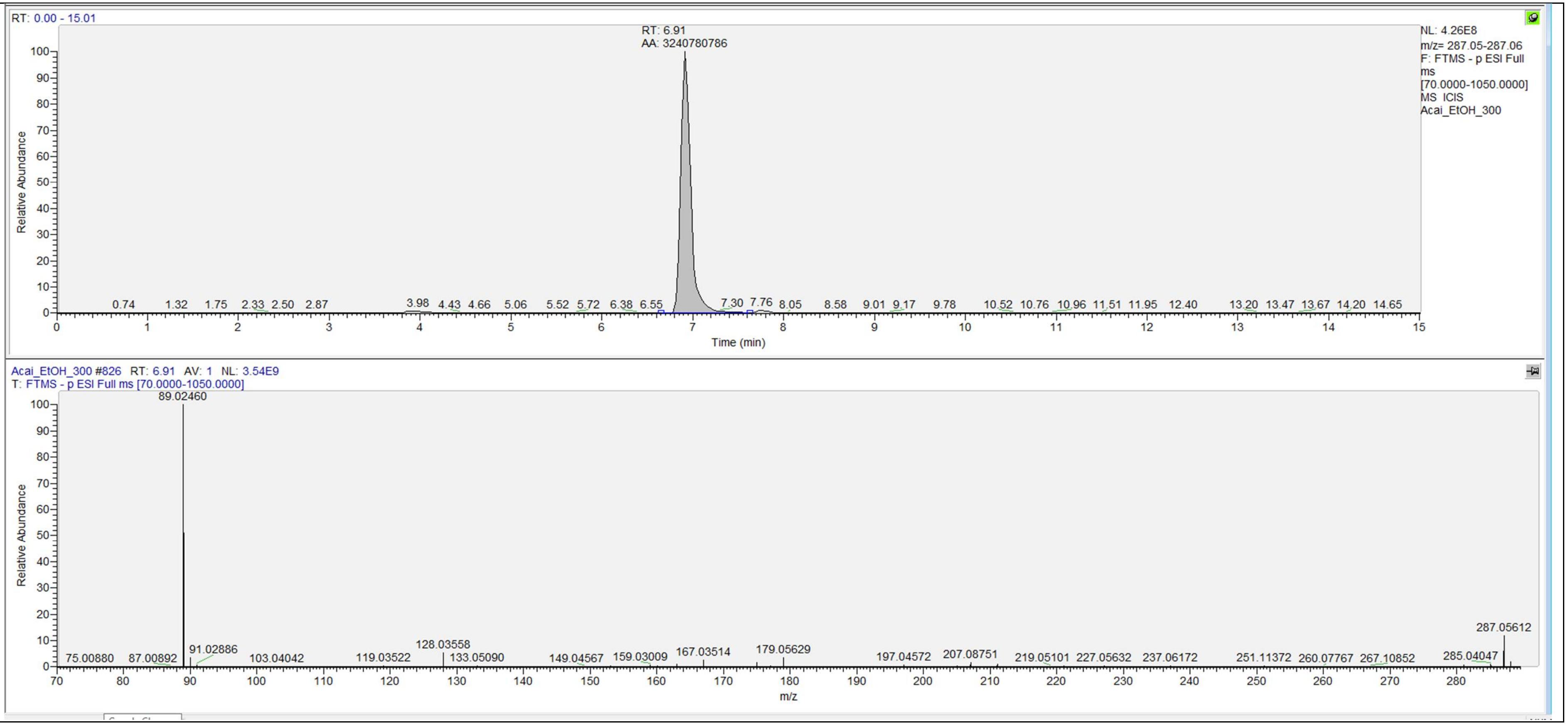
RT: 0.00 - 15.01
RT: 6.91
AA: 3240780786
NL: 4.26E8
m/z= 287.05-287.06
F: FTMS - p ESI Full ms [70.0000-1050.0000] MS ICIS Acai_EtOH_300
Relative Abundance
Time (min)
Acai_EtOH_300 #826 RT: 6.91 AV: 1 NL: 3.54E9
T: FTMS - p ESI Full ms [70.0000-1050.0000]
89.02460
287.05612
128.03558
179.05629
167.03514
m/z

Luteolin	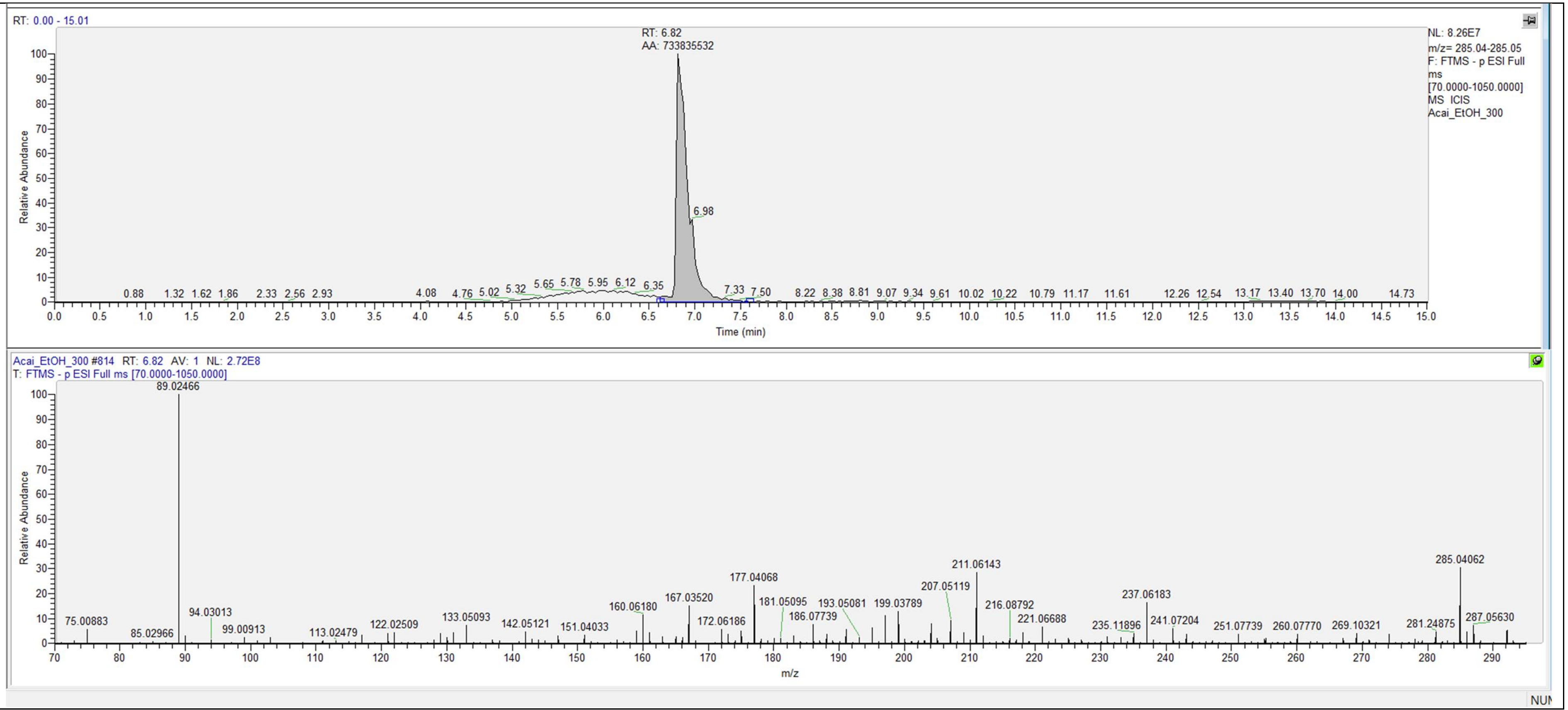

RT: 0.00 - 15.01
RT: 6.82
AA: 733835532
NL: 8.26E7
m/z= 285.04-285.05
F: FTMS - p ESI Full ms [70.0000-1050.0000]
MS ICIS
Acai_EtOH_300
Relative Abundance
Time (min)
Acai_EtOH_300 #814 RT: 6.82 AV: 1 NL: 2.72E8
T: FTMS - p ESI Full ms [70.0000-1050.0000]
89.02466
285.04062
211.06143
177.04068
m/z

Quercetin	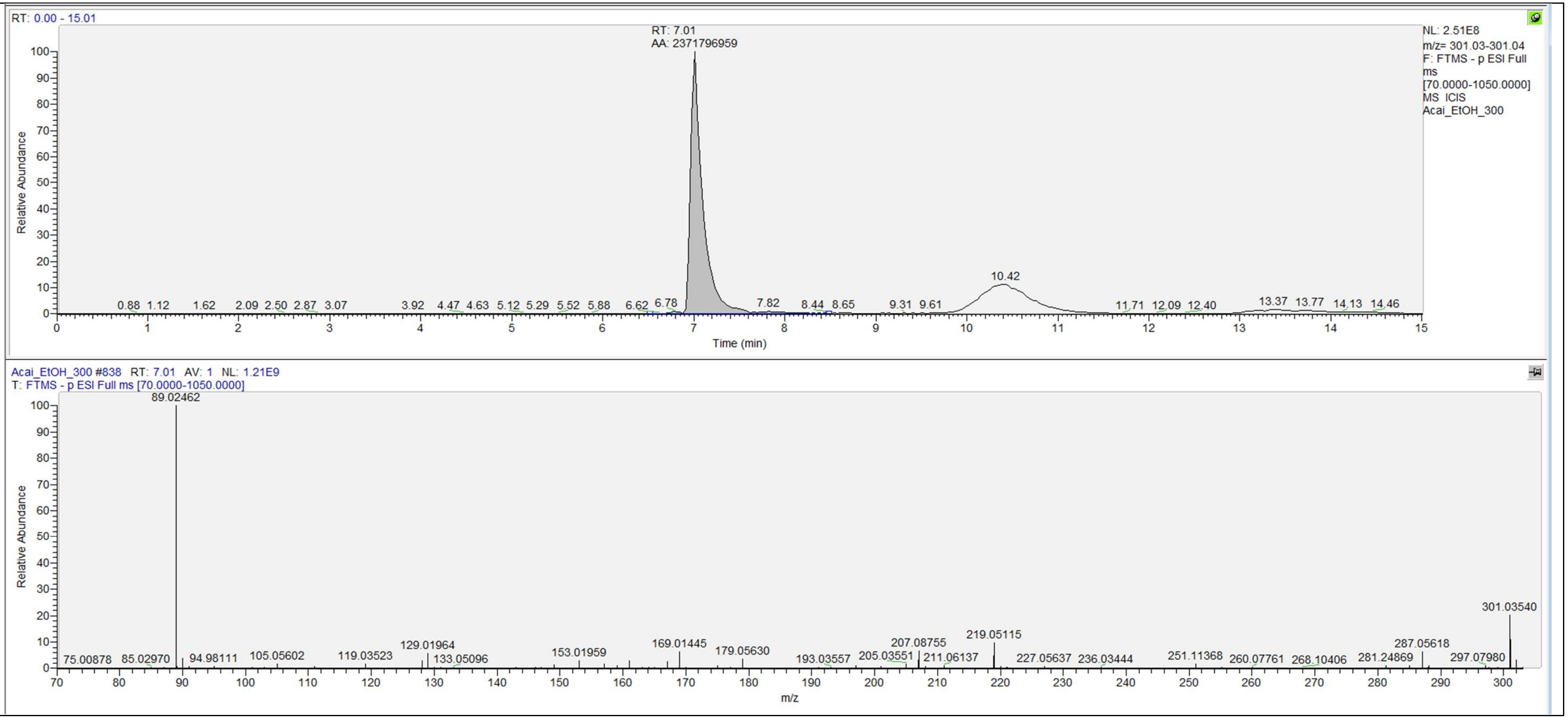

RT: 0.00 - 15.01
RT: 7.01
AA: 2371796959
NL: 2.51E8
m/z= 301.03-301.04
F: FTMS - p ESI Full ms [70.0000-1050.0000]
MS ICIS
Acai_EtOH_300
10.42
Relative Abundance
Time (min)
Acai_EtOH_300 #838 RT: 7.01 AV: 1 NL: 1.21E9
T: FTMS - p ESI Full ms [70.0000-1050.0000]
89.02462
301.03540
219.05115
207.08755
169.01445
287.05618
129.01964
153.01959
179.05630
m/z

Taxifolin deoxyhexose or Taxifolin	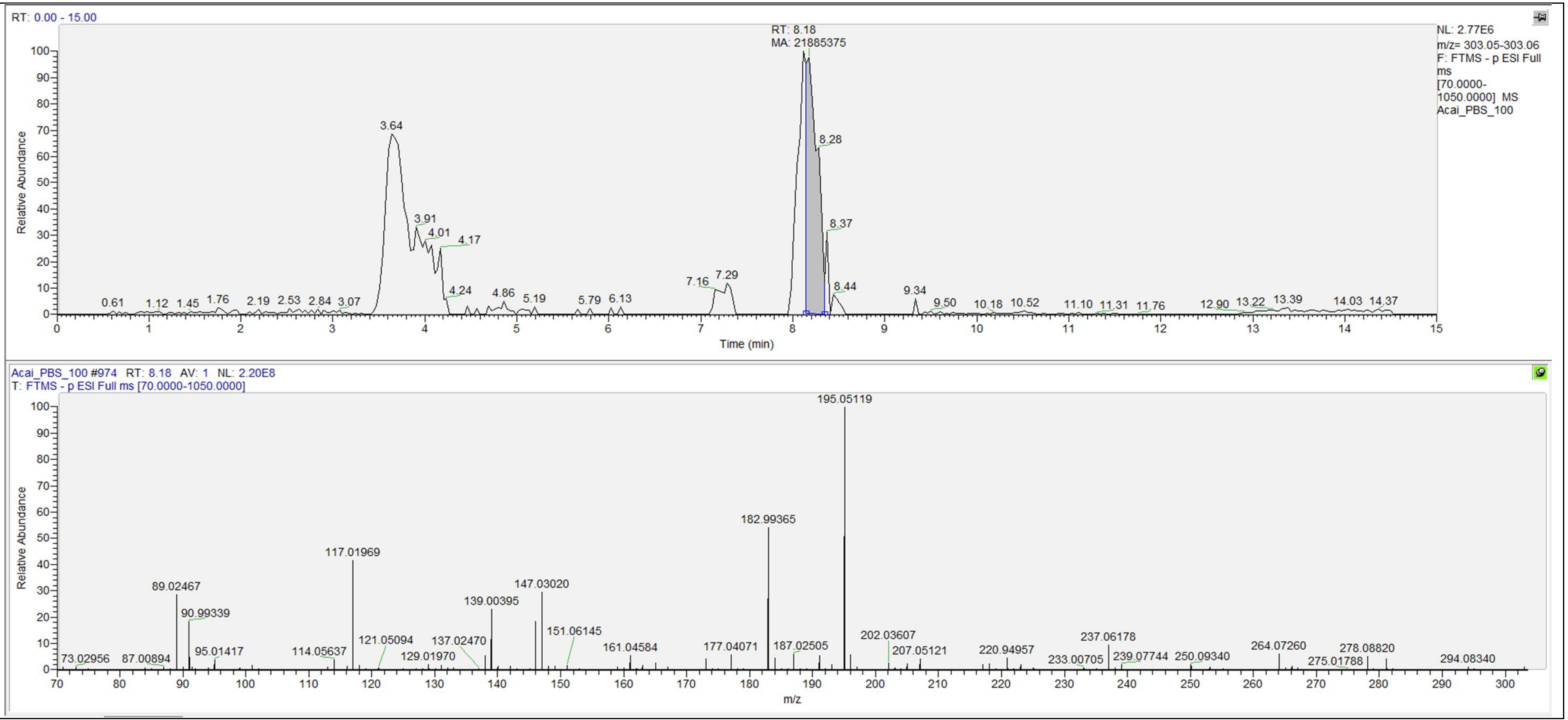

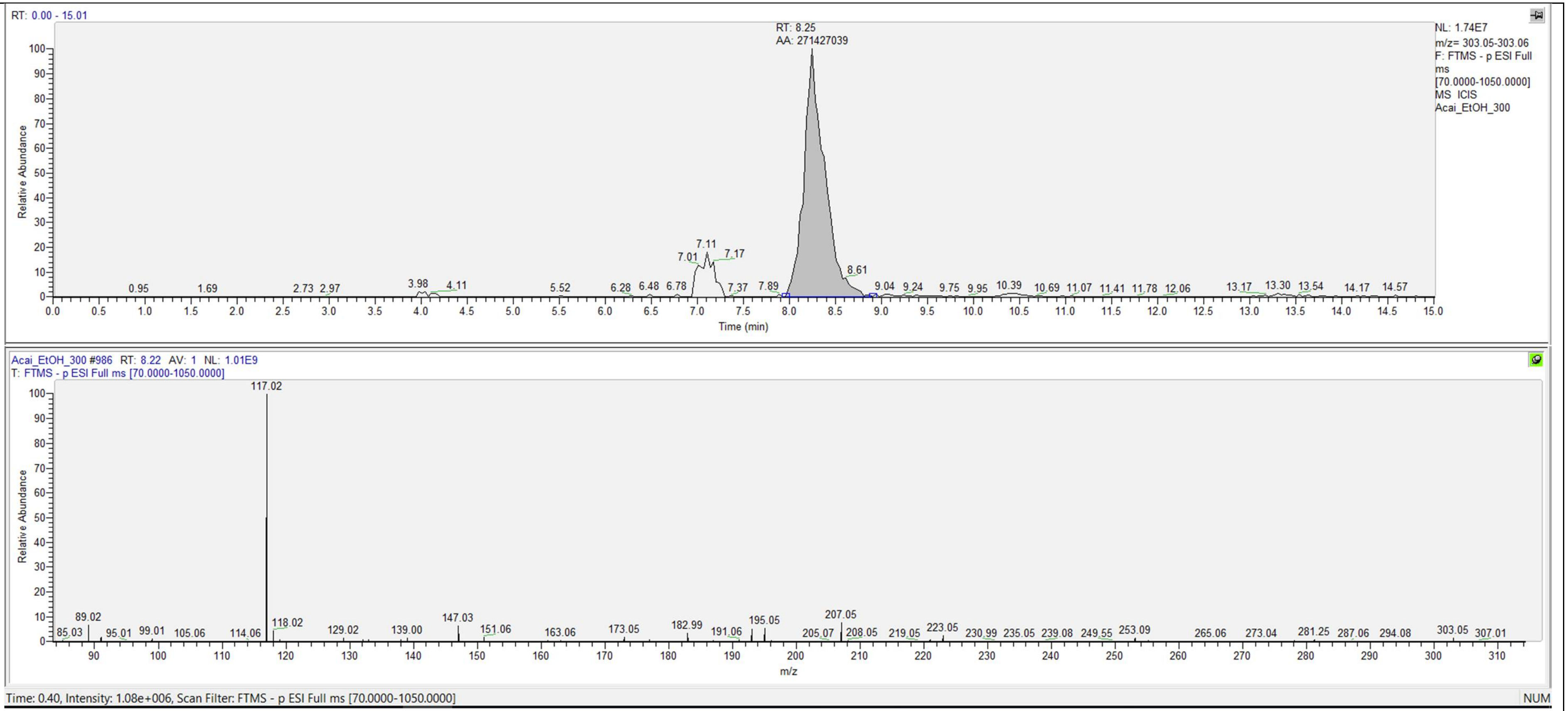
RT: 0.00 - 15.01
RT: 8.25
AA: 271427039
NL: 1.74E7
m/z= 303.05-303.06
F: FTMS - p ESI Full ms [70.0000-1050.0000]
MS ICIS
Acai_EtOH_300
Relative Abundance
Time (min)
Acai_EtOH_300 #986 RT: 8.22 AV: 1 NL: 1.01E9
T: FTMS - p ESI Full ms [70.0000-1050.0000]
117.02
Relative Abundance
m/z
Time: 0.40, Intensity: 1.08e+006, Scan Filter: FTMS - p ESI Full ms [70.0000-1050.0000]
NUM

Quercetin-3-O-rutinoside (rutin)	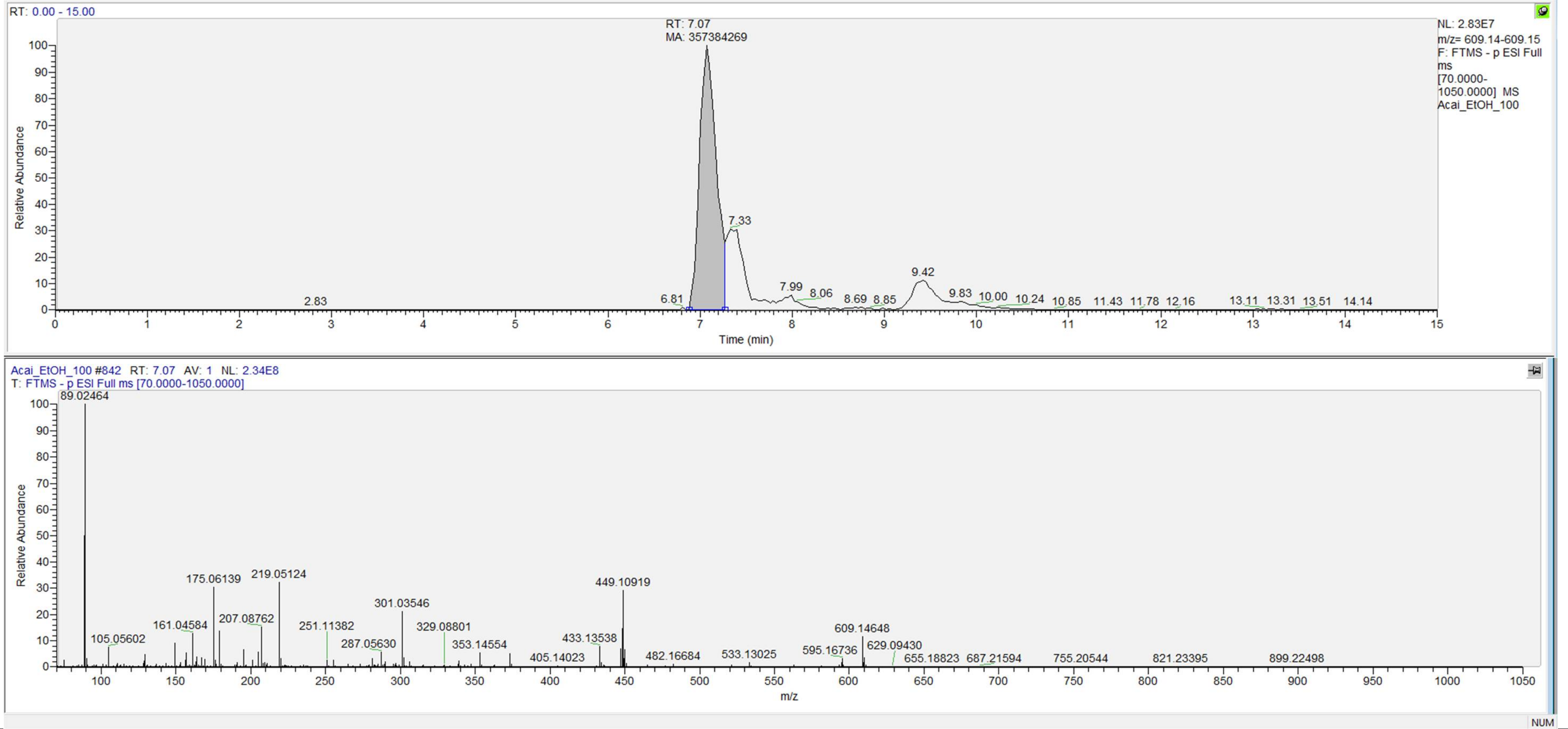

Quercetin 3-O-glucoside (Isoquercitrin)	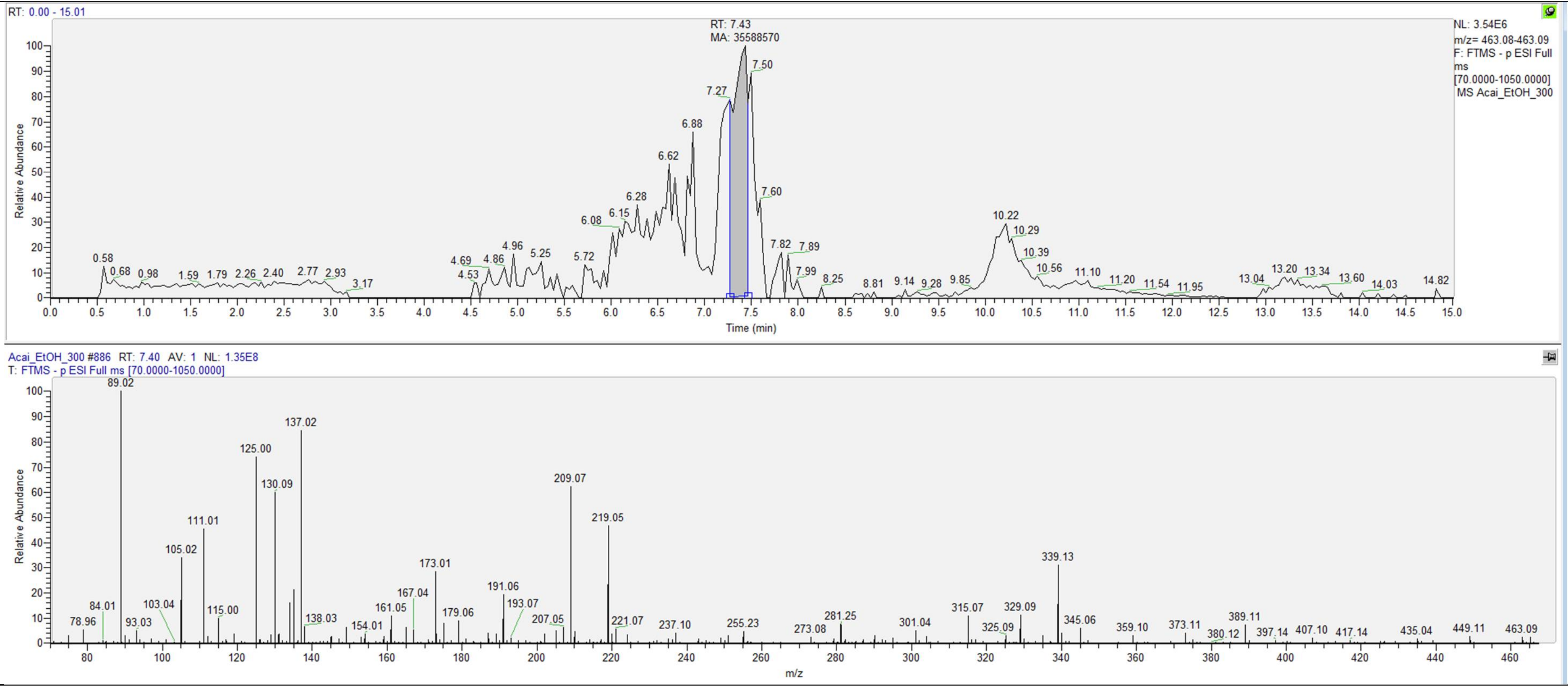

Kaempferol rhamnoside

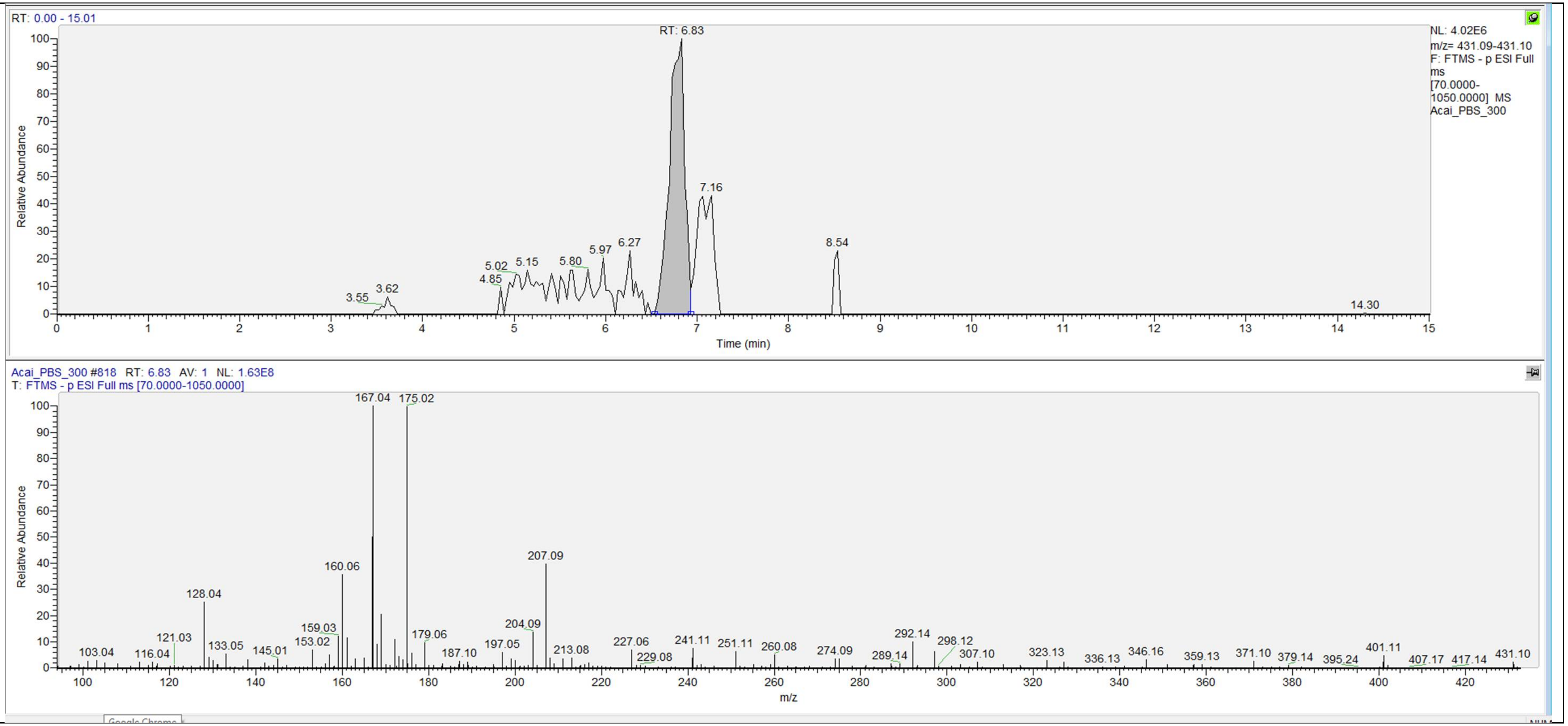

Isoorientin	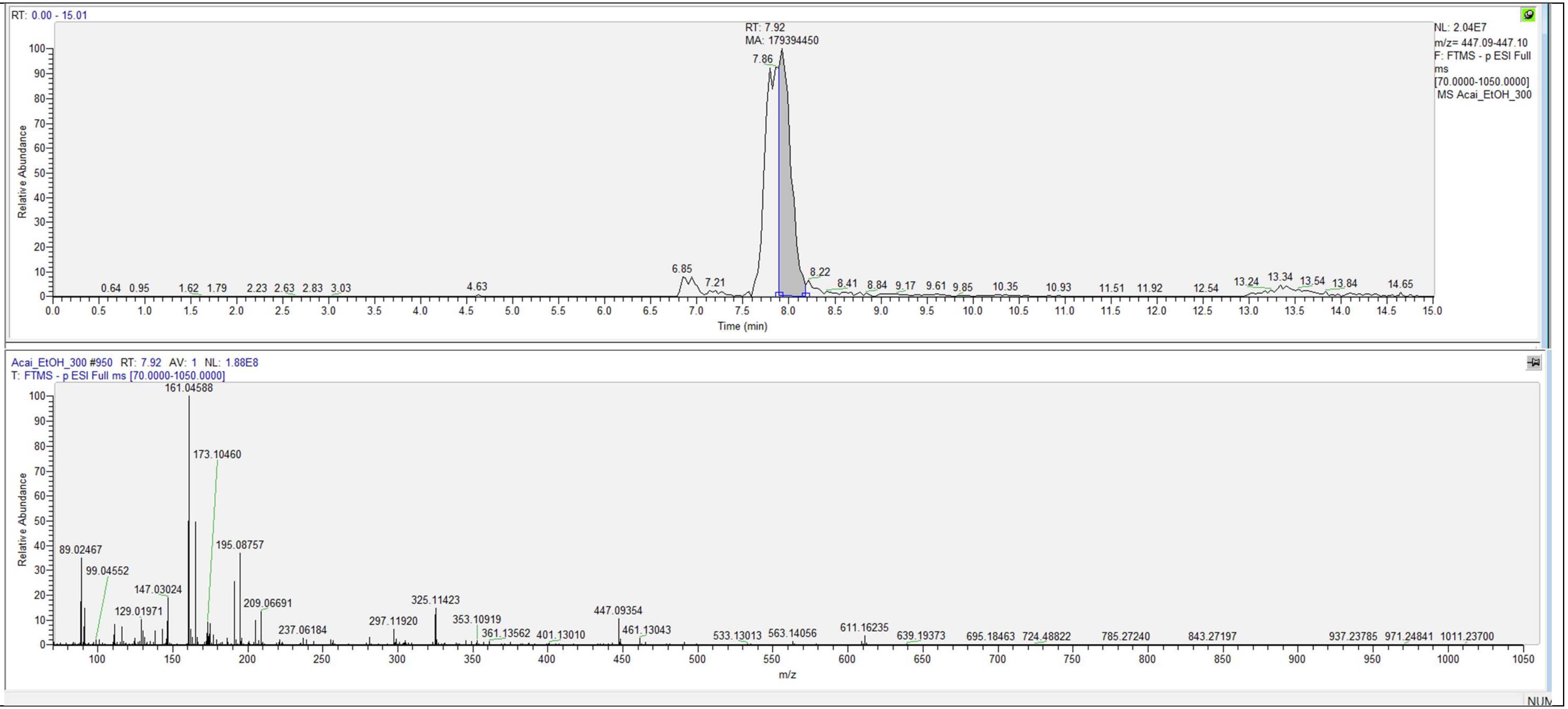

(+)-lariciresinol	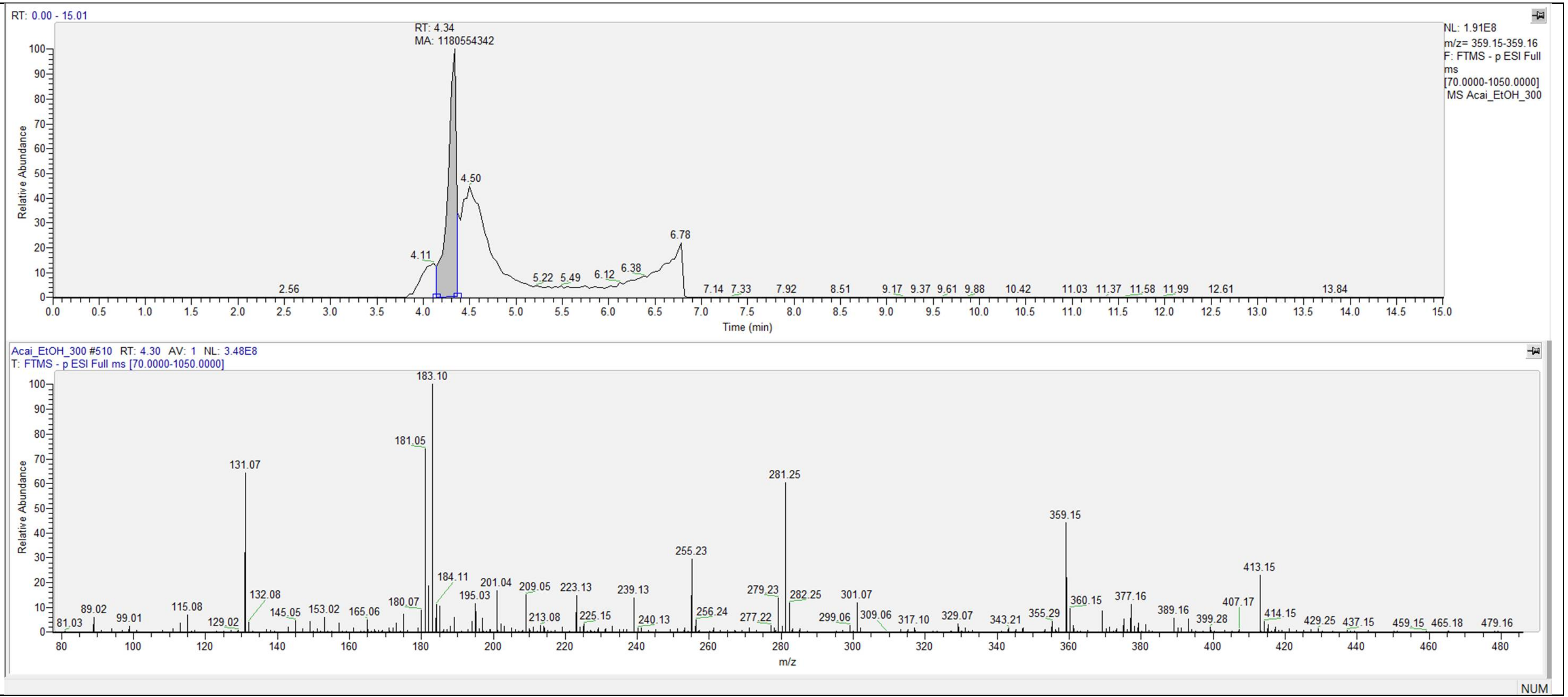

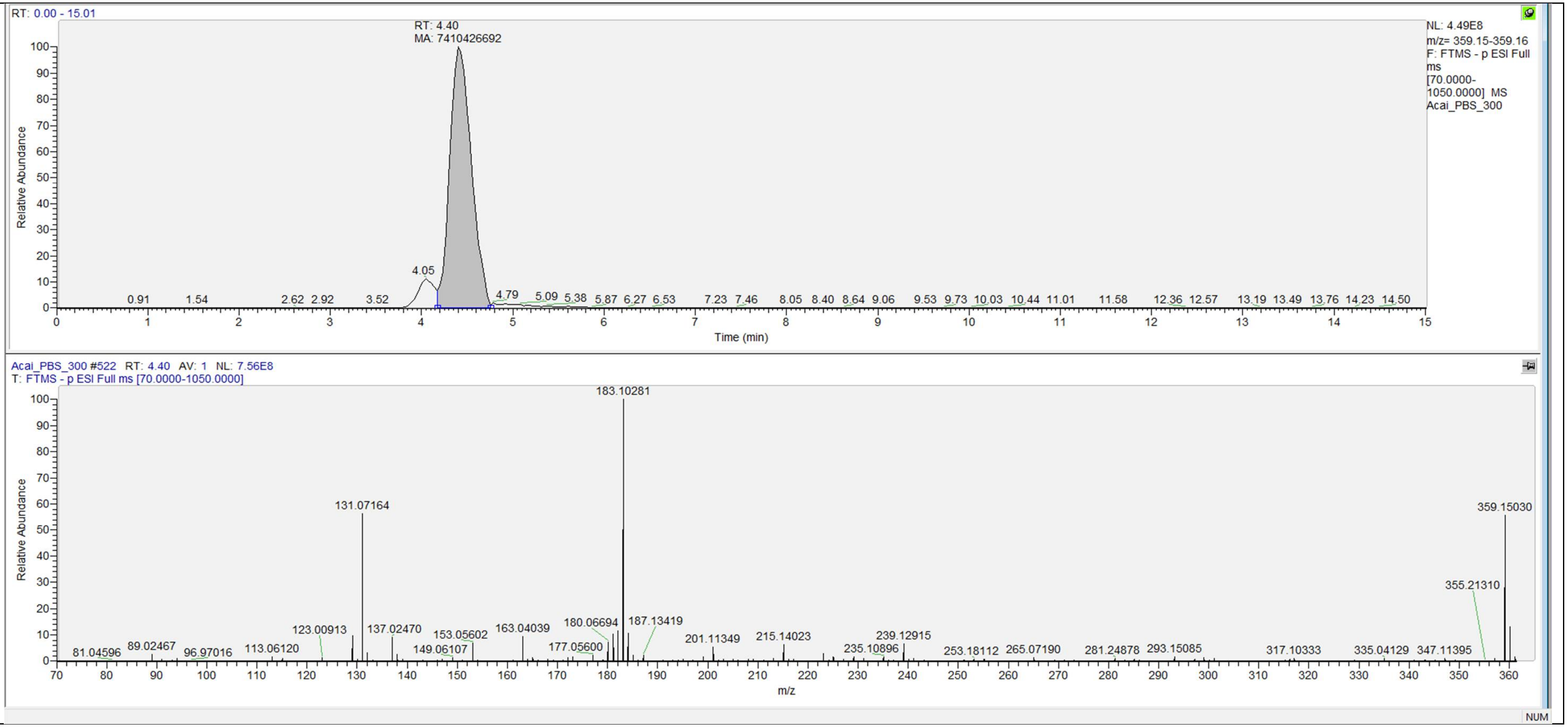
RT: 0.00 - 15.01
RT: 4.40
MA: 7410426692
NL: 4.49E8
m/z= 359.15-359.16
F: FTMS - p ESI Full ms [70.0000-1050.0000] MS Acai_PBS_300
Relative Abundance
Time (min)
Acai_PBS_300 #522 RT: 4.40 AV: 1 NL: 7.56E8
T: FTMS - p ESI Full ms [70.0000-1050.0000]
183.10281
131.07164
359.15030
355.21310
m/z

Dihydroconiferyl alcohol

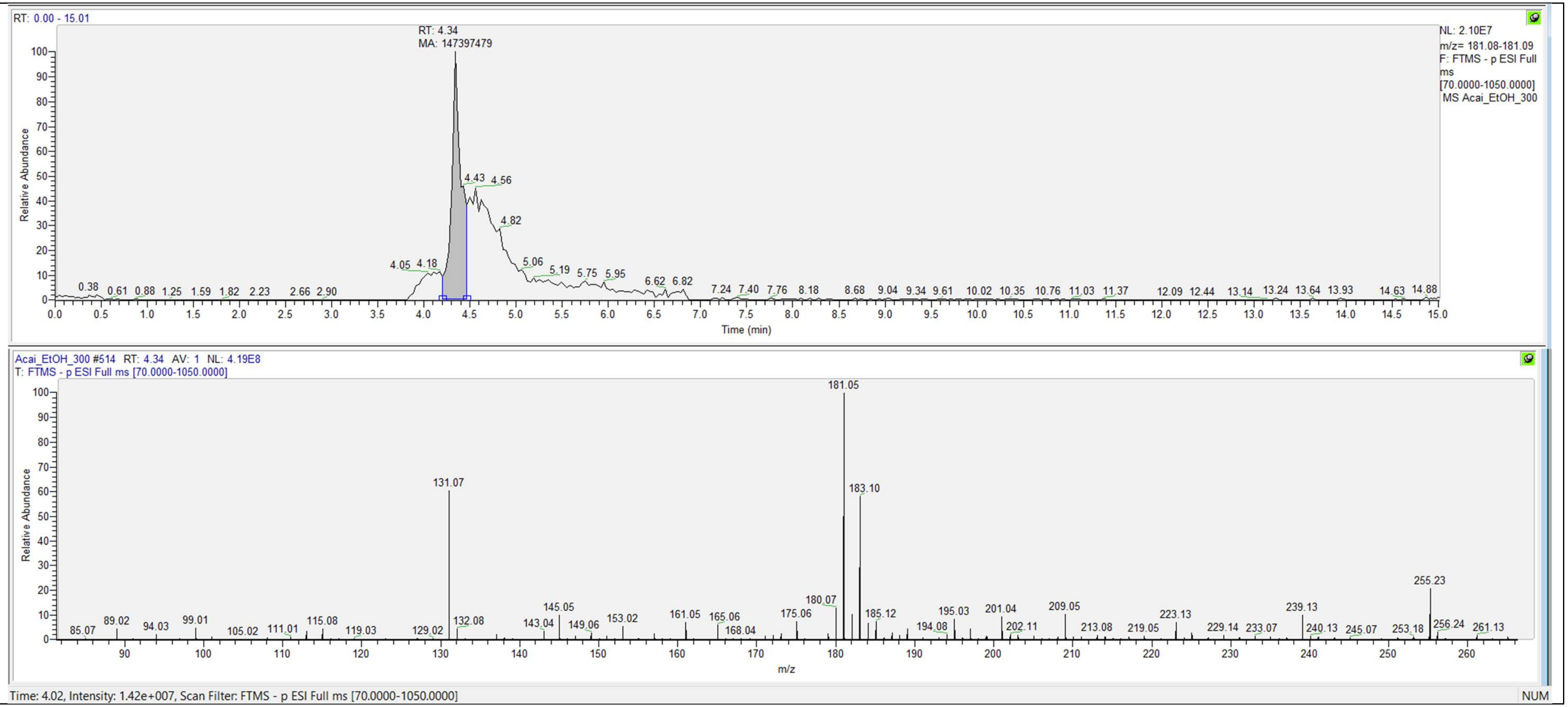

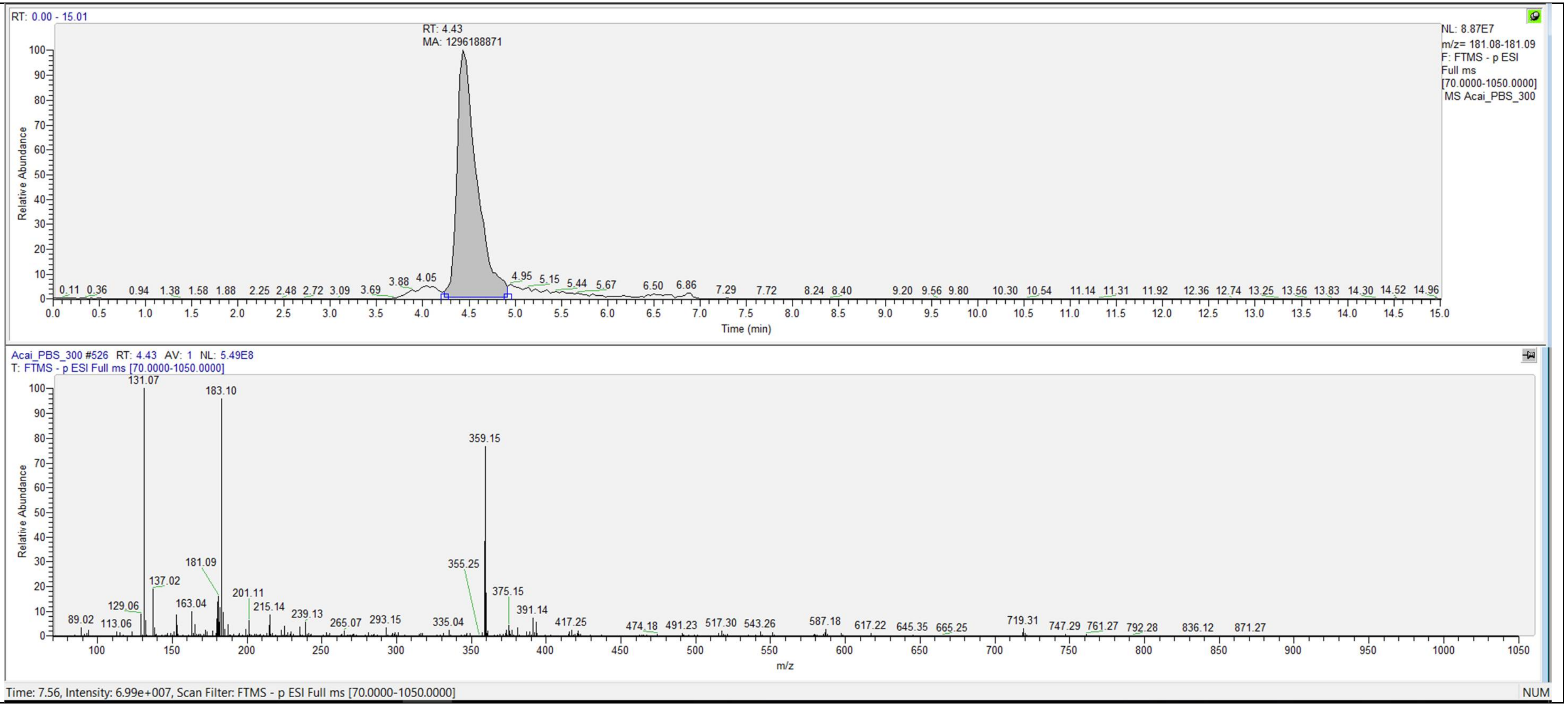

RT: 0.00 - 15.01
RT: 4.43
MA: 1296188871
NL: 8.87E7
m/z= 181.08-181.09
F: FTMS - p ESI
Full ms
[70.0000-1050.0000]
MS Acai_PBS_300
Relative Abundance
Time (min)
Acai_PBS_300 #526 RT: 4.43 AV: 1 NL: 5.49E8
T: FTMS - p ESI Full ms [70.0000-1050.0000]
131.07
183.10
359.15
181.09
355.25
375.15
137.02
201.11
129.06
163.04
215.14
391.14
239.13
89.02
113.06
265.07
293.15
335.04
417.25
474.18
491.23
517.30
543.26
587.18
617.22
645.35
665.25
719.31
747.29
761.27
792.28
836.12
871.27
Relative Abundance
m/z
Time: 7.56, Intensity: 6.99e+007, Scan Filter: FTMS - p ESI Full ms [70.0000-1050.0000]
NUM

(+)-Catechin	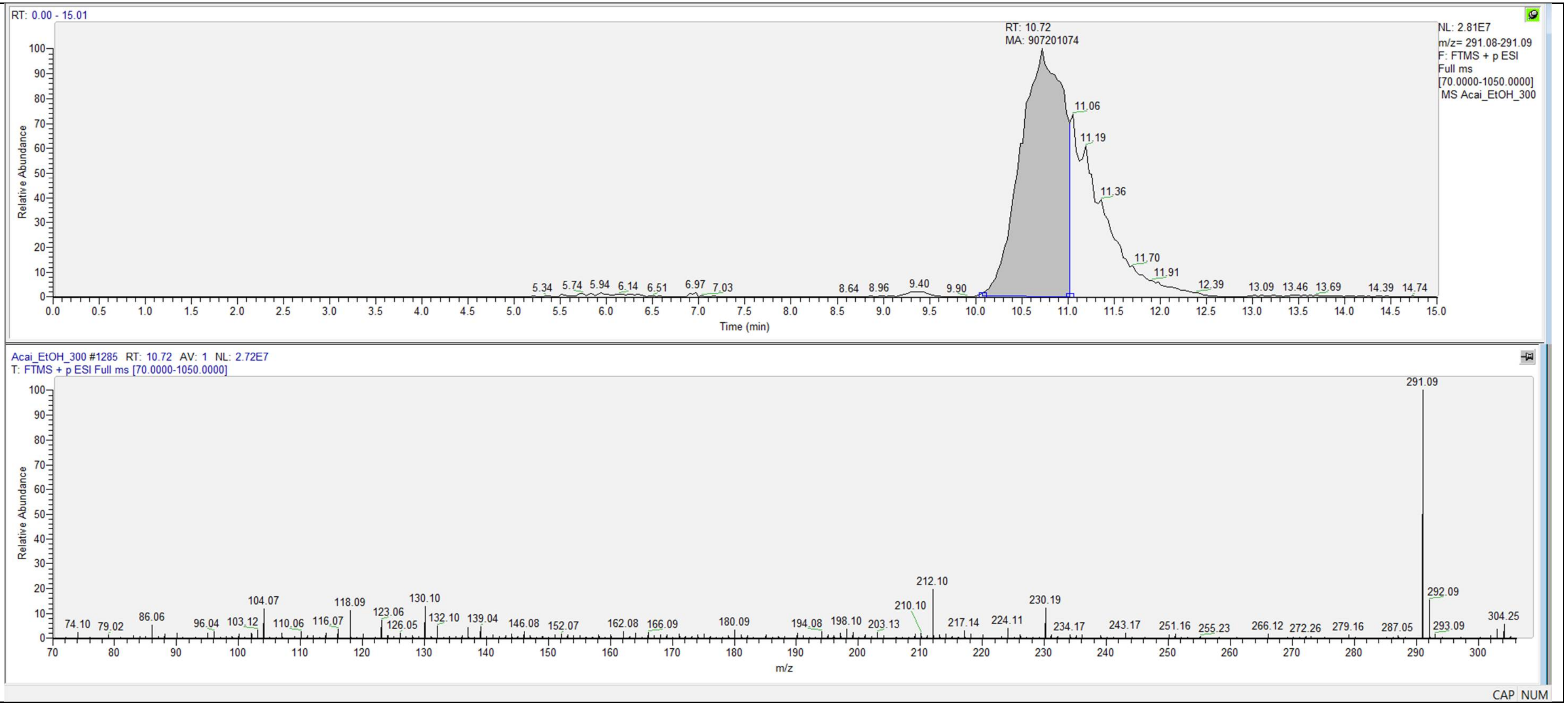

RT: 0.00 - 15.01
RT: 10.72
MA: 907201074
NL: 2.81E7
m/z= 291.08-291.09
F: FTMS + p ESI
Full ms
[70.0000-1050.0000]
MS Acai_EtOH_300
11.06
11.19
11.36
11.70
11.91
12.39
5.34 5.74 5.94 6.14 6.51 6.97 7.03 8.64 8.96 9.40 9.90
13.09 13.46 13.69 14.39 14.74
Relative Abundance
Time (min)
Acai_EtOH_300 #1285 RT: 10.72 AV: 1 NL: 2.72E7
T: FTMS + p ESI Full ms [70.0000-1050.0000]
291.09
292.09
293.09
304.25
212.10
210.10
230.19
104.07
118.09
130.10
123.06
126.05
132.10
139.04
74.10 79.02 86.06 96.04 103.12 110.06 116.07
146.08 152.07 162.08 166.09 180.09 194.08 198.10 203.13
217.14 224.11 234.17 243.17 251.16 255.23 266.12 272.26 279.16 287.05
Relative Abundance
m/z
CAP NUM

(+)-menthiafolic acid

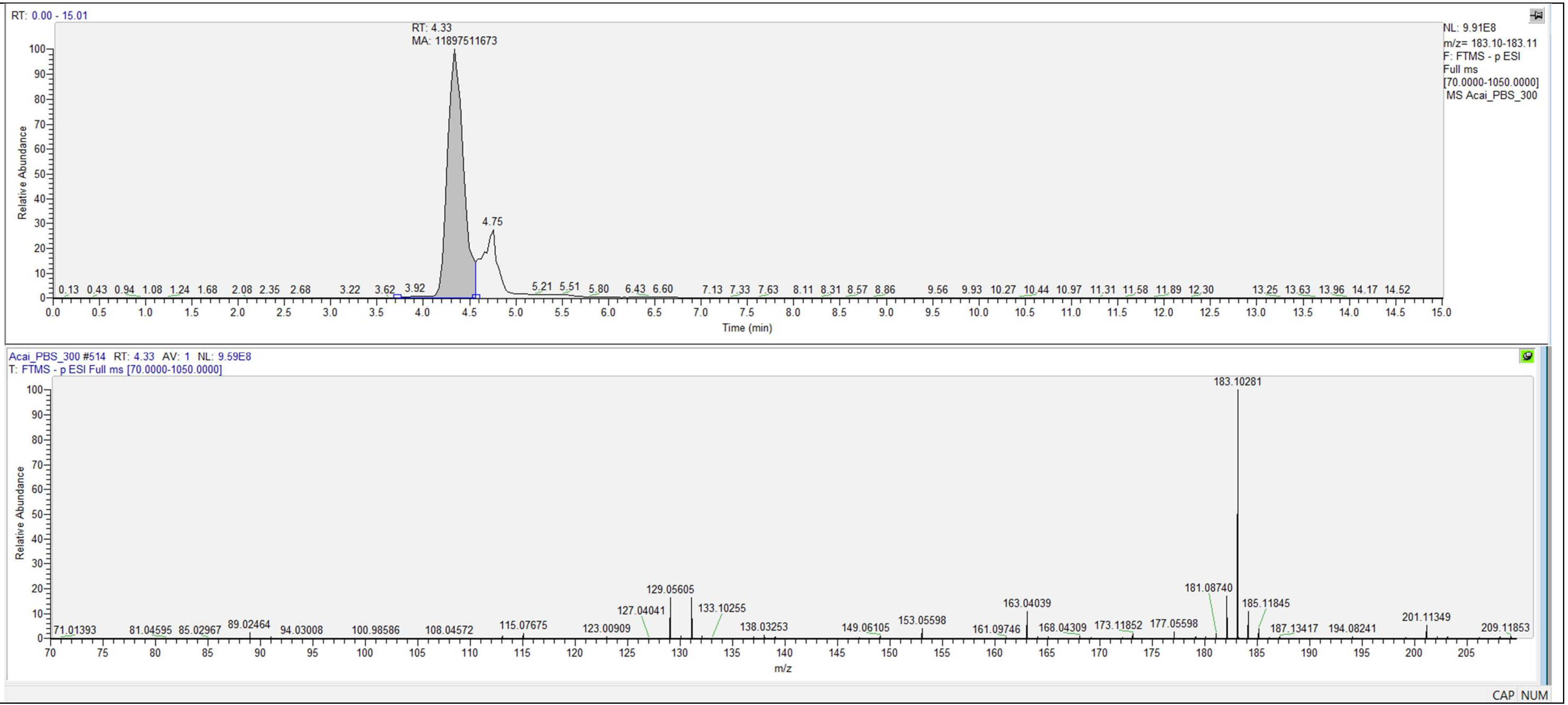

(E,Z)-2,6-dimethyl-2,6-octadiene-1,8-diol

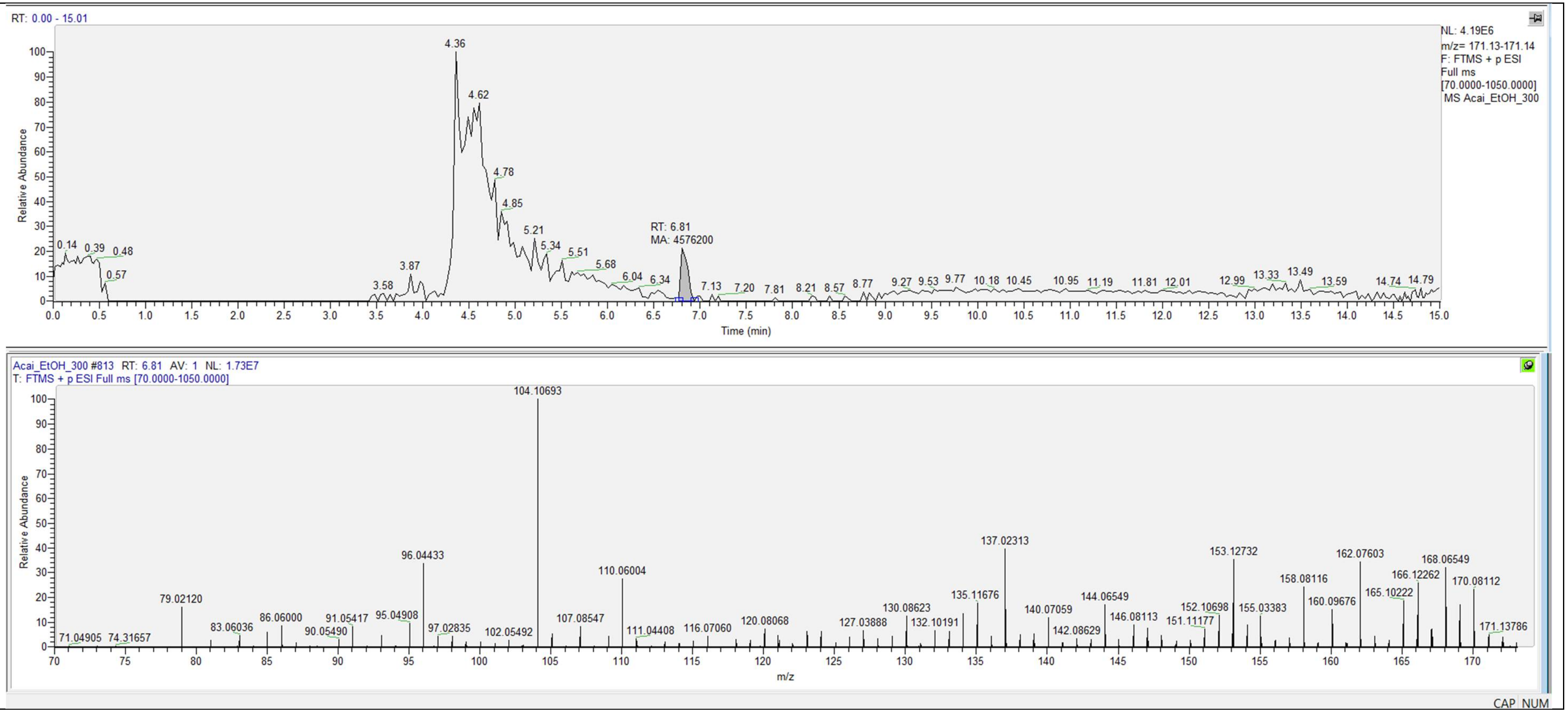

(-)-loliolide	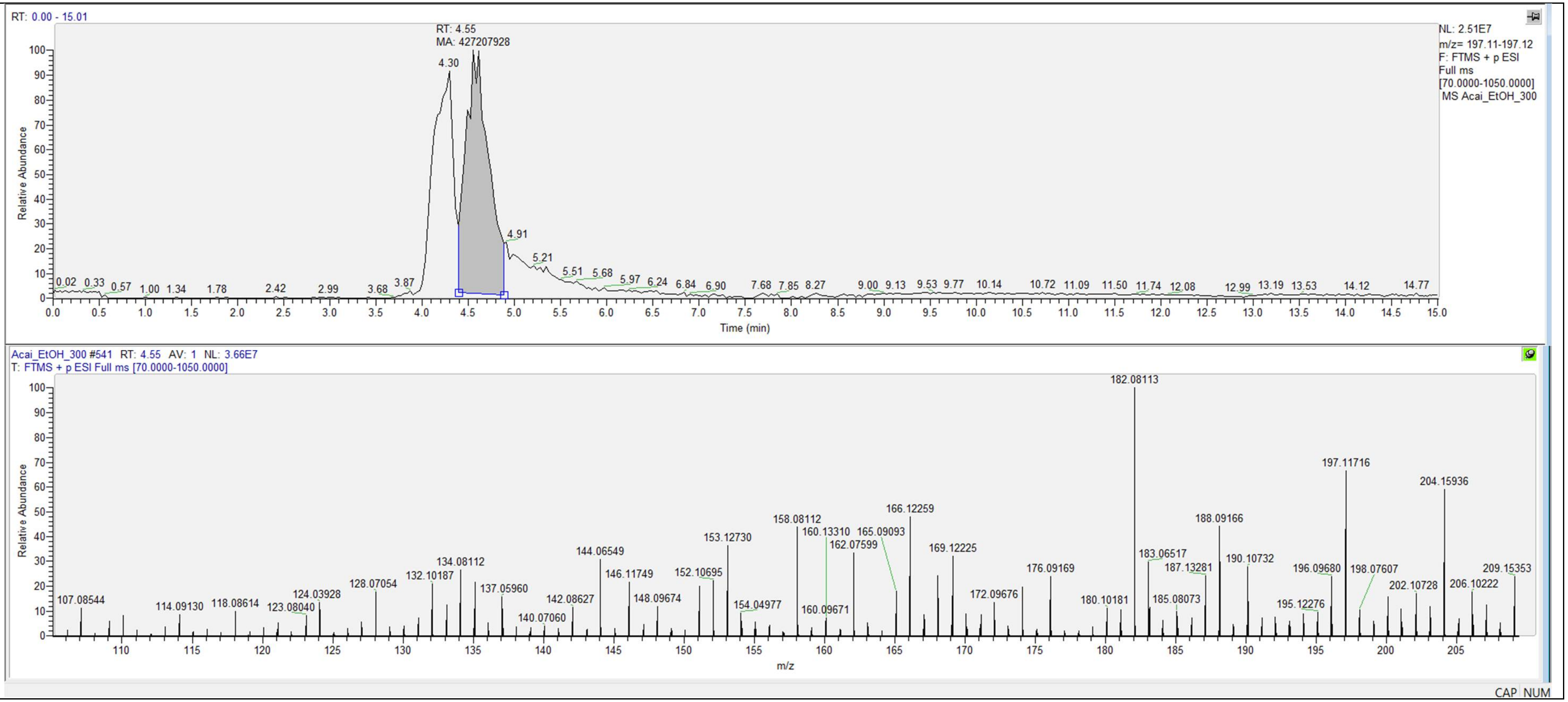 RT: 0.00 - 15.01<br>RT: 4.55<br>MA: 427207928<br>NL: 2.51E7<br>m/z= 197.11-197.12<br>F: FTMS + p ESI<br>Full ms<br>[70.0000-1050.0000]<br>MS Acai_EtOH_300<br>Relative Abundance<br>Time (min)<br>Acai_EtOH_300 #541 RT: 4.55 AV: 1 NL: 3.66E7<br>T: FTMS + p ESI Full ms [70.0000-1050.0000]<br>182.08113<br>197.11716<br>204.15936<br>166.12259<br>188.09166<br>158.08112<br>m/z<br>CAP NUM

Oleic acid	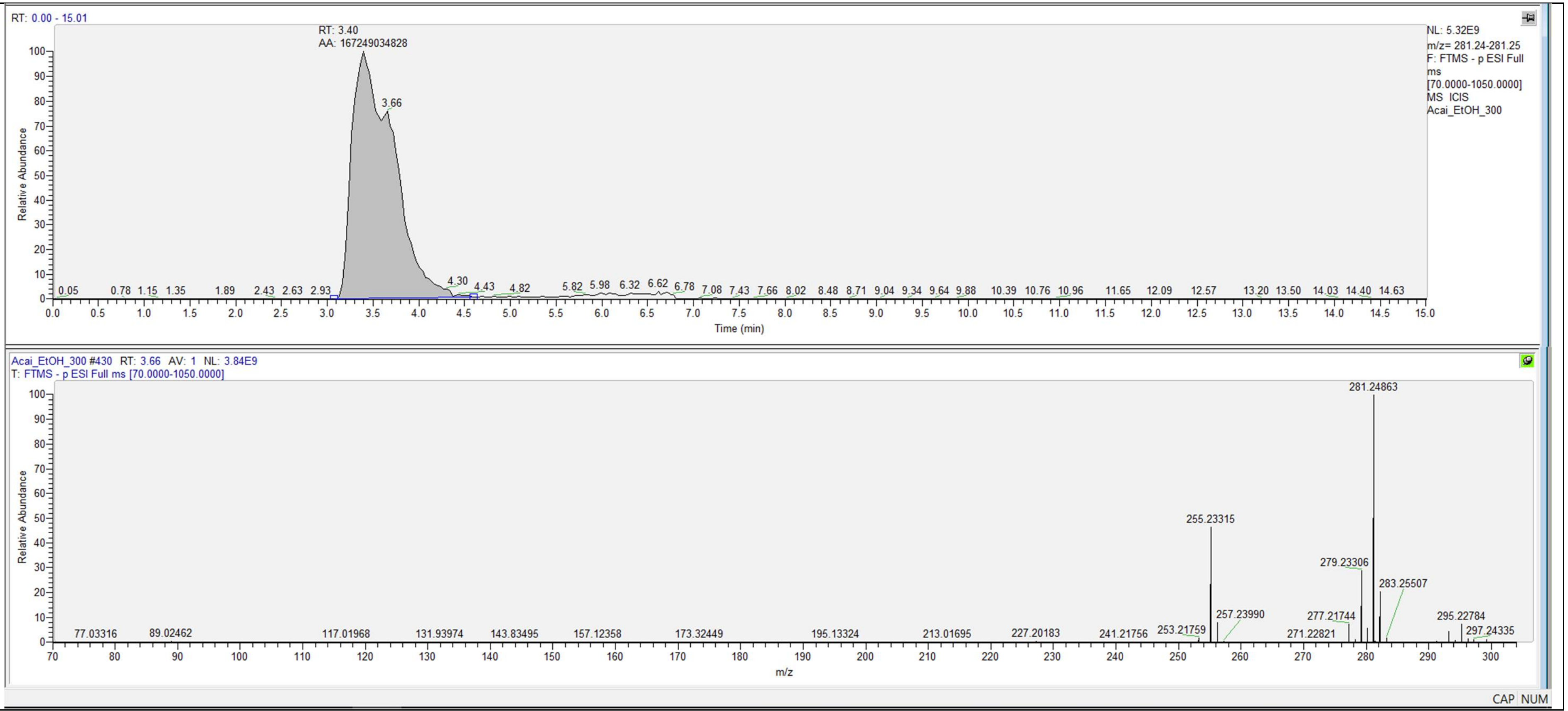

RT: 0.00 - 15.01
RT: 3.40
AA: 167249034828
NL: 5.32E9
m/z= 281.24-281.25
F: FTMS - p ESI Full ms [70.0000-1050.0000] MS ICIS Acai_EtOH_300
Relative Abundance
Time (min)
Acai_EtOH_300 #430 RT: 3.66 AV: 1 NL: 3.84E9
T: FTMS - p ESI Full ms [70.0000-1050.0000]
281.24863
255.23315
279.23306
283.25507
295.22784
Relative Abundance
m/z

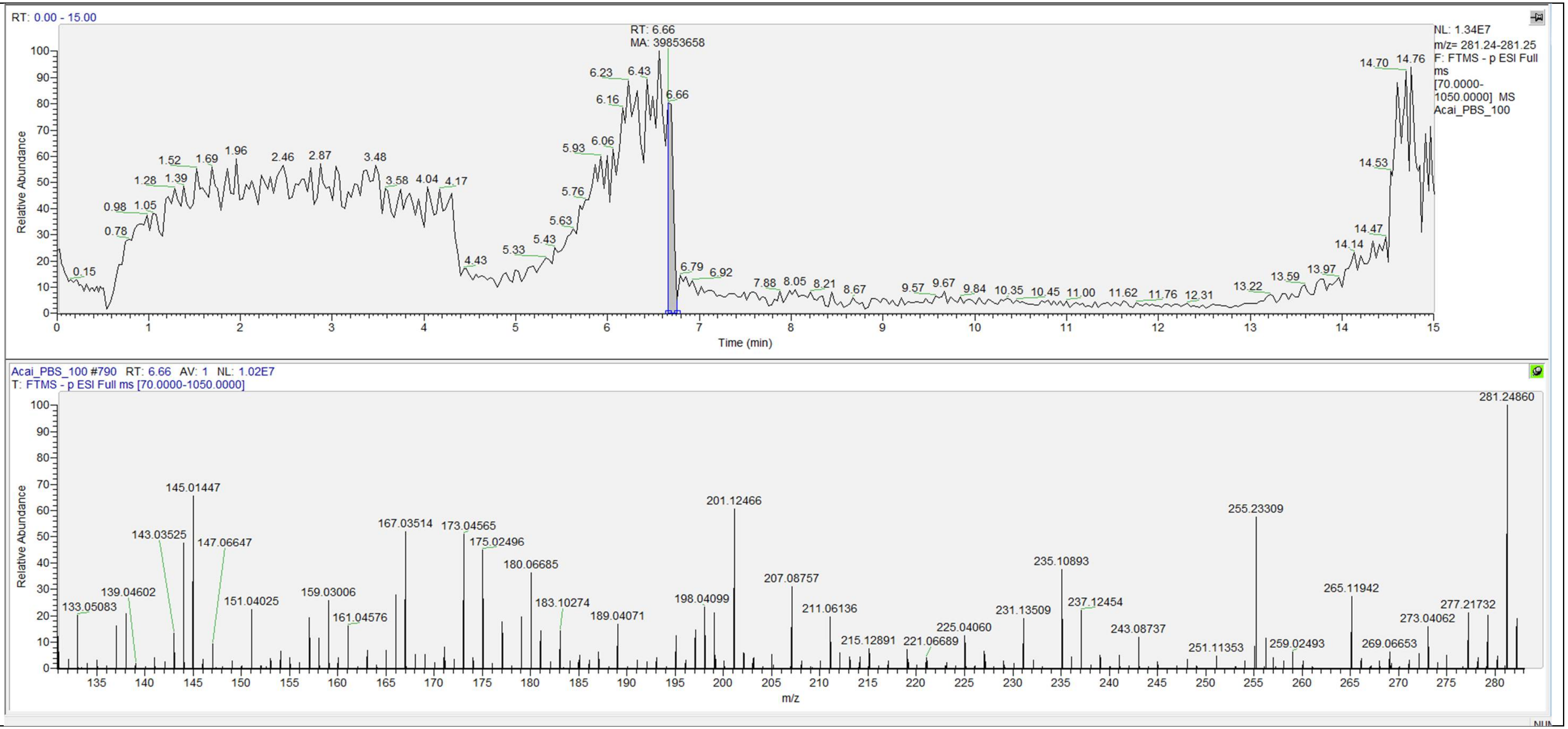
RT: 0.00 - 15.00
RT: 6.66
MA: 39853658
NL: 1.34E7
m/z= 281.24-281.25
F: FTMS - p ESI Full ms [70.0000-1050.0000] MS
Acai_PBS_100
Relative Abundance
Time (min)
0.15
0.78
0.98
1.05
1.28
1.39
1.52
1.69
1.96
2.46
2.87
3.48
3.58
4.04
4.17
4.43
5.33
5.43
5.63
5.76
5.93
6.06
6.16
6.23
6.43
6.66
6.79
6.92
7.88
8.05
8.21
8.67
9.57
9.67
9.84
10.35
10.45
11.00
11.62
11.76
12.31
13.22
13.59
13.97
14.14
14.47
14.53
14.70
14.76
Acai_PBS_100 #790 RT: 6.66 AV: 1 NL: 1.02E7
T: FTMS - p ESI Full ms [70.0000-1050.0000]
Relative Abundance
m/z
133.05083
139.04602
143.03525
145.01447
147.06647
151.04025
159.03006
161.04576
167.03514
173.04565
175.02496
180.06685
183.10274
189.04071
198.04099
201.12466
207.08757
211.06136
215.12891
221.06689
225.04060
231.13509
235.10893
237.12454
243.08737
251.11353
255.23309
259.02493
265.11942
269.06653
273.04062
277.21732
281.24860

Palmitoleic acid

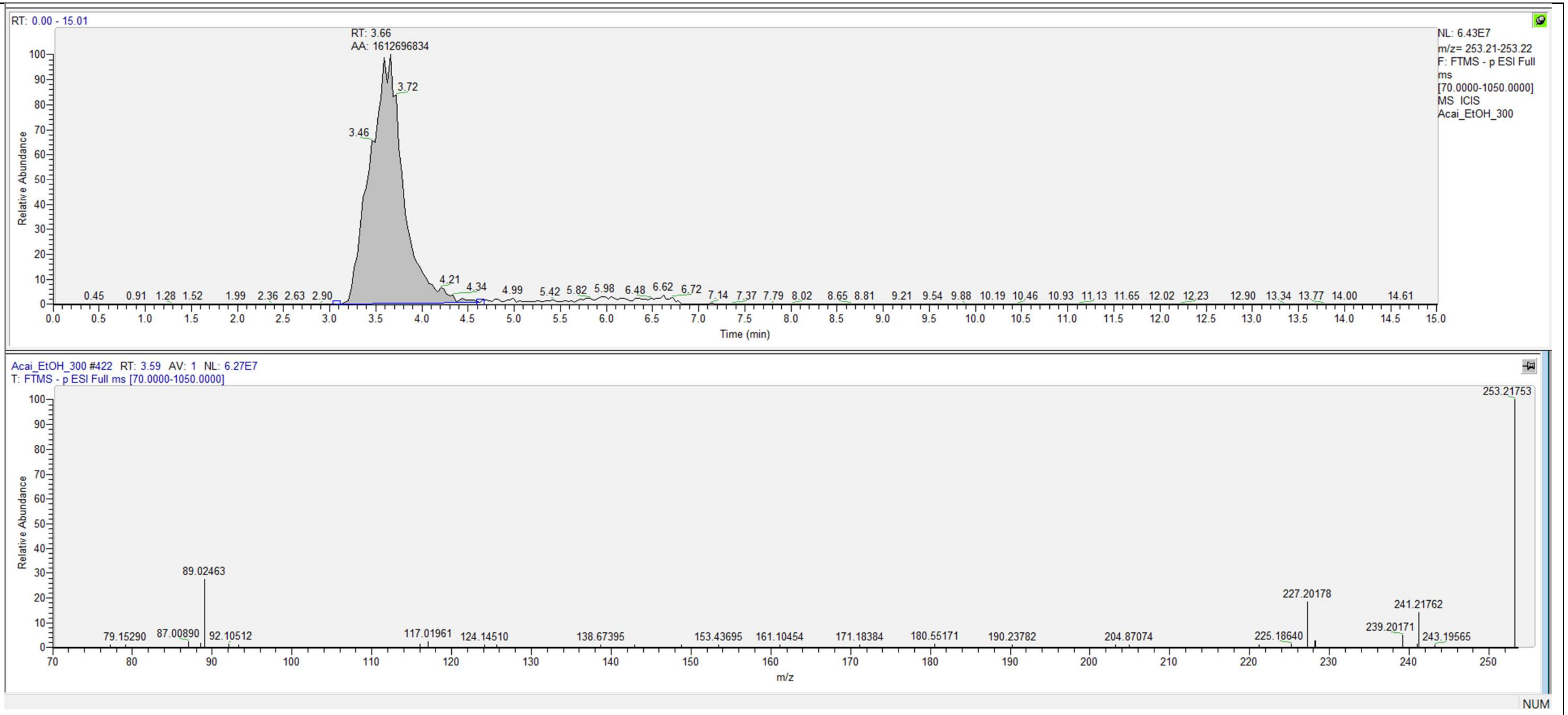

Linoleic acid	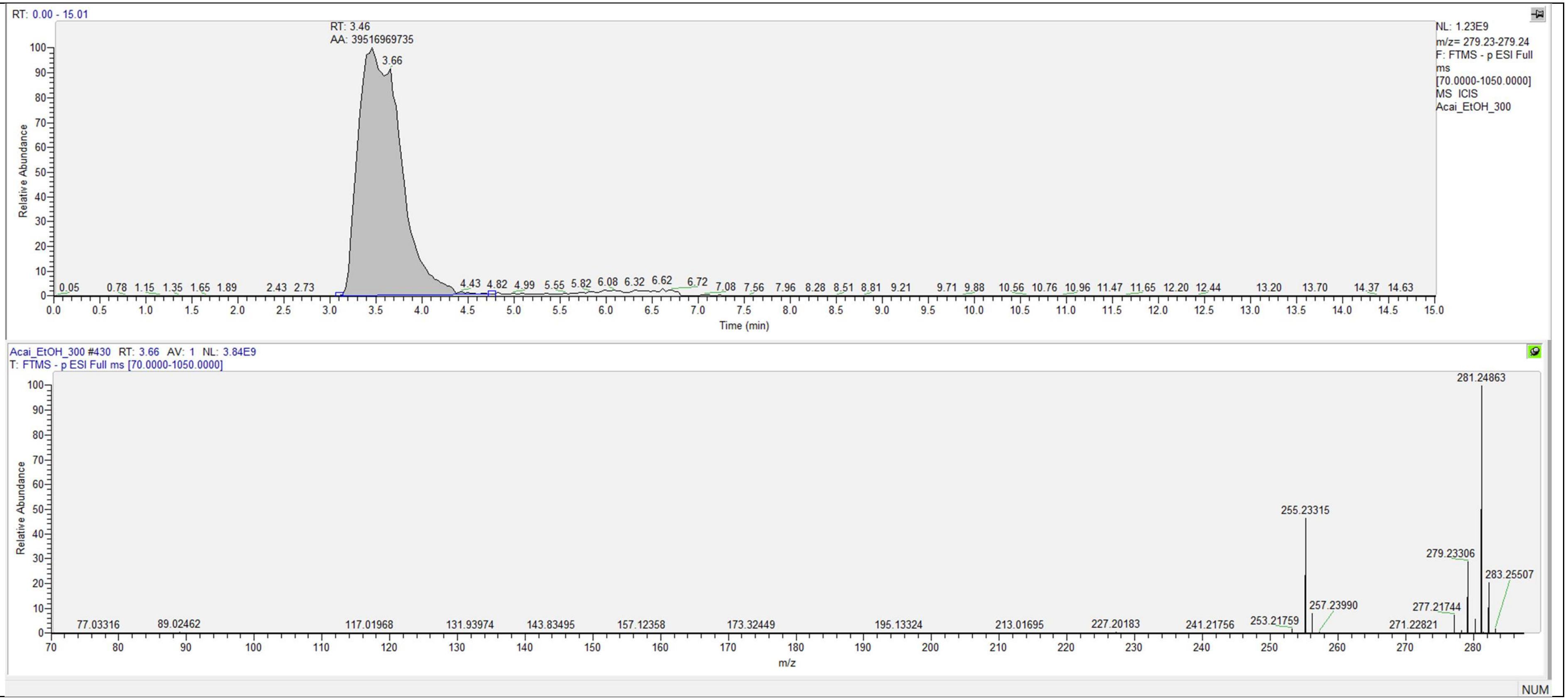

Linolenic acid	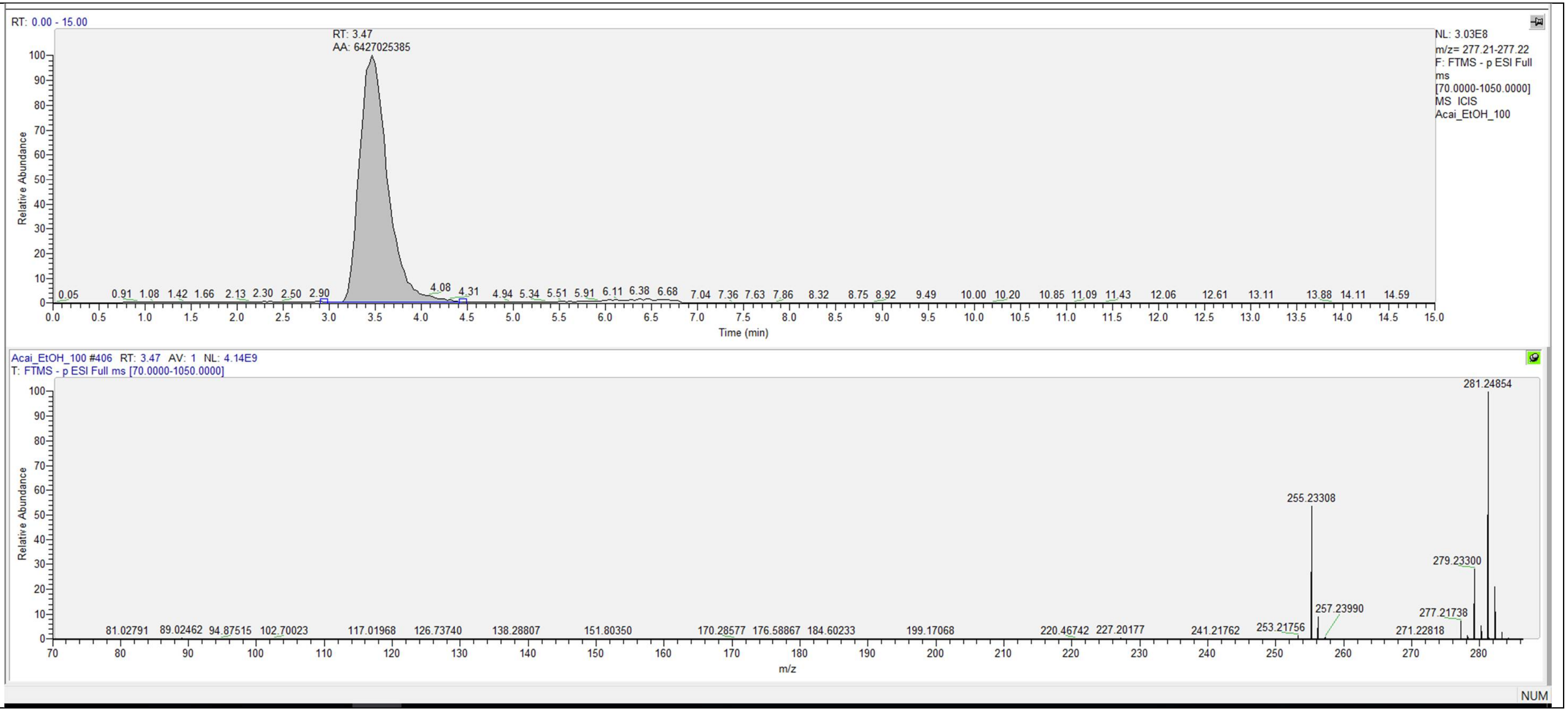

Palmitic acid	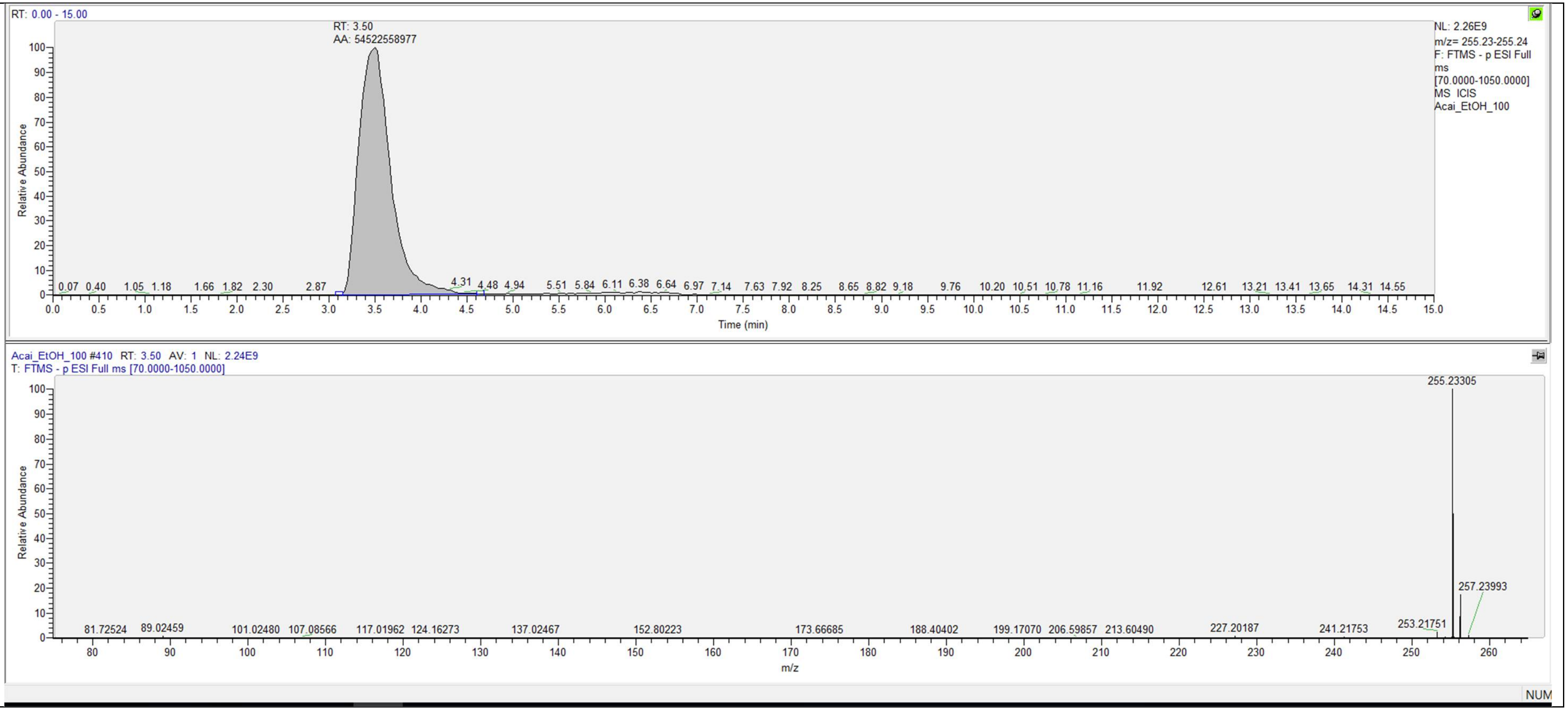

Stearic acid	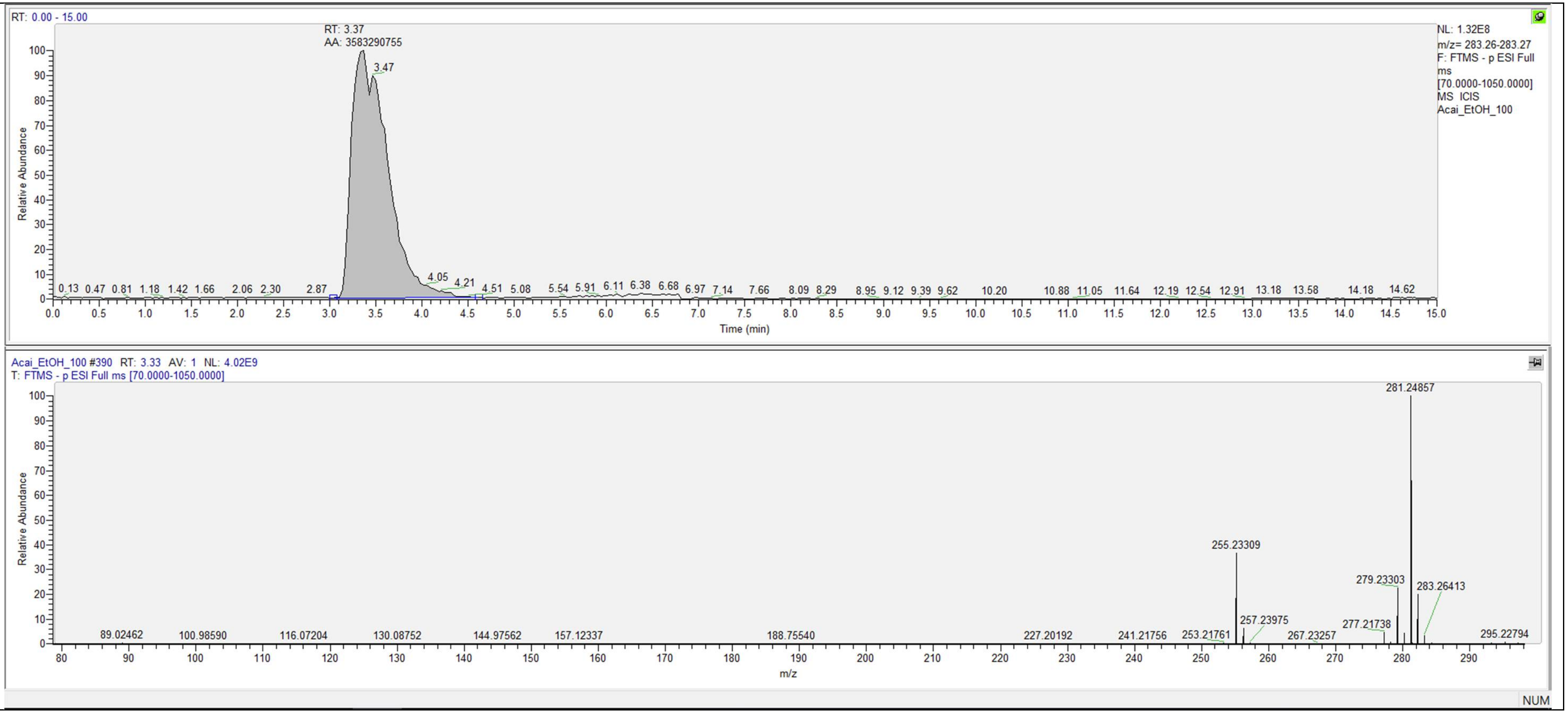

Alanine

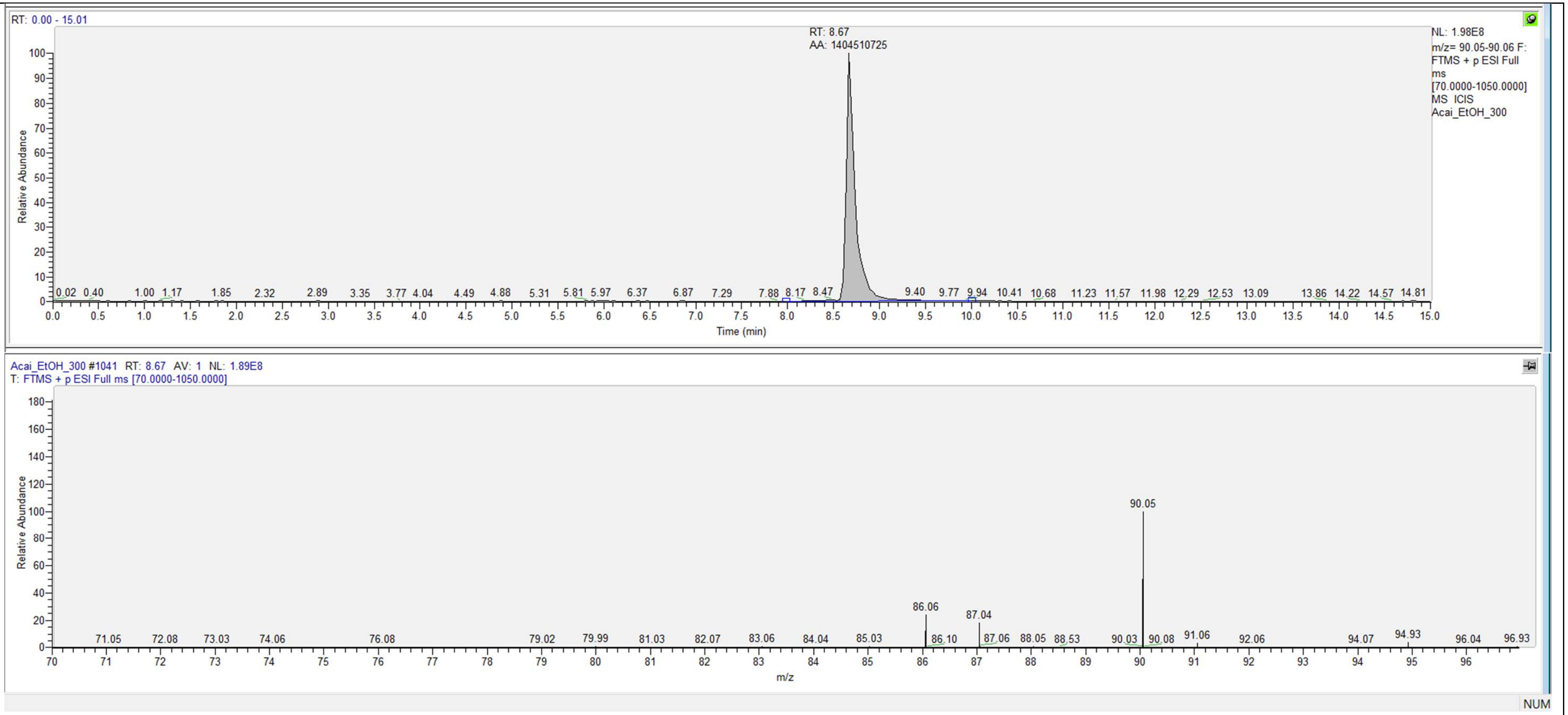
RT: 0.00 - 15.01
RT: 8.67
AA: 1404510725
NL: 1.98E8
m/z= 90.05-90.06 F: FTMS + p ESI Full ms [70.0000-1050.0000] MS ICIS Acai_EtOH_300
Relative Abundance
Time (min)
Acai_EtOH_300 #1041 RT: 8.67 AV: 1 NL: 1.89E8
T: FTMS + p ESI Full ms [70.0000-1050.0000]
90.05
86.06
87.04
m/z
NUM

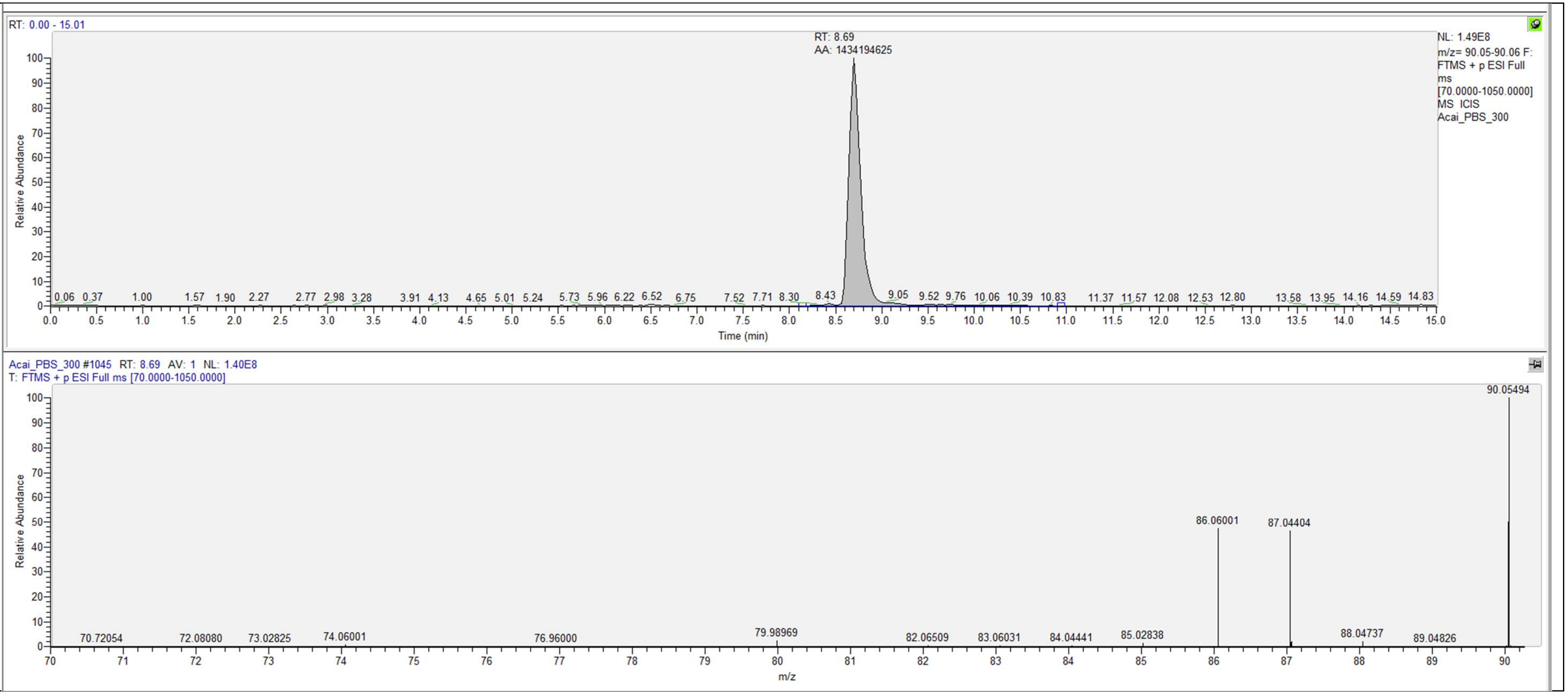

RT: 0.00 - 15.01
RT: 8.69
AA: 1434194625
NL: 1.49E8
m/z= 90.05-90.06 F: FTMS + p ESI Full ms [70.0000-1050.0000] MS ICIS Acai_PBS_300
Relative Abundance
Time (min)
Acai_PBS_300 #1045 RT: 8.69 AV: 1 NL: 1.40E8
T: FTMS + p ESI Full ms [70.0000-1050.0000]
90.05494
86.06001
87.04404
70.72054
72.08080
73.02825
74.06001
76.96000
79.98969
82.06509
83.06031
84.04441
85.02838
88.04737
89.04826
m/z

Lysine	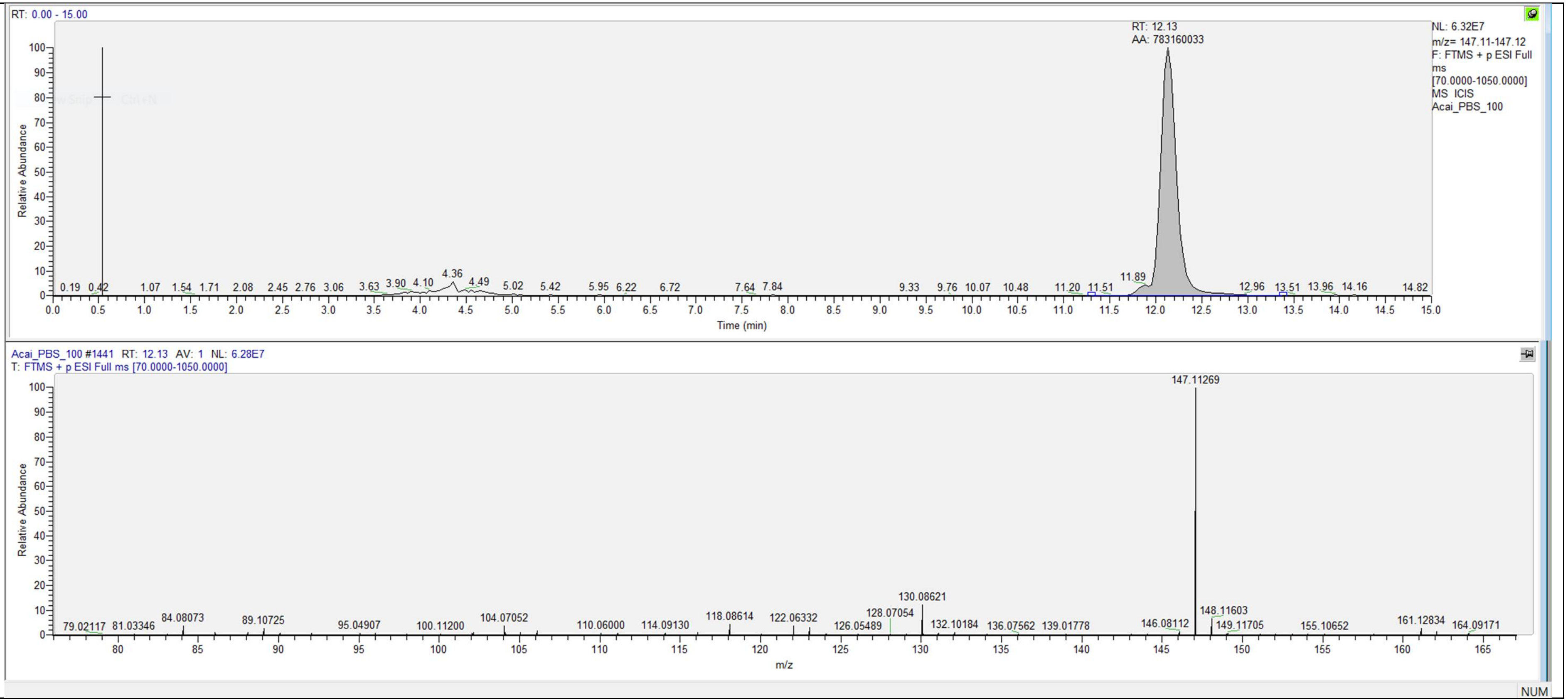

RT: 0.00 - 15.00
RT: 12.13
AA: 783160033
NL: 6.32E7
m/z= 147.11-147.12
F: FTMS + p ESI Full ms [70.0000-1050.0000]
MS ICIS
Acai_PBS_100
Relative Abundance
Time (min)
Acai_PBS_100 #1441 RT: 12.13 AV: 1 NL: 6.28E7
T: FTMS + p ESI Full ms [70.0000-1050.0000]
147.11269
130.08621
148.11603
128.07054
84.08073
89.10725
104.07052
118.08614
122.06332
161.12834
m/z

Arginine	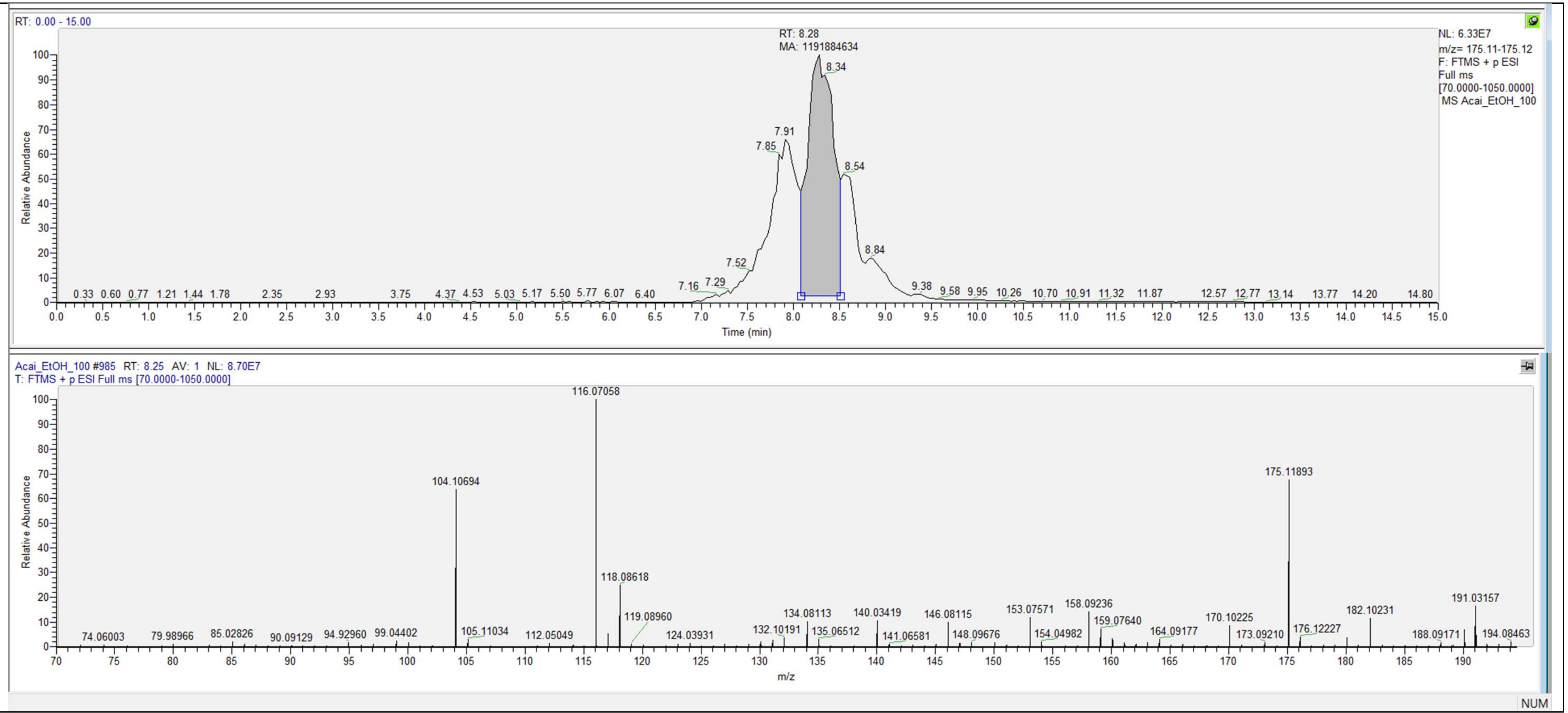

RT: 0.00 - 15.00
RT: 8.28
MA: 1191884634
NL: 6.33E7
m/z= 175.11-175.12
F: FTMS + p ESI
Full ms
[70.0000-1050.0000]
MS Acai_EtOH_100
Relative Abundance
Time (min)
Acai_EtOH_100 #985 RT: 8.25 AV: 1 NL: 8.70E7
T: FTMS + p ESI Full ms [70.0000-1050.0000]
116.07058
104.10694
175.11893
118.08618
191.03157
m/z
NUM

<table>
<tr>
<td>Methionine</td>
<td>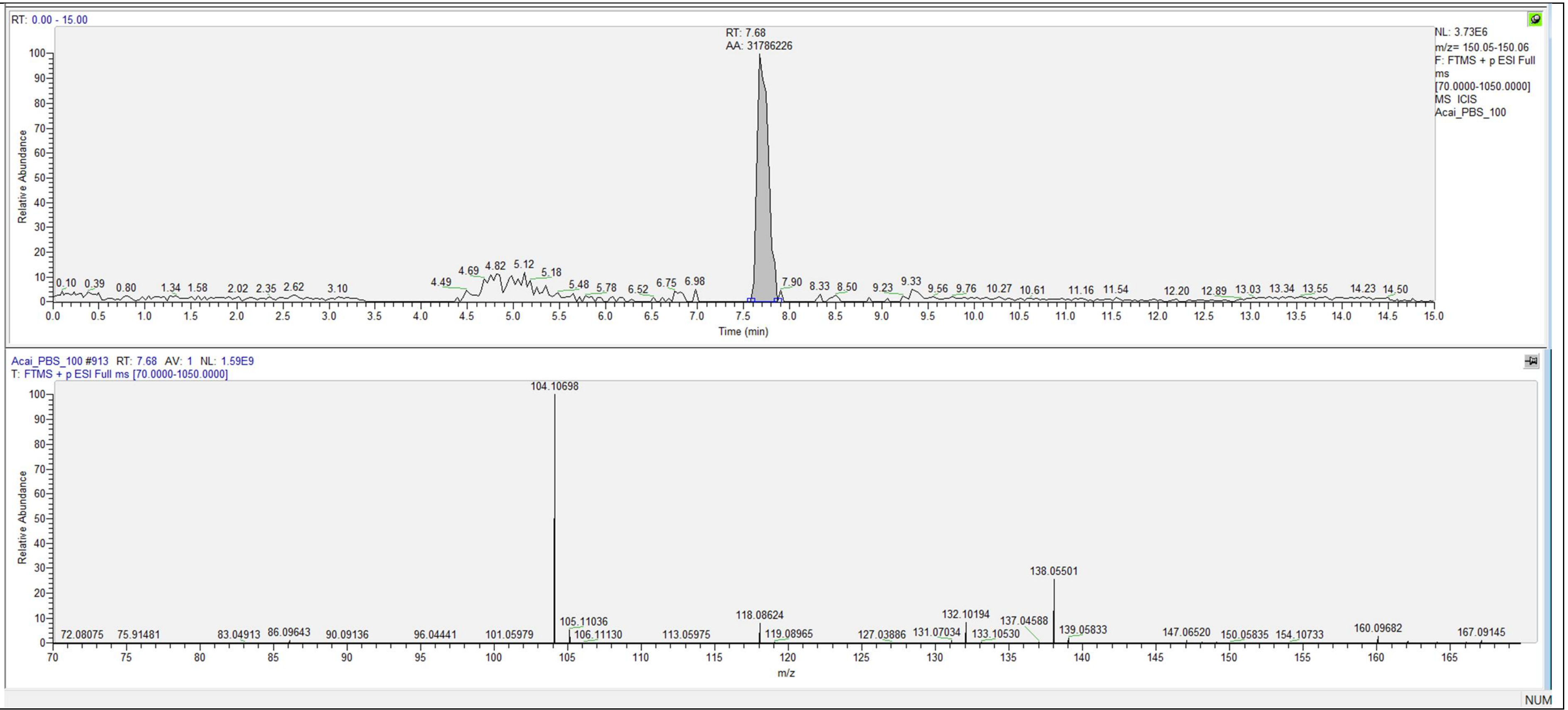
</td>
</tr>
</table>

Phenylalanine

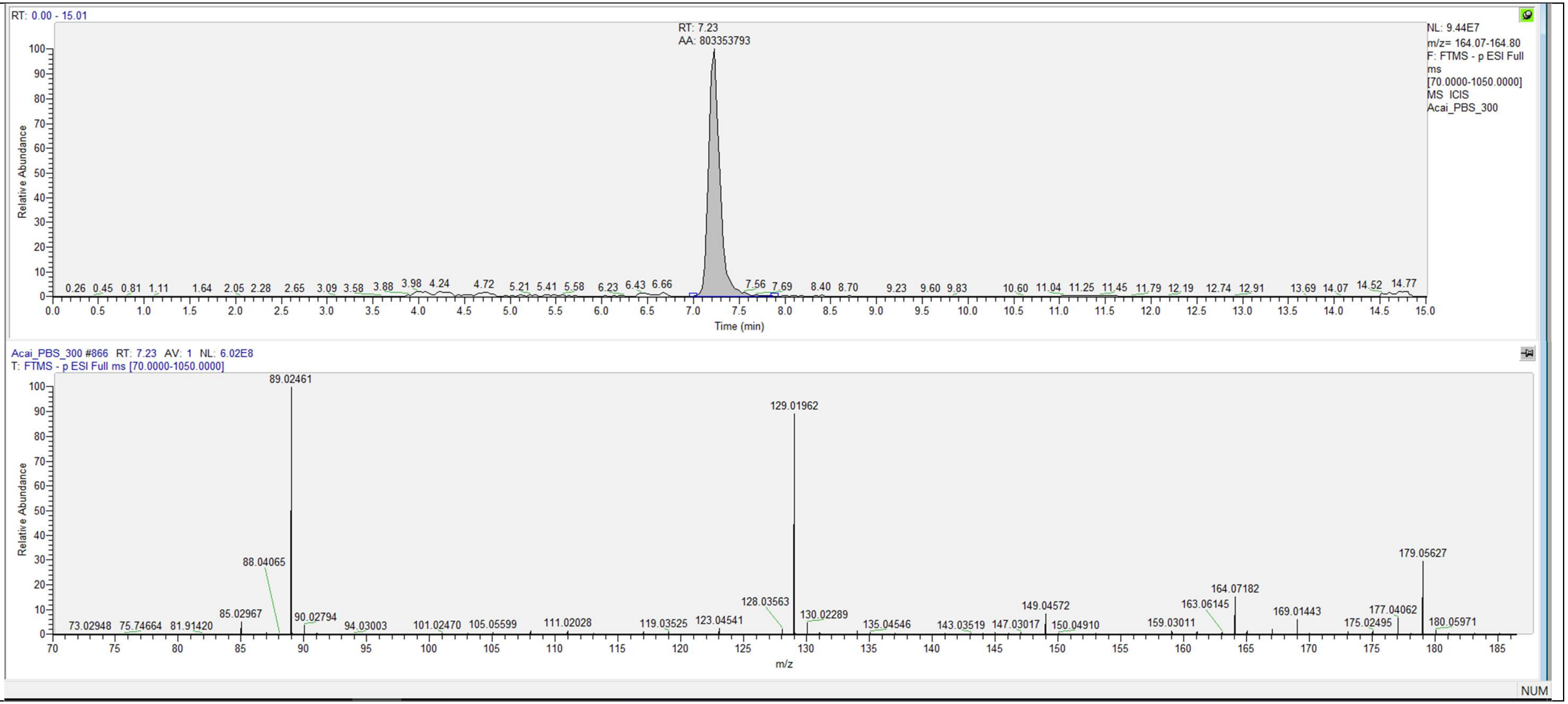

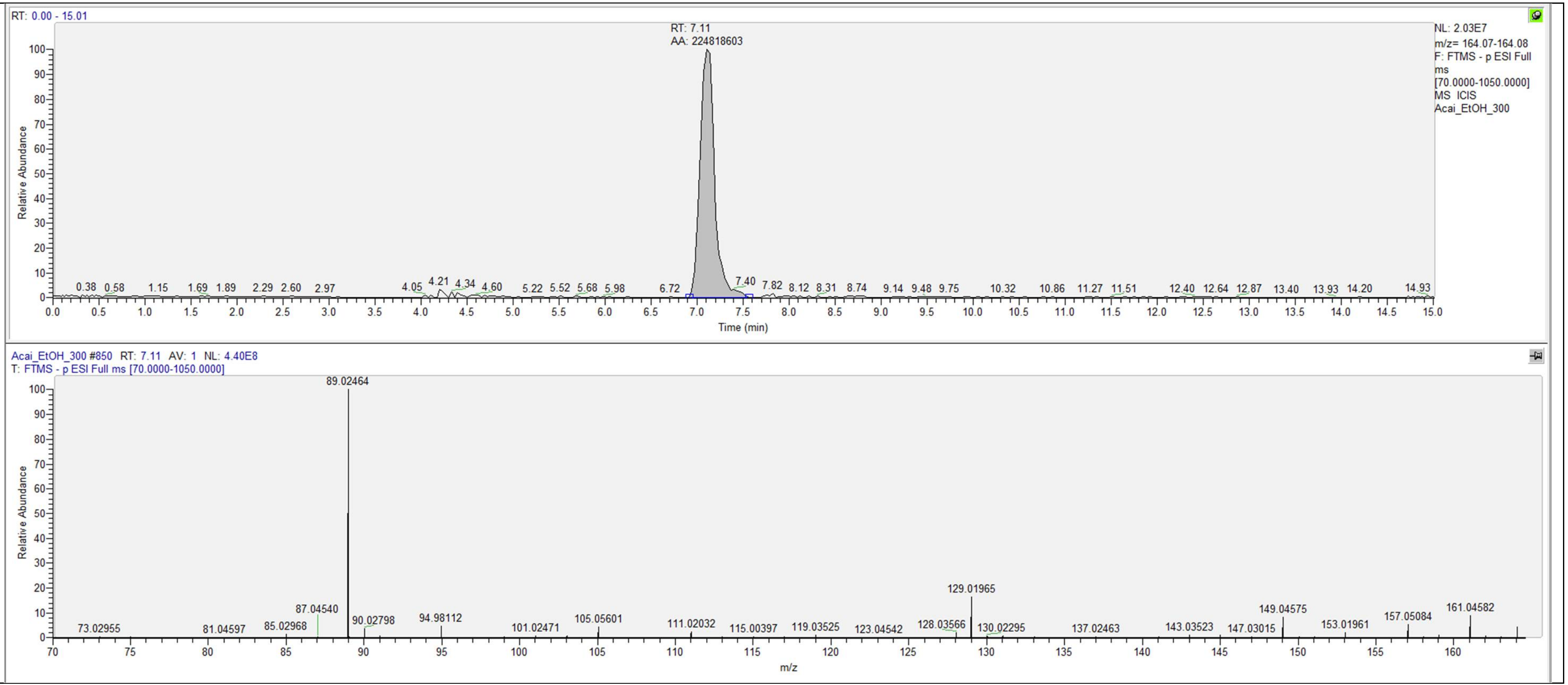
RT: 0.00 - 15.01
RT: 7.11
AA: 224818603
NL: 2.03E7
m/z= 164.07-164.08
F: FTMS - p ESI Full ms [70.0000-1050.0000]
MS ICIS
Acai_EtOH_300
Relative Abundance
Time (min)
Acai_EtOH_300 #850 RT: 7.11 AV: 1 NL: 4.40E8
T: FTMS - p ESI Full ms [70.0000-1050.0000]
89.02464
129.01965
149.04575
161.04582
157.05084
87.04540
m/z

Proline

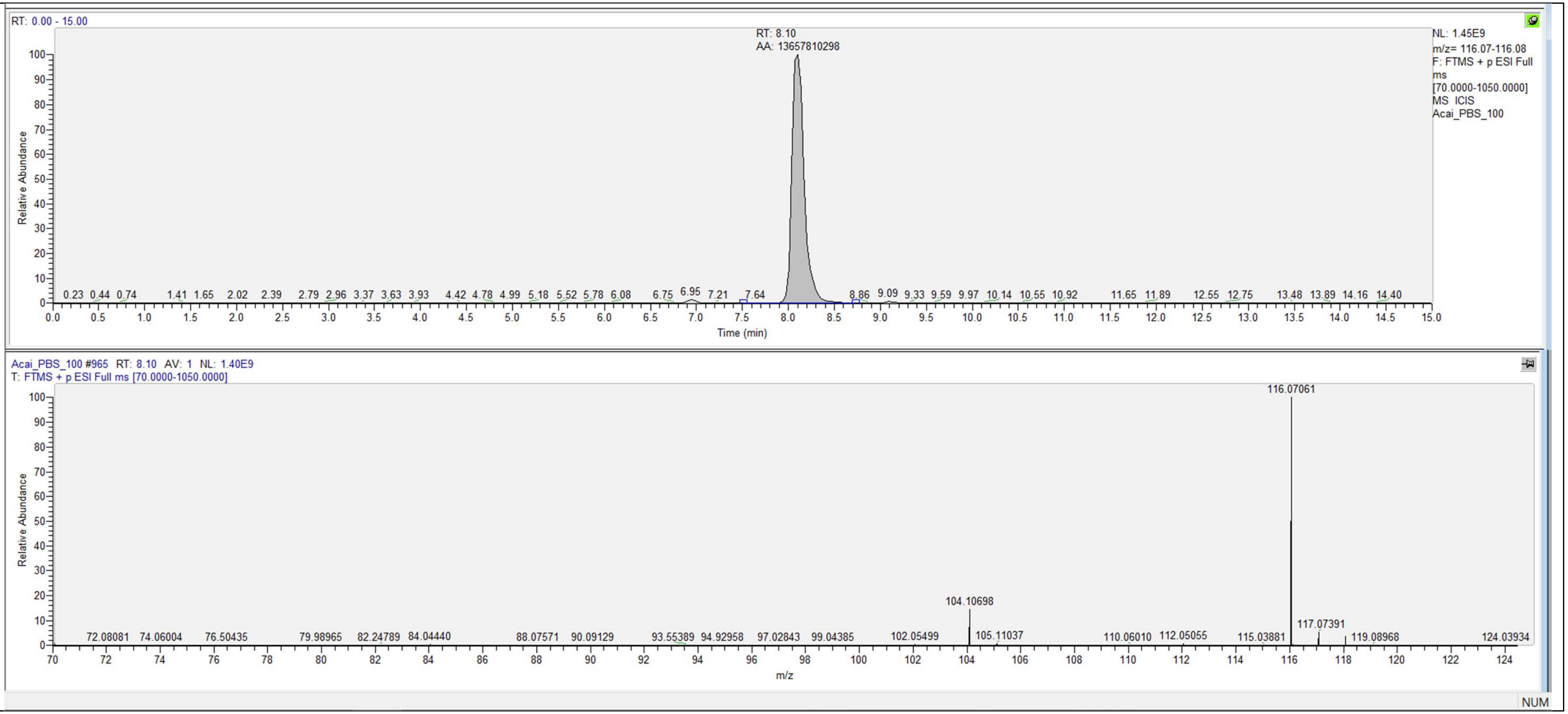
RT: 0.00 - 15.00
RT: 8.10
AA: 13657810298
NL: 1.45E9
m/z= 116.07-116.08
F: FTMS + p ESI Full ms [70.0000-1050.0000]
MS ICIS
Acai_PBS_100
Relative Abundance
Time (min)
Acai_PBS_100 #965 RT: 8.10 AV: 1 NL: 1.40E9
T: FTMS + p ESI Full ms [70.0000-1050.0000]
116.07061
104.10698
105.11037
117.07391
119.08968
72.08081
74.06004
76.50435
79.98965
82.24789
84.04440
88.07571
90.09129
93.55389
94.92958
97.02843
99.04385
102.05499
110.06010
112.05055
115.03881
124.03934
m/z
NUM

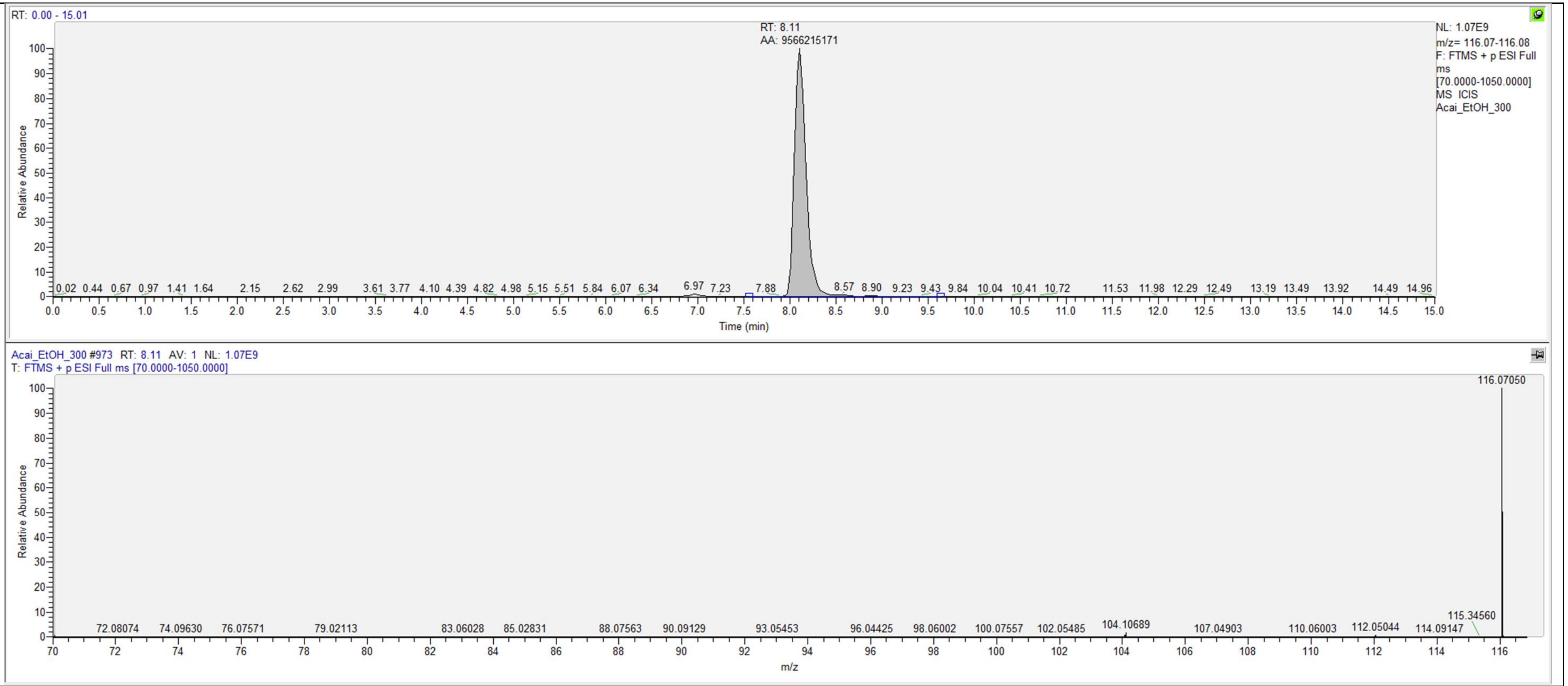
RT: 0.00 - 15.01
RT: 8.11
AA: 9566215171
NL: 1.07E9
m/z= 116.07-116.08
F: FTMS + p ESI Full ms [70.0000-1050.0000]
MS ICIS
Acai_EtOH_300
Relative Abundance
Time (min)
Acai_EtOH_300 #973 RT: 8.11 AV: 1 NL: 1.07E9
T: FTMS + p ESI Full ms [70.0000-1050.0000]
116.07050
115.34560
104.10689
m/z

Glutamic acid	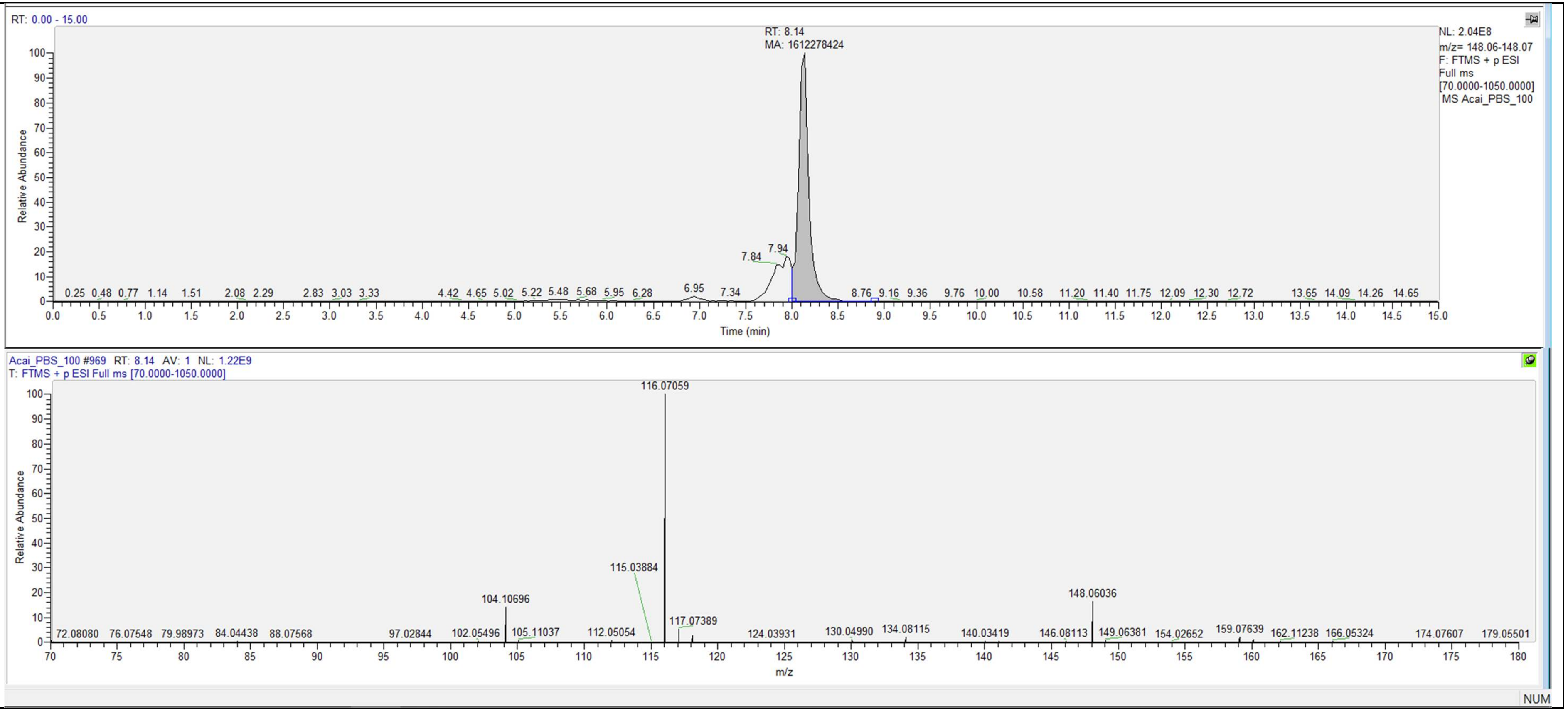

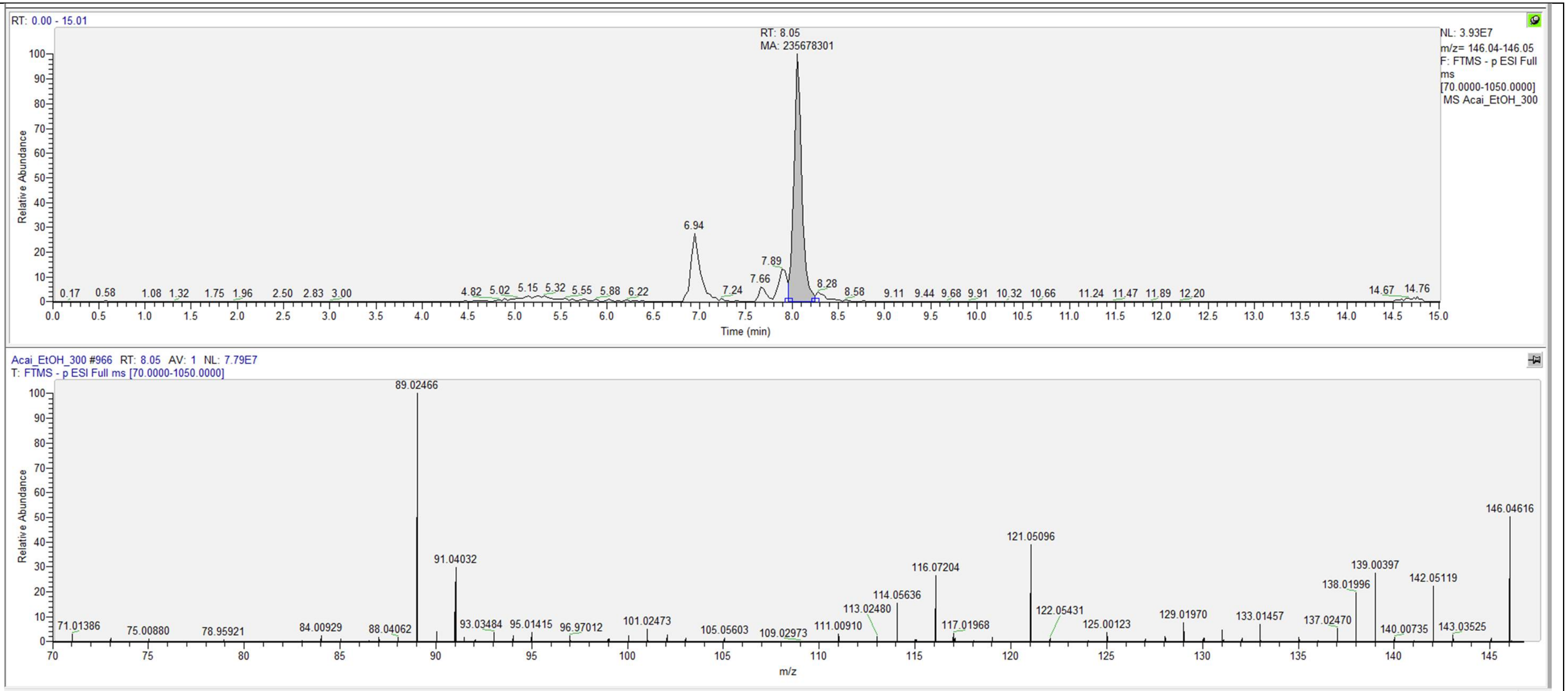

RT: 0.00 - 15.01
RT: 8.05
MA: 235678301
NL: 3.93E7
m/z= 146.04-146.05
F: FTMS - p ESI Full ms [70.0000-1050.0000]
MS Acai_EtOH_300
Relative Abundance
Time (min)
Acai_EtOH_300 #966 RT: 8.05 AV: 1 NL: 7.79E7
T: FTMS - p ESI Full ms [70.0000-1050.0000]
89.02466
91.04032
146.04616
121.05096
116.07204
139.00397
142.05119
138.01996
114.05636
113.02480
m/z

Serine	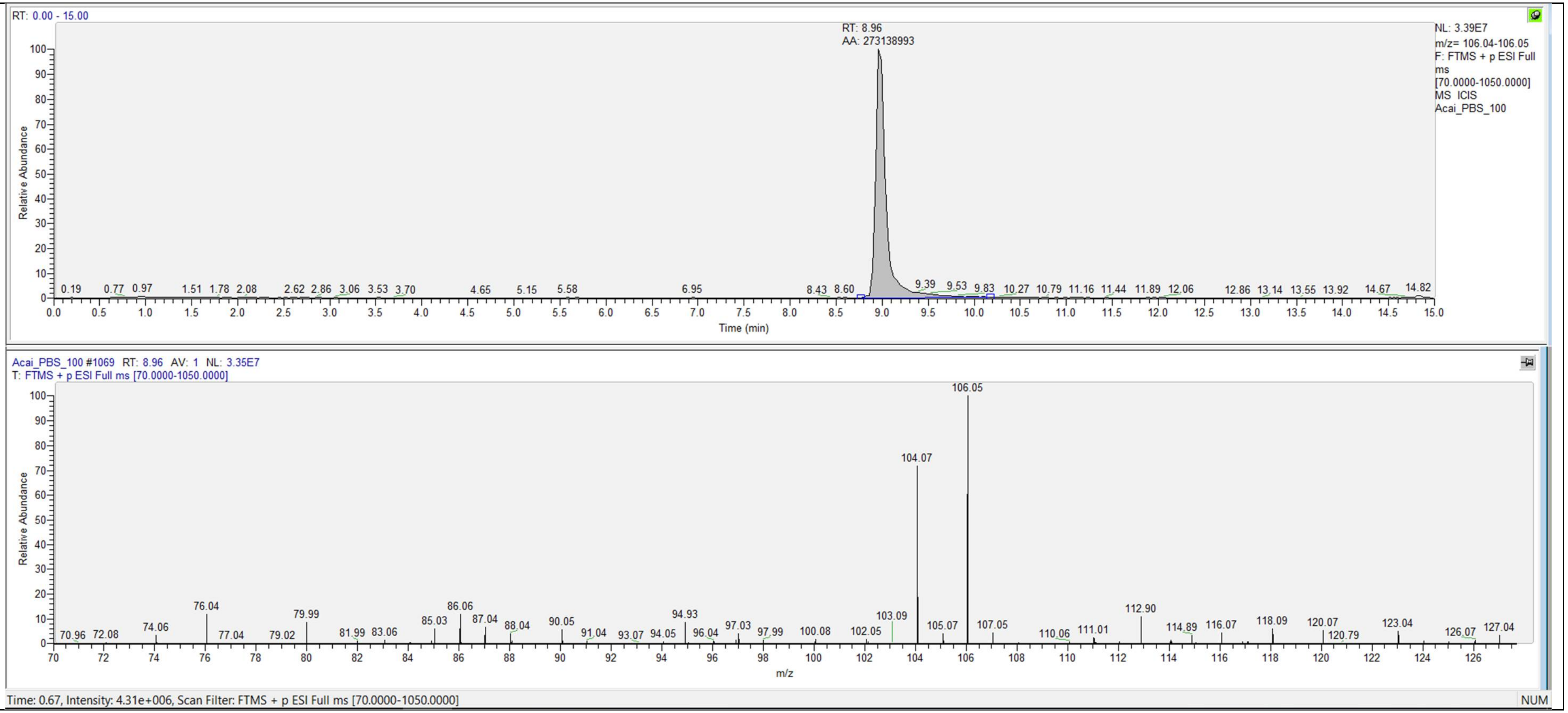

RT: 0.00 - 15.00
RT: 8.96
AA: 273138993
NL: 3.39E7
m/z= 106.04-106.05
F: FTMS + p ESI Full ms [70.0000-1050.0000]
MS ICIS
Acai_PBS_100
Relative Abundance
Time (min)
Acai_PBS_100 #1069 RT: 8.96 AV: 1 NL: 3.35E7
T: FTMS + p ESI Full ms [70.0000-1050.0000]
106.05
104.07
112.90
86.06
76.04
79.99
94.93
103.09
m/z
Time: 0.67, Intensity: 4.31e+006, Scan Filter: FTMS + p ESI Full ms [70.0000-1050.0000]
NUM

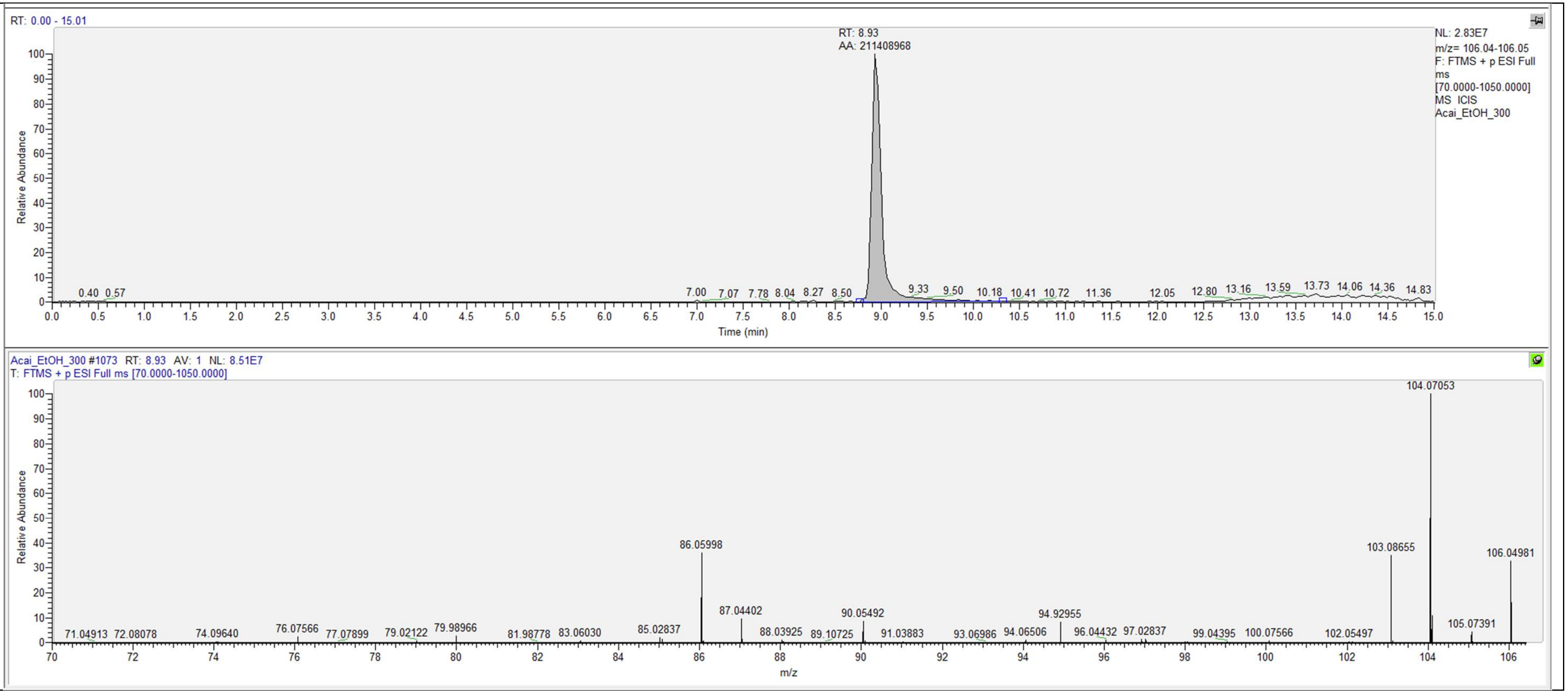

RT: 0.00 - 15.01
RT: 8.93
AA: 211408968
NL: 2.83E7
m/z= 106.04-106.05
F: FTMS + p ESI Full ms [70.0000-1050.0000] MS ICIS Acai_EtOH_300
Relative Abundance
Time (min)
Acai_EtOH_300 #1073 RT: 8.93 AV: 1 NL: 8.51E7
T: FTMS + p ESI Full ms [70.0000-1050.0000]
104.07053
86.05998
103.08655
106.04981
87.04402
90.05492
94.92955
105.07391
m/z

Glycine	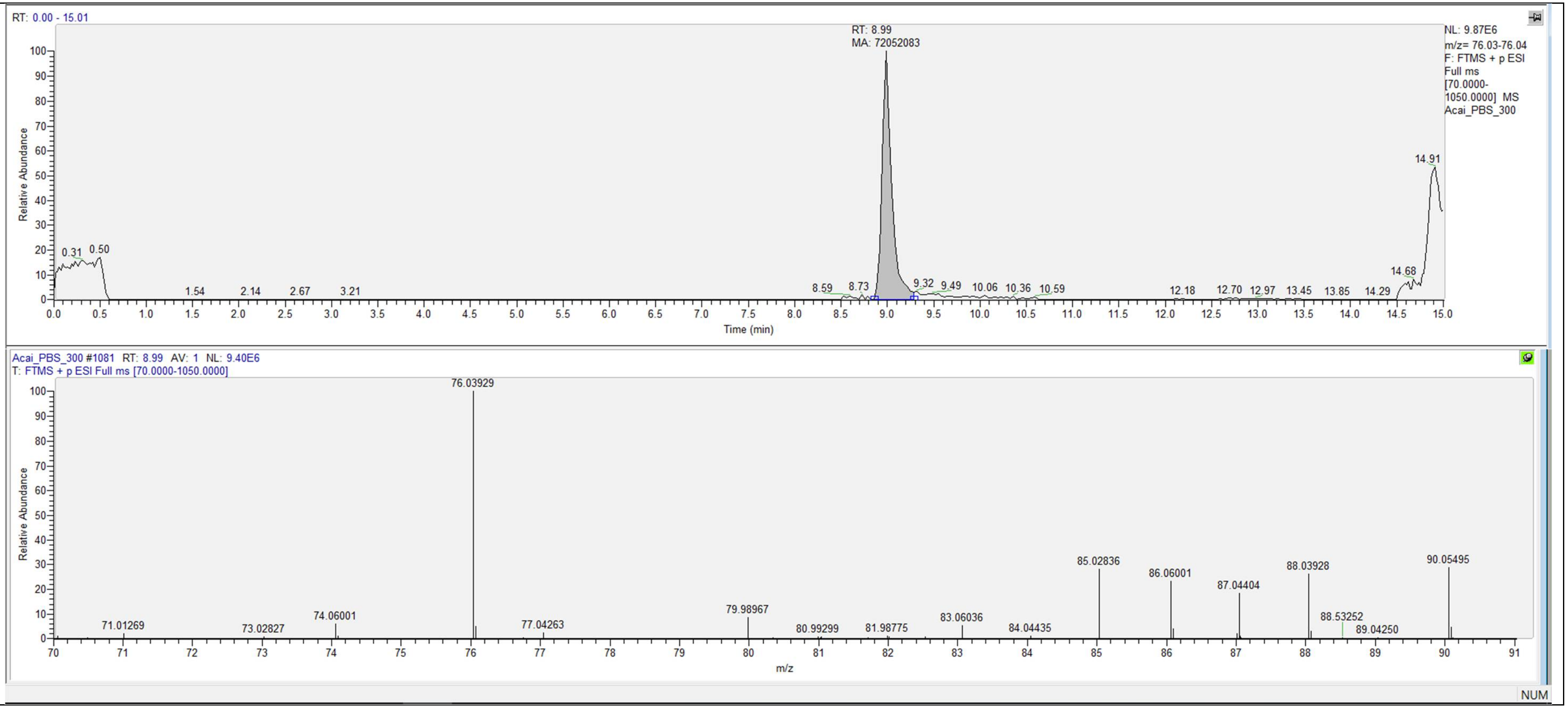RT: 0.00 - 15.01
RT: 8.99
MA: 72052083
NL: 9.87E6
m/z= 76.03-76.04
F: FTMS + p ESI Full ms [70.0000-1050.0000] MS
Acai_PBS_300
Relative Abundance
Time (min)
Acai_PBS_300 #1081 RT: 8.99 AV: 1 NL: 9.40E6
T: FTMS + p ESI Full ms [70.0000-1050.0000]
76.03929
85.02836
86.06001
87.04404
88.03928
90.05495
79.98967
74.06001
m/z
NUM

	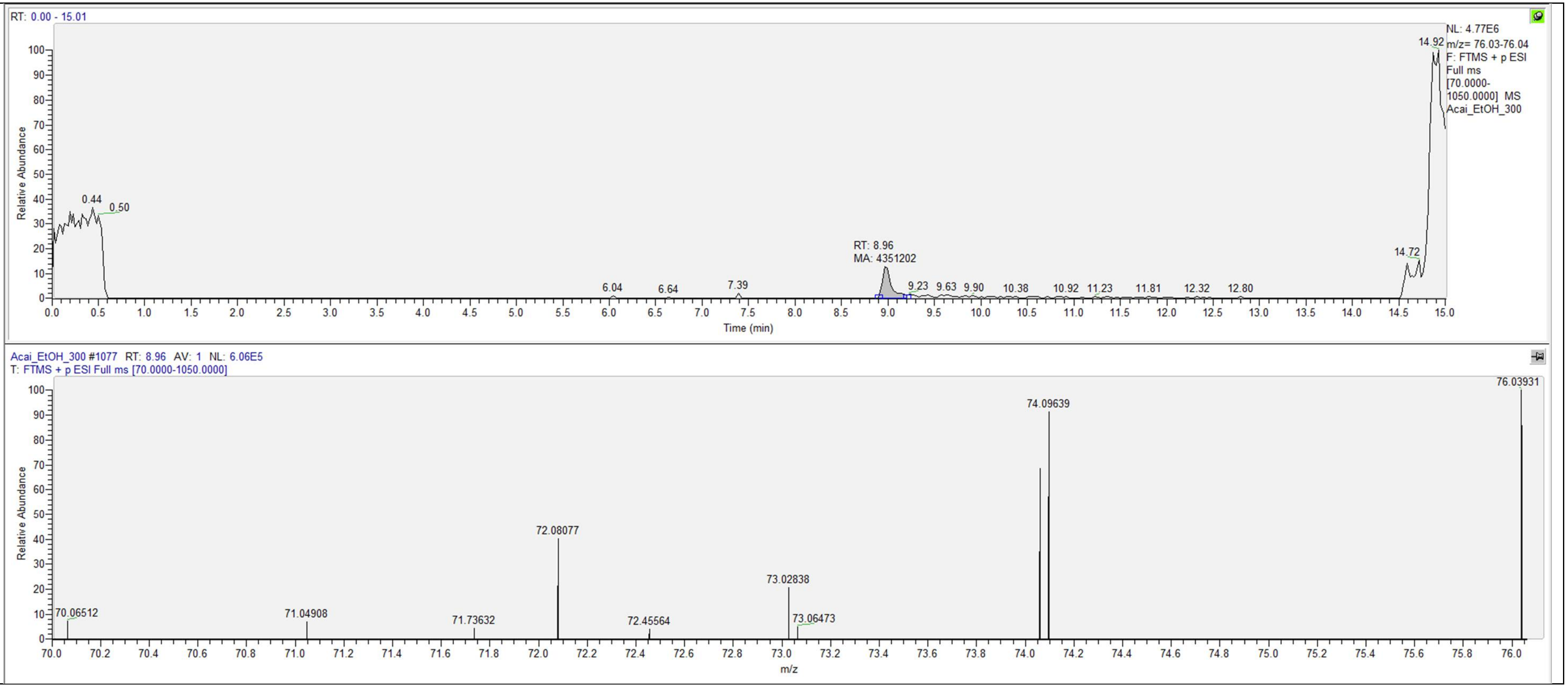RT: 0.00 - 15.01
NL: 4.77E6
m/z= 76.03-76.04
F: FTMS + p ESI
Full ms
[70.0000-
1050.0000] MS
Acai_EtOH_300
Relative Abundance
Time (min)
RT: 8.96
MA: 4351202
Acai_EtOH_300 #1077 RT: 8.96 AV: 1 NL: 6.06E5
T: FTMS + p ESI Full ms [70.0000-1050.0000]
76.03931
74.09639
72.08077
73.02838
70.06512
71.04908
71.73632
72.45564
73.06473
m/z

Threonine	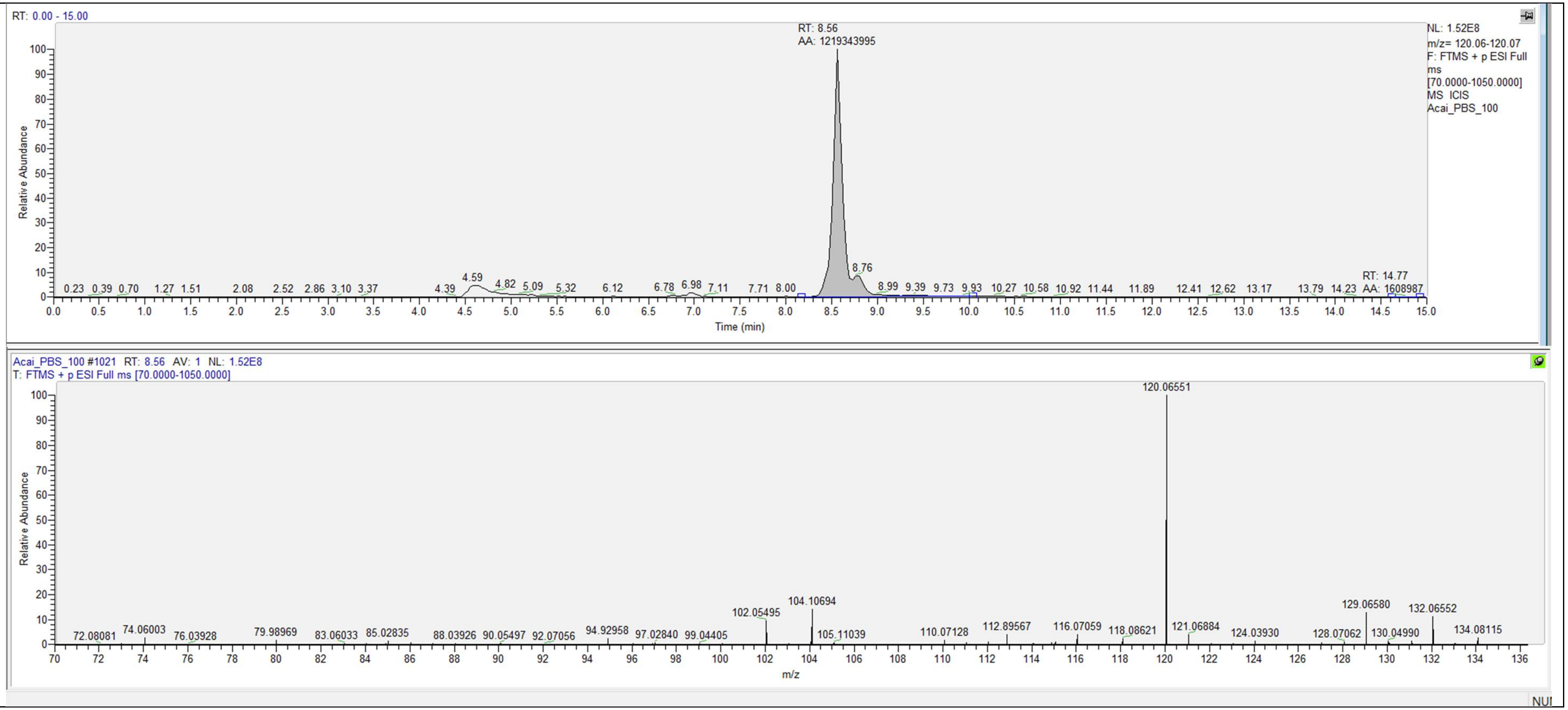

RT: 0.00 - 15.00
RT: 8.56
AA: 1219343995
NL: 1.52E8
m/z= 120.06-120.07
F: FTMS + p ESI Full ms [70.0000-1050.0000]
MS ICIS
Acai_PBS_100
RT: 14.77
AA: 1608987
Relative Abundance
Time (min)
Acai_PBS_100 #1021 RT: 8.56 AV: 1 NL: 1.52E8
T: FTMS + p ESI Full ms [70.0000-1050.0000]
120.06551
104.10694
102.05495
129.06580
132.06552
134.08115
121.06884
116.07059
112.89567
m/z

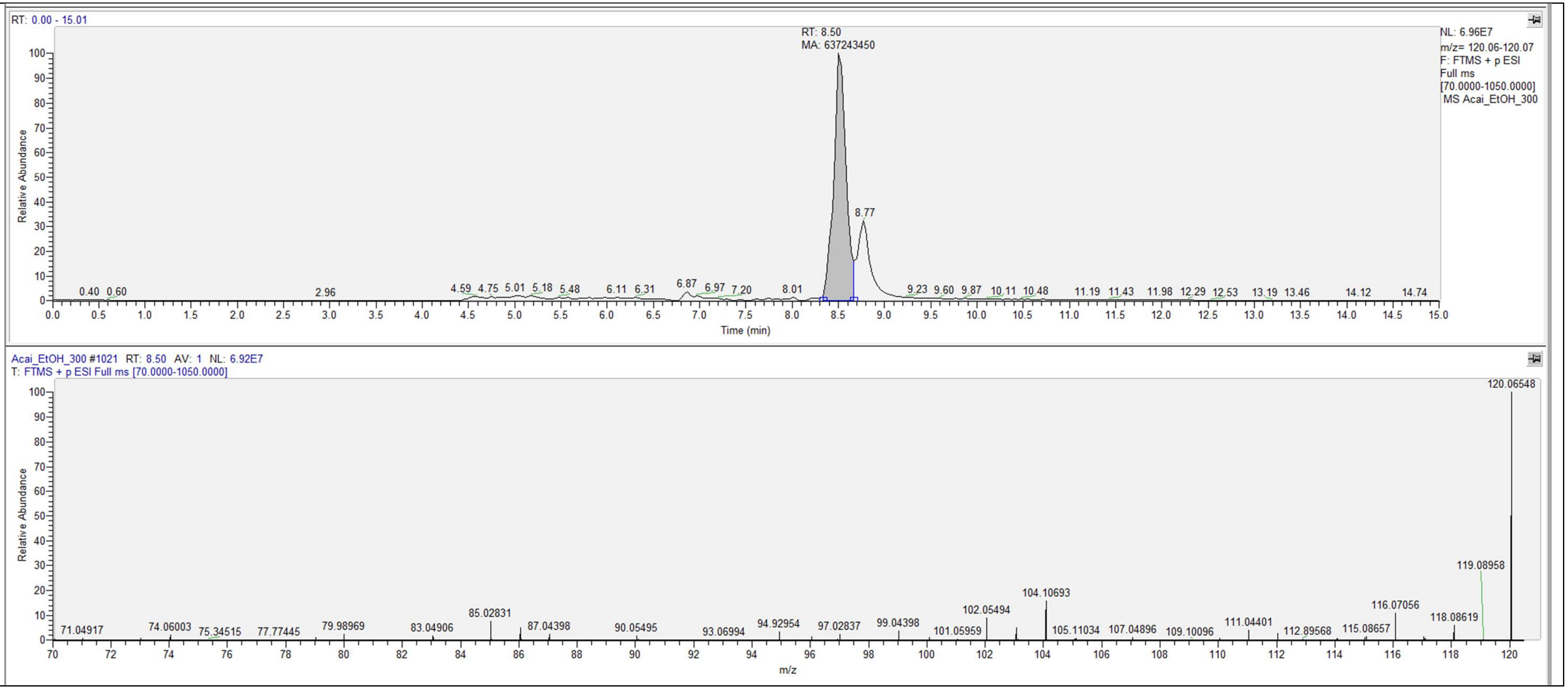
RT: 0.00 - 15.01
RT: 8.50
MA: 637243450
NL: 6.96E7
m/z= 120.06-120.07
F: FTMS + p ESI
Full ms
[70.0000-1050.0000]
MS Acai_EtOH_300
Relative Abundance
Time (min)
Acai_EtOH_300 #1021 RT: 8.50 AV: 1 NL: 6.92E7
T: FTMS + p ESI Full ms [70.0000-1050.0000]
120.06548
119.08958
104.10693
102.05494
116.07056
118.08619
85.02831
m/z

Histidine	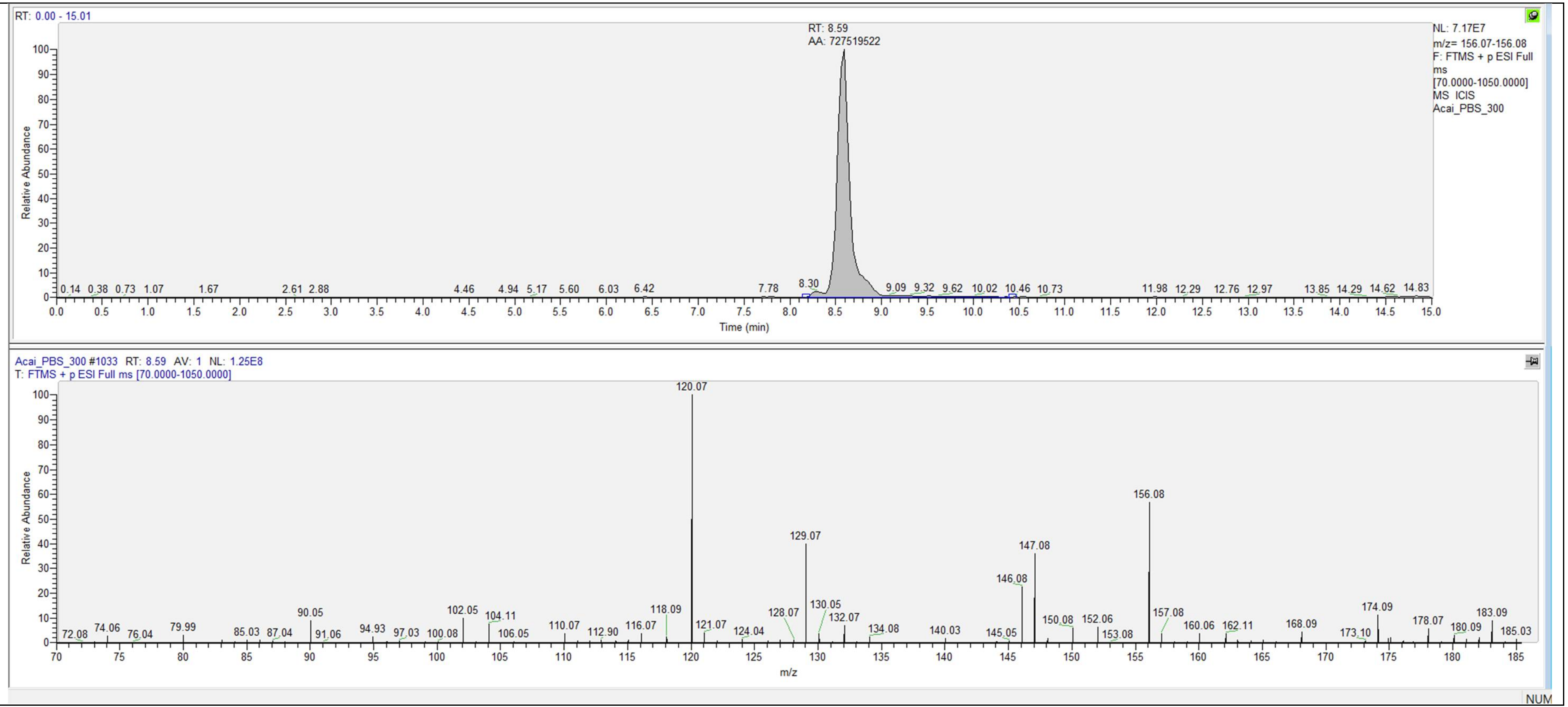

RT: 0.00 - 15.01
RT: 8.59
AA: 727519522
NL: 7.17E7
m/z= 156.07-156.08
F: FTMS + p ESI Full ms [70.0000-1050.0000] MS ICIS Acai_PBS_300
Relative Abundance
Time (min)
Acai_PBS_300 #1033 RT: 8.59 AV: 1 NL: 1.25E8
T: FTMS + p ESI Full ms [70.0000-1050.0000]
120.07
156.08
129.07
147.08
146.08
m/z

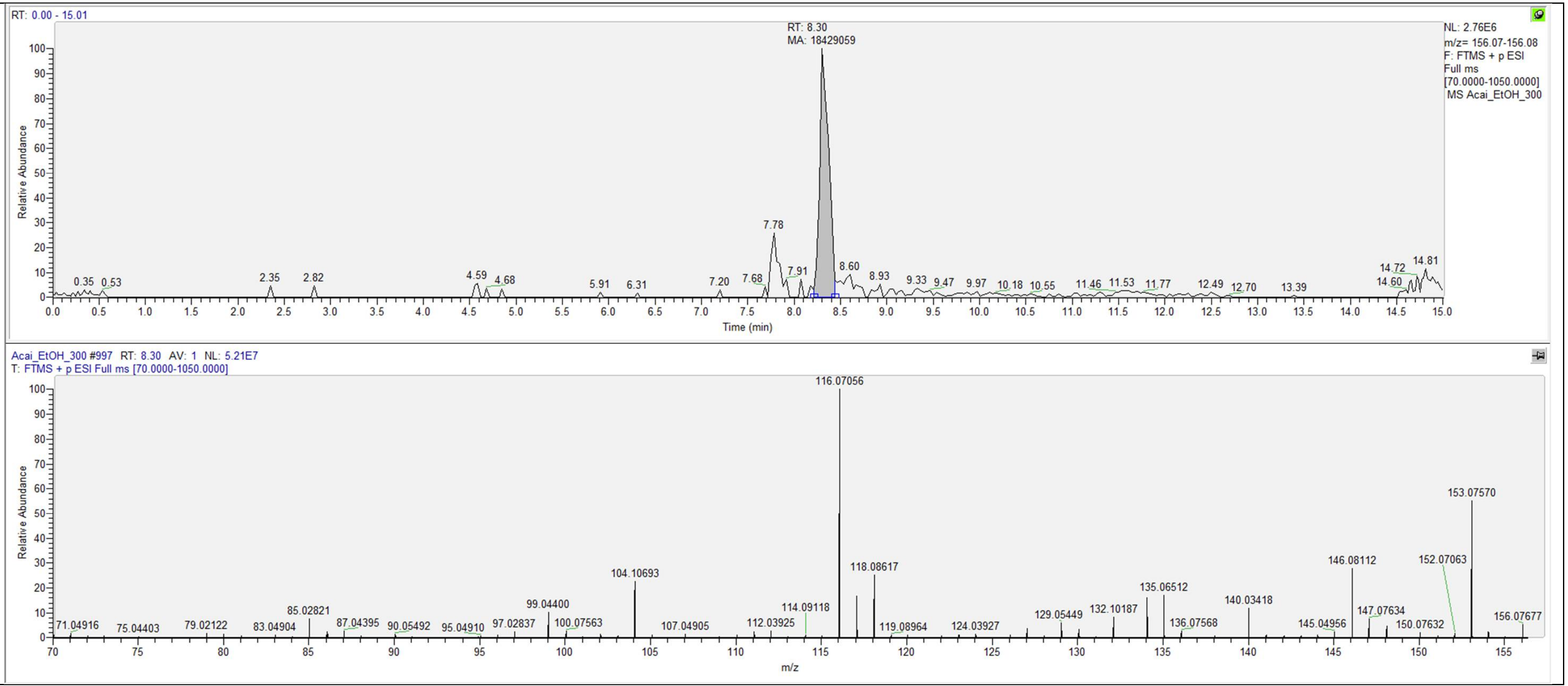

RT: 0.00 - 15.01
RT: 8.30
MA: 18429059
NL: 2.76E6
m/z= 156.07-156.08
F: FTMS + p ESI
Full ms
[70.0000-1050.0000]
MS Acai_EtOH_300
Relative Abundance
Time (min)
Acai_EtOH_300 #997 RT: 8.30 AV: 1 NL: 5.21E7
T: FTMS + p ESI Full ms [70.0000-1050.0000]
116.07056
153.07570
146.08112
152.07063
118.08617
104.10693
135.06512
140.03418
99.04400
85.02821
m/z

Tryptophan	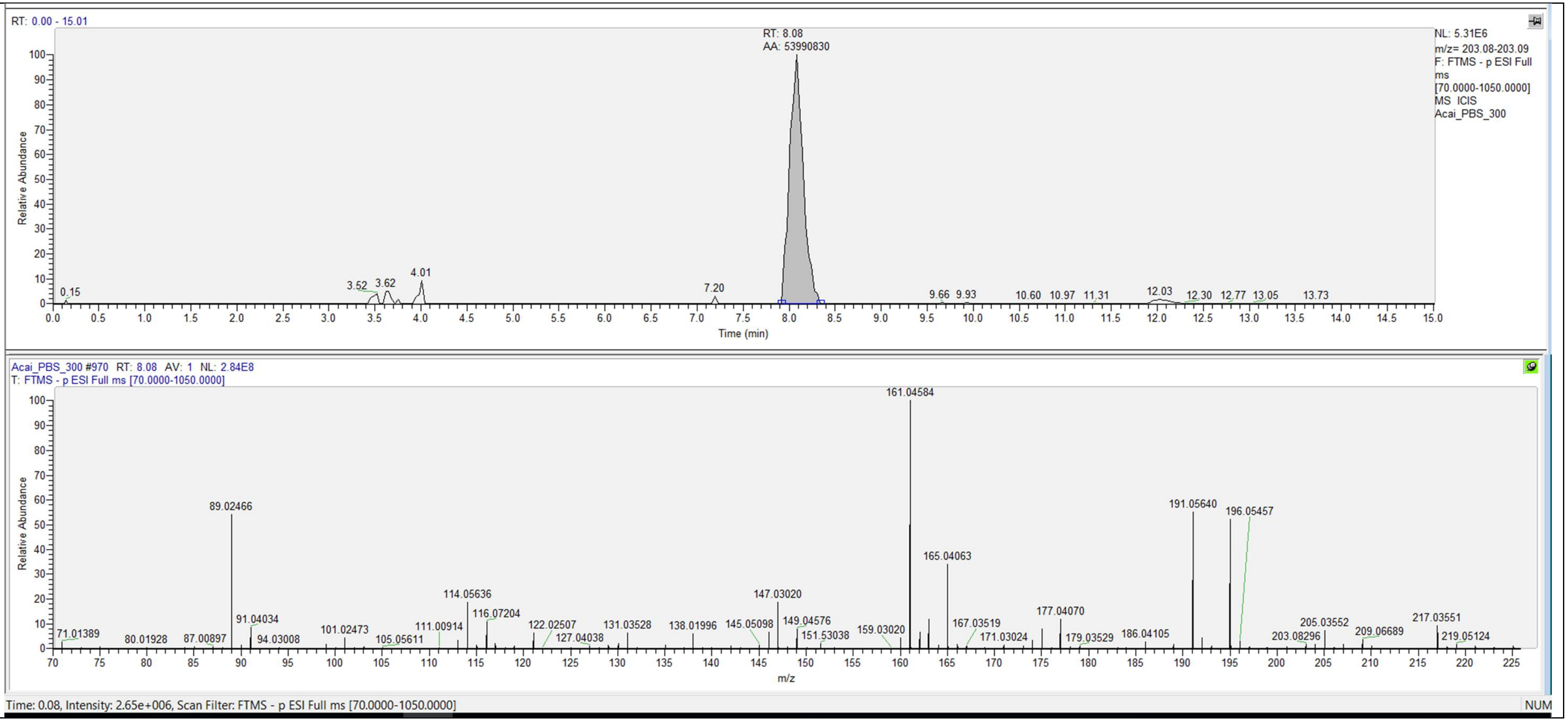

RT: 0.00 - 15.01
RT: 8.08
AA: 53990830
NL: 5.31E6
m/z= 203.08-203.09
F: FTMS - p ESI Full ms [70.0000-1050.0000]
MS ICIS
Acai_PBS_300
Relative Abundance
0.15
3.52
3.62
4.01
7.20
9.66
9.93
10.60
10.97
11.31
12.03
12.30
12.77
13.05
13.73
Time (min)
Acai_PBS_300 #970 RT: 8.08 AV: 1 NL: 2.84E8
T: FTMS - p ESI Full ms [70.0000-1050.0000]
161.04584
89.02466
191.05640
196.05457
165.04063
147.03020
114.05636
116.07204
91.04034
177.04070
217.03551
205.03552
71.01389
80.01928
87.00897
94.03008
101.02473
105.05611
111.00914
122.02507
127.04038
131.03528
138.01996
145.05098
149.04576
151.53038
159.03020
167.03519
171.03024
179.03529
186.04105
203.08296
209.06689
219.05124
m/z
Time: 0.08, Intensity: 2.65e+006, Scan Filter: FTMS - p ESI Full ms [70.0000-1050.0000]
NUM

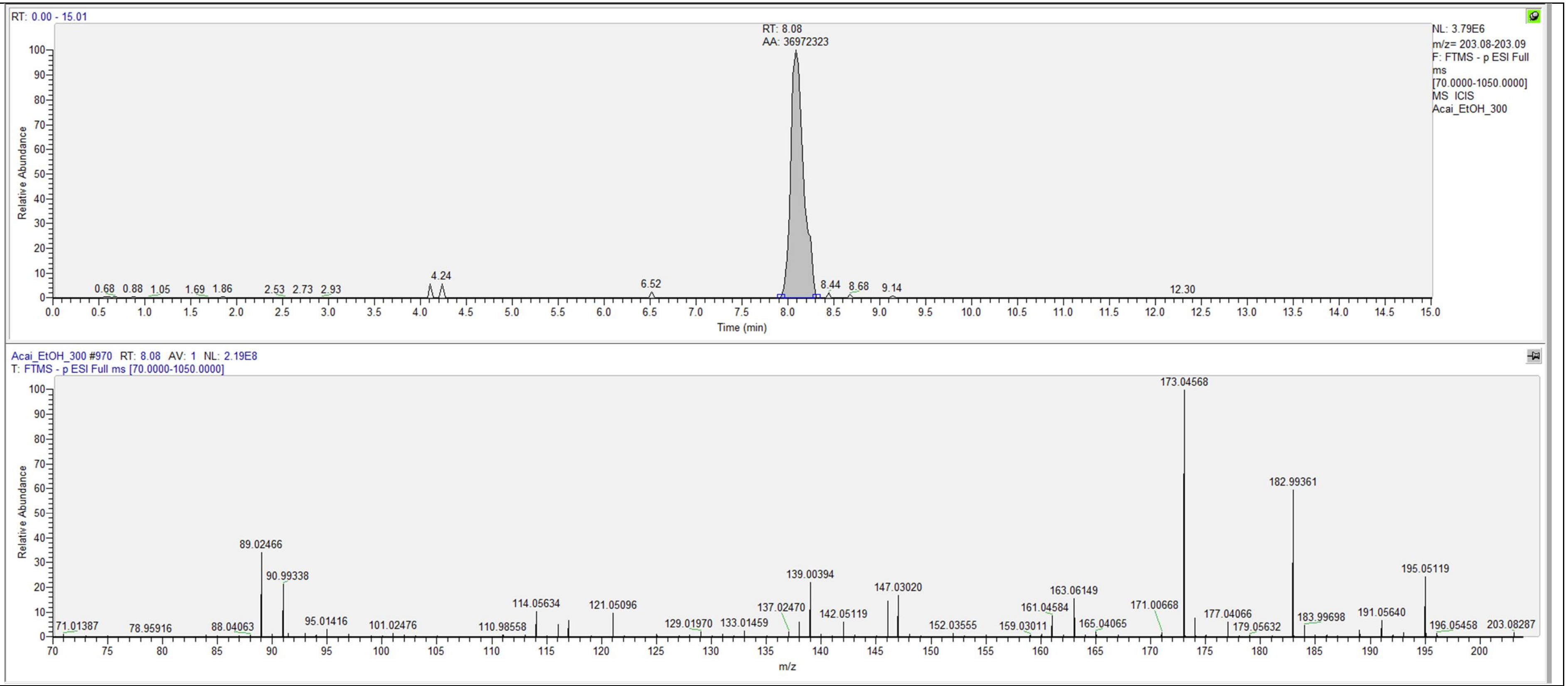
RT: 0.00 - 15.01
RT: 8.08
AA: 36972323
NL: 3.79E6
m/z= 203.08-203.09
F: FTMS - p ESI Full ms [70.0000-1050.0000]
MS ICIS
Acai_EtOH_300
Relative Abundance
0.68 0.88 1.05 1.69 1.86 2.53 2.73 2.93 4.24 6.52 8.44 8.68 9.14 12.30
Time (min)
Acai_EtOH_300 #970 RT: 8.08 AV: 1 NL: 2.19E8
T: FTMS - p ESI Full ms [70.0000-1050.0000]
173.04568
182.99361
89.02466
90.99338
139.00394
195.05119
147.03020
163.06149
114.05634
121.05096
137.02470
161.04584
171.00668
142.05119
177.04066
191.05640
183.99698
95.01416
71.01387
78.95916
88.04063
101.02476
110.98558
129.01970
133.01459
152.03555
159.03011
165.04065
179.05632
196.05458
203.08287
m/z

Tyrosine	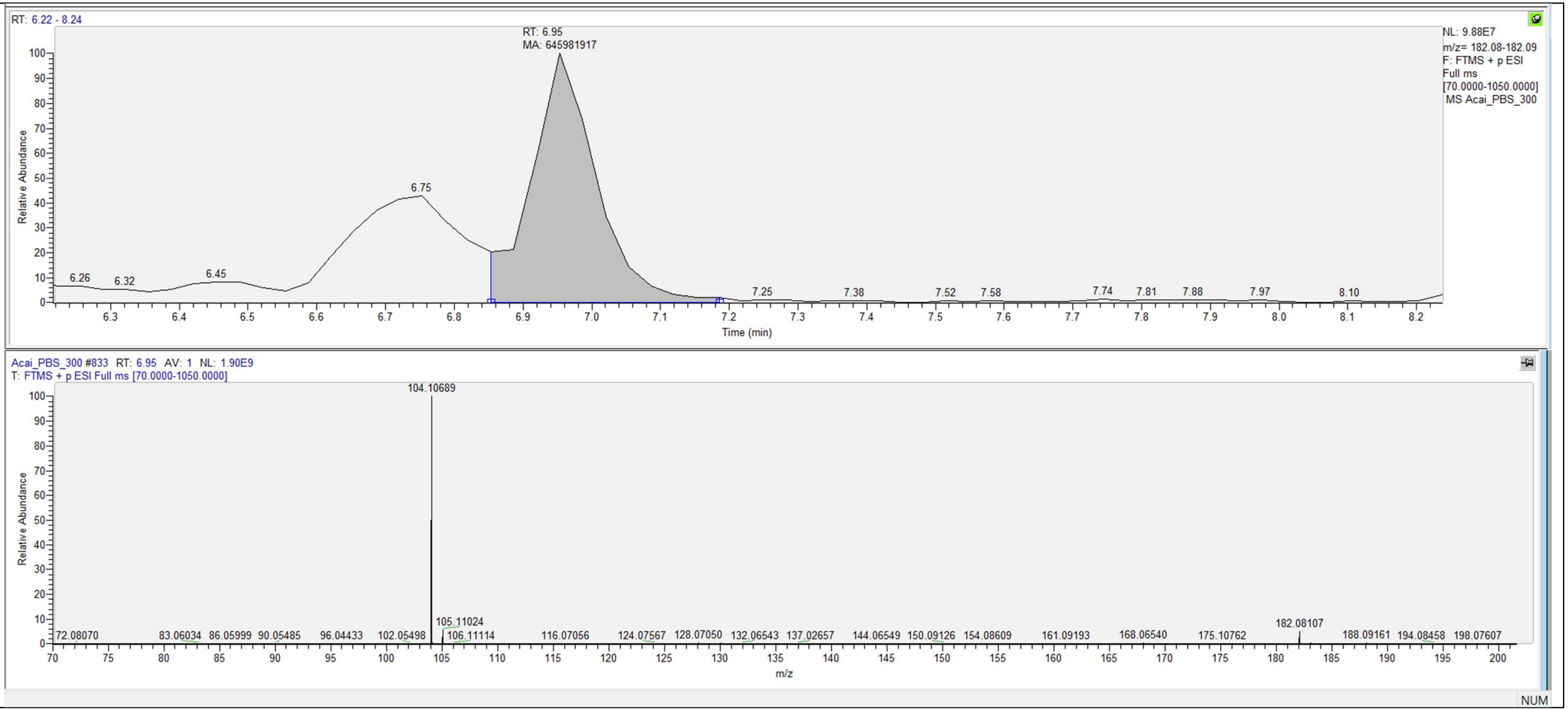

RT: 6.22 - 8.24
RT: 6.95
MA: 645981917
NL: 9.88E7
m/z= 182.08-182.09
F: FTMS + p ESI
Full ms
[70.0000-1050.0000]
MS Acai_PBS_300
6.26 6.32 6.45 6.75 7.25 7.38 7.52 7.58 7.74 7.81 7.88 7.97 8.10
Relative Abundance
Time (min)
Acai_PBS_300 #833 RT: 6.95 AV: 1 NL: 1.90E9
T: FTMS + p ESI Full ms [70.0000-1050.0000]
104.10689
105.11024
106.11114
72.08070 83.06034 86.05999 90.05485 96.04433 102.05498 116.07056 124.07567 128.07050 132.06543 137.02657 144.06549 150.09126 154.08609 161.09193 168.06540 175.10762 182.08107 188.09161 194.08458 198.07607
m/z
NUM

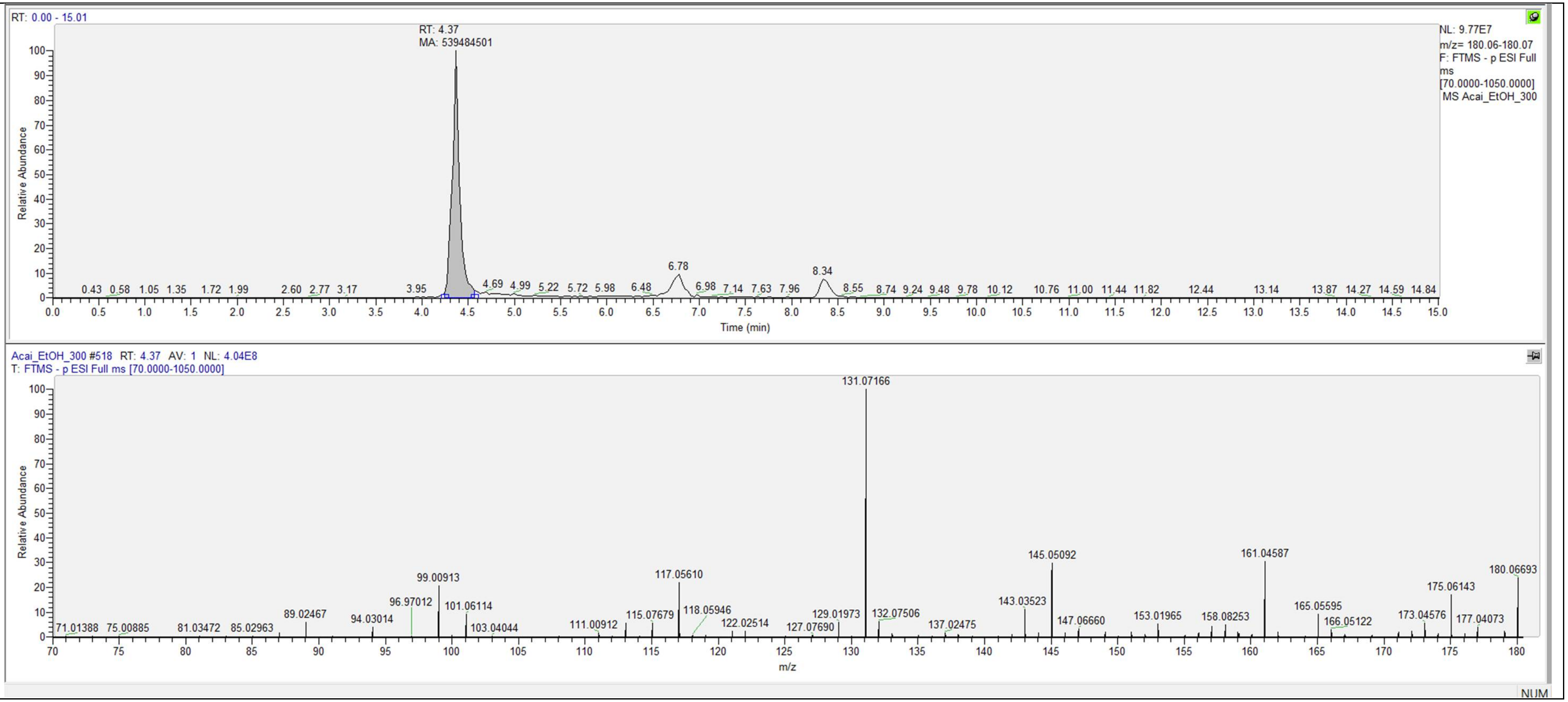
RT: 0.00 - 15.01
RT: 4.37
MA: 539484501
NL: 9.77E7
m/z= 180.06-180.07
F: FTMS - p ESI Full ms [70.0000-1050.0000]
MS Acai_EtOH_300
Relative Abundance
Time (min)
Acai_EtOH_300 #518 RT: 4.37 AV: 1 NL: 4.04E8
T: FTMS - p ESI Full ms [70.0000-1050.0000]
131.07166
145.05092
161.04587
180.06693
175.06143
117.05610
99.00913
m/z

Isoleucine	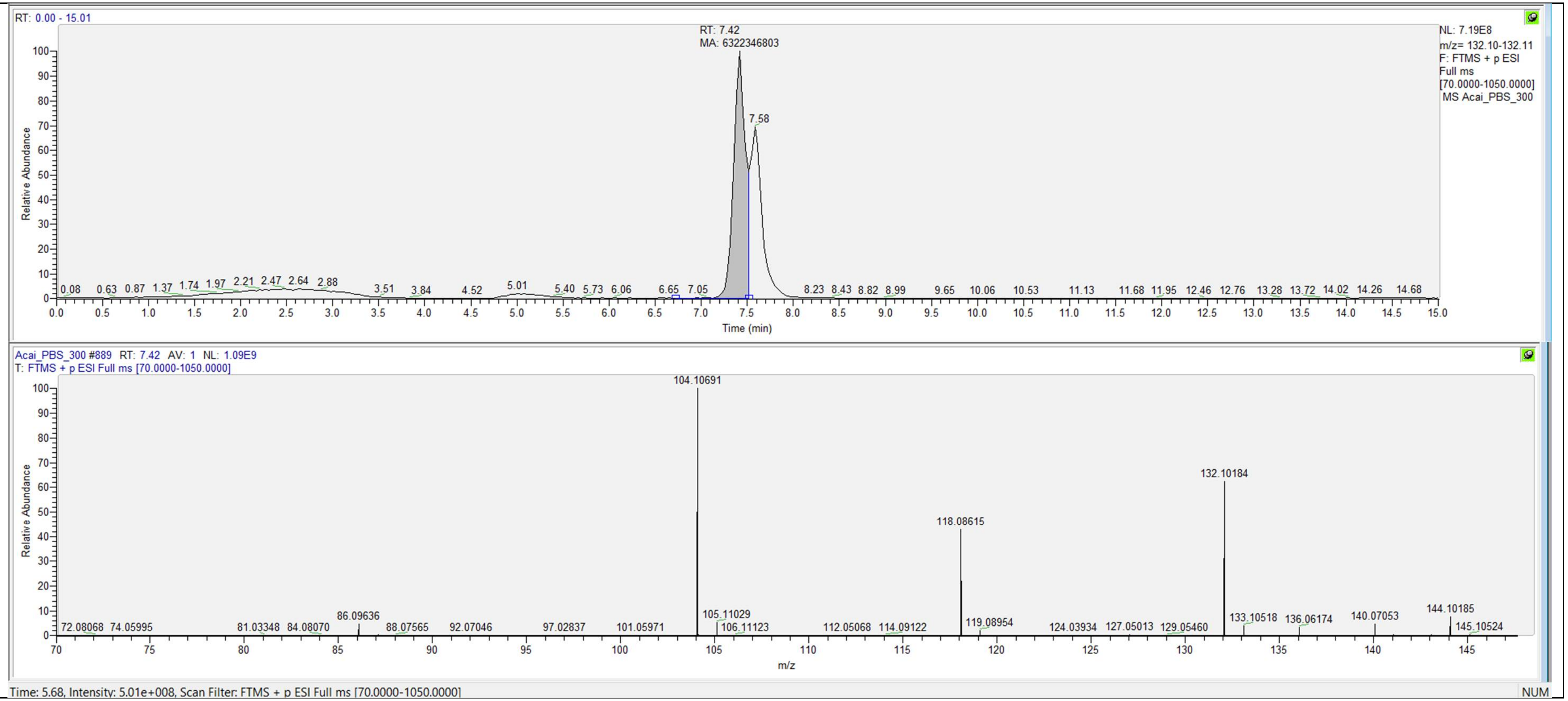

RT: 0.00 - 15.01
RT: 7.42
MA: 6322346803
7.58
NL: 7.19E8
m/z= 132.10-132.11
F: FTMS + p ESI
Full ms
[70.0000-1050.0000]
MS Acai_PBS_300
Relative Abundance
Time (min)
Acai_PBS_300 #889 RT: 7.42 AV: 1 NL: 1.09E9
T: FTMS + p ESI Full ms [70.0000-1050.0000]
104.10691
132.10184
118.08615
144.10185
m/z
Time: 5.68, Intensity: 5.01e+008, Scan Filter: FTMS + p ESI Full ms [70.0000-1050.0000]
NUM

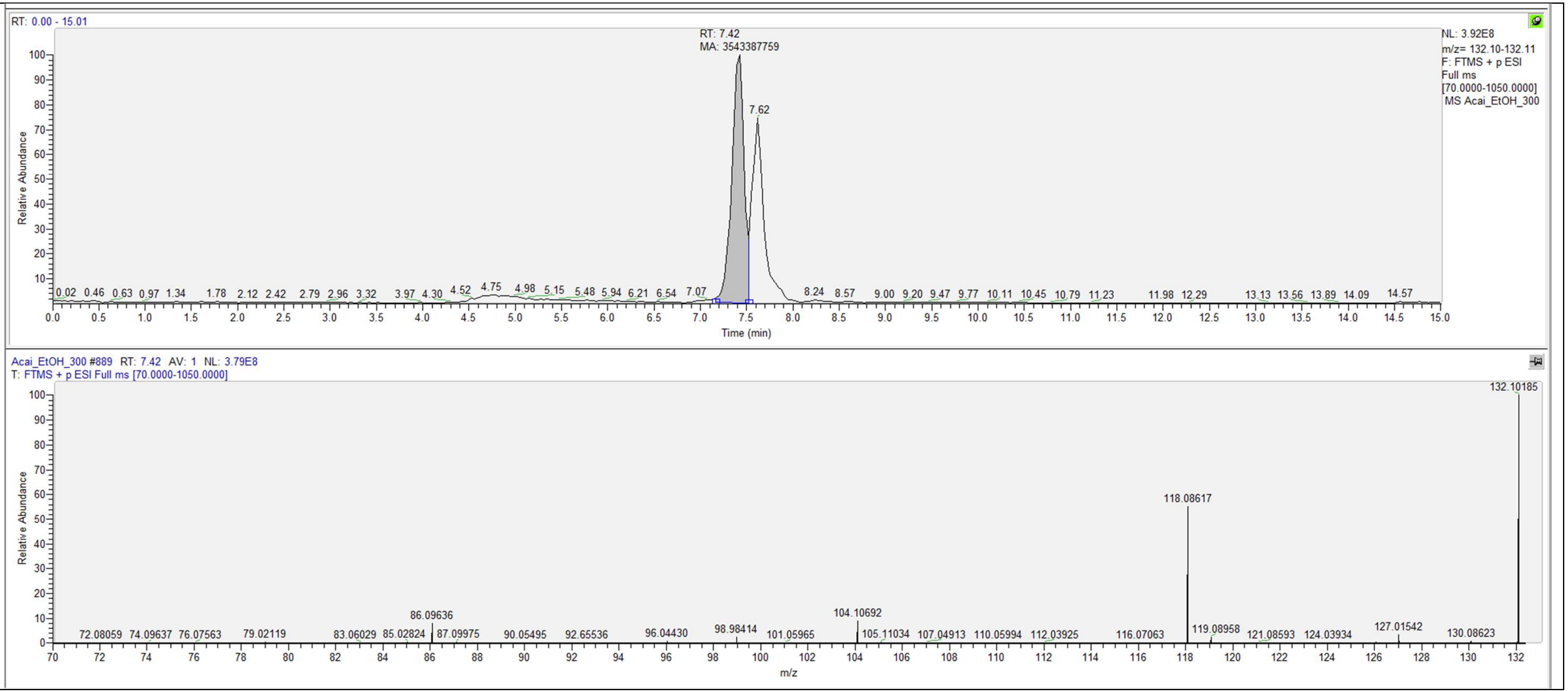

RT: 0.00 - 15.01
RT: 7.42
MA: 3543387759
7.62
NL: 3.92E8
m/z= 132.10-132.11
F: FTMS + p ESI
Full ms
[70.0000-1050.0000]
MS Acai_EtOH_300
Relative Abundance
Time (min)
Acai_EtOH_300 #889 RT: 7.42 AV: 1 NL: 3.79E8
T: FTMS + p ESI Full ms [70.0000-1050.0000]
132.10185
118.08617
104.10692
86.09636
m/z

Valine	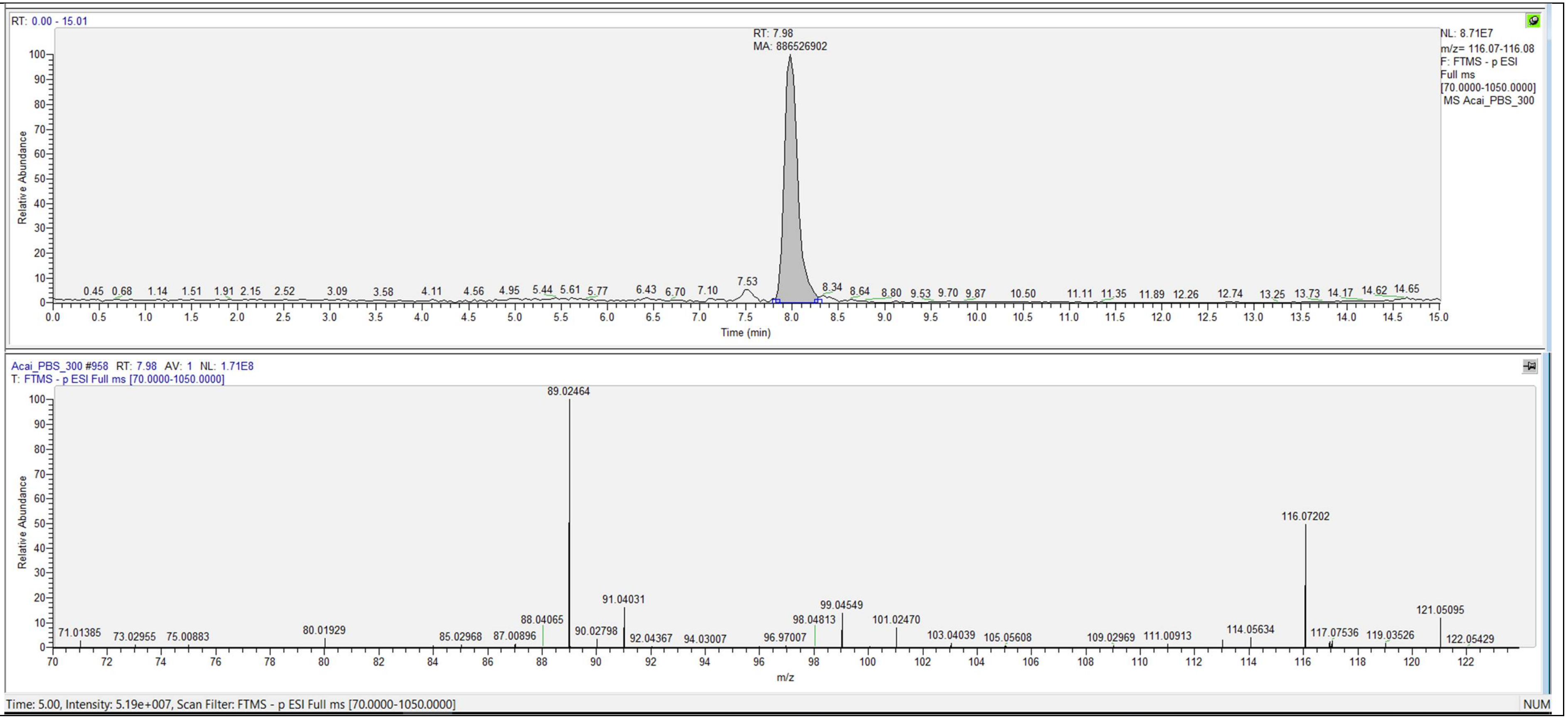

RT: 0.00 - 15.01
RT: 7.98
MA: 886526902
NL: 8.71E7
m/z= 116.07-116.08
F: FTMS - p ESI
Full ms
[70.0000-1050.0000]
MS Acai_PBS_300
Relative Abundance
Time (min)
Acai_PBS_300 #958 RT: 7.98 AV: 1 NL: 1.71E8
T: FTMS - p ESI Full ms [70.0000-1050.0000]
89.02464
116.07202
91.04031
99.04549
98.04813
101.02470
88.04065
121.05095
m/z
Time: 5.00, Intensity: 5.19e+007, Scan Filter: FTMS - p ESI Full ms [70.0000-1050.0000]
NUM

	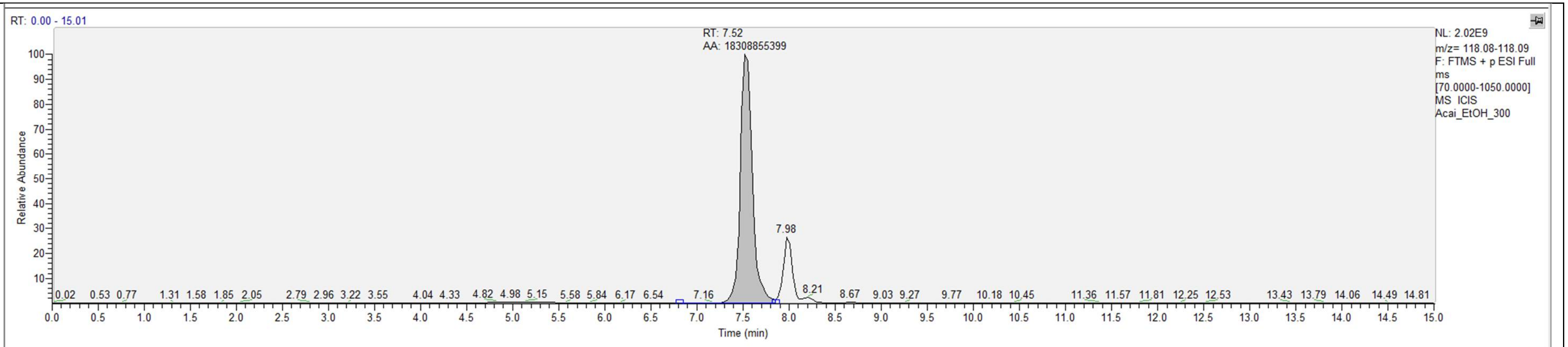RT: 0.00 - 15.01
RT: 7.52
AA: 18308855399
NL: 2.02E9
m/z= 118.08-118.09
F: FTMS + p ESI Full ms [70.0000-1050.0000]
MS ICIS
Acai_EtOH_300
Relative Abundance
Time (min)
7.98
8.21 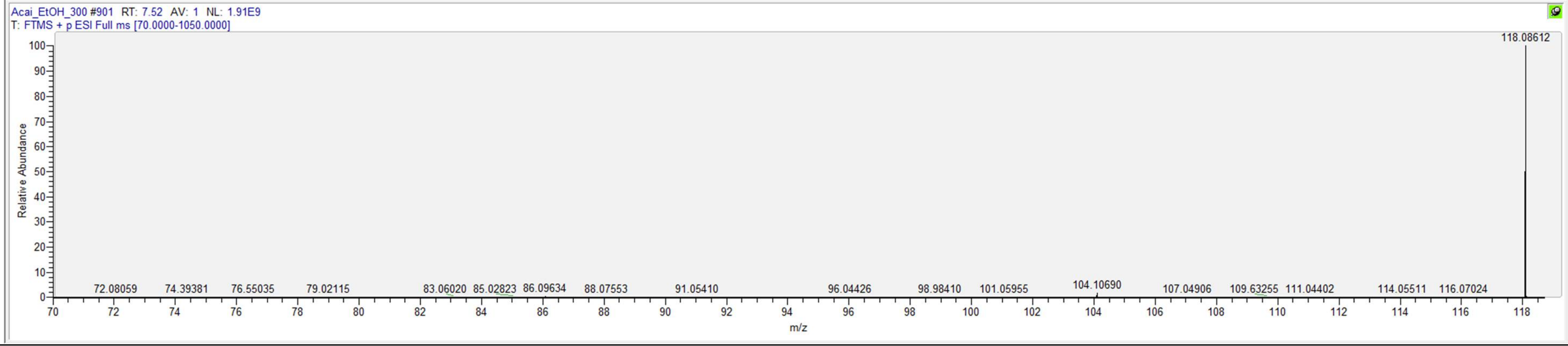Acai_EtOH_300 #901 RT: 7.52 AV: 1 NL: 1.91E9
T: FTMS + p ESI Full ms [70.0000-1050.0000]
118.08612
Relative Abundance
m/z
104.10690

Leucine	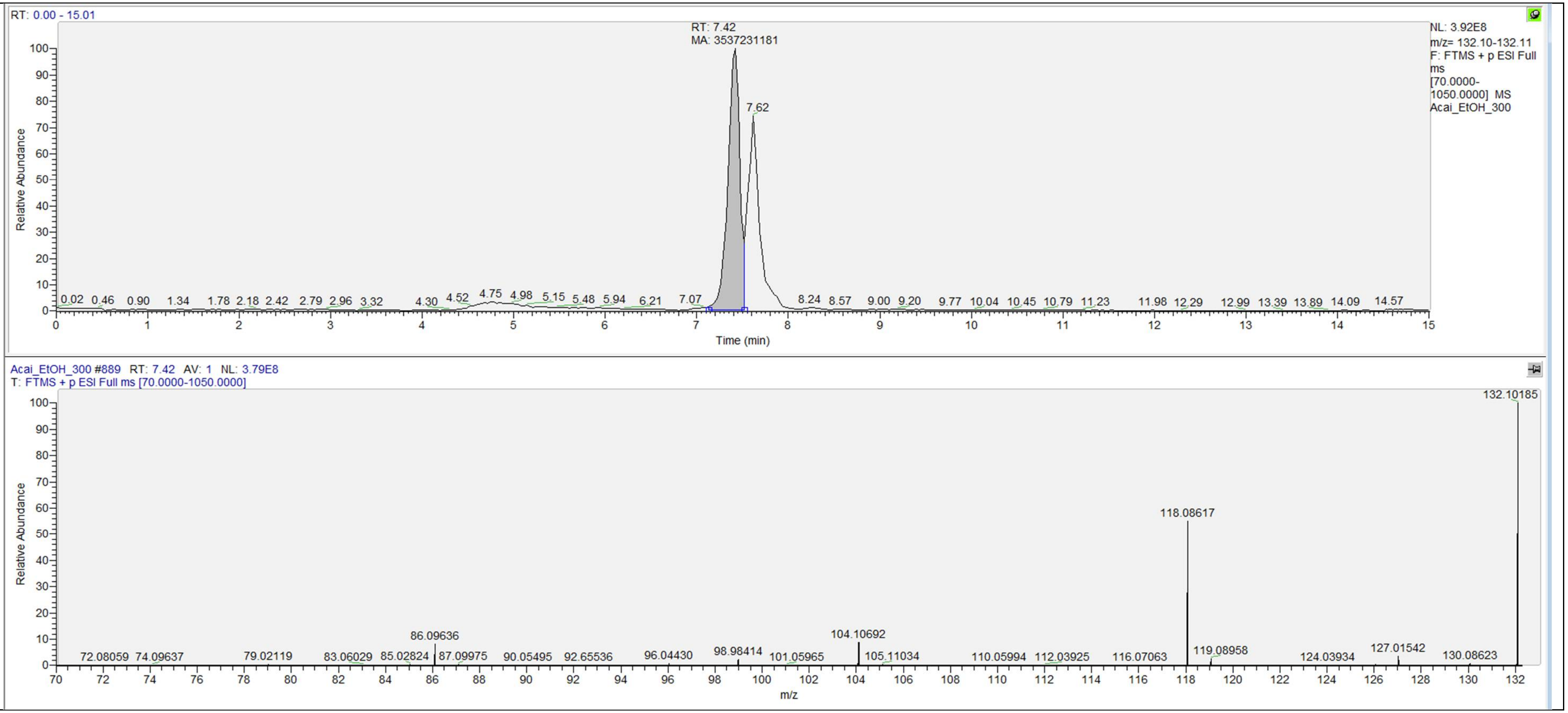

RT: 0.00 - 15.01
RT: 7.42
MA: 3537231181
7.62
NL: 3.92E8
m/z= 132.10-132.11
F: FTMS + p ESI Full ms [70.0000-1050.0000] MS
Acai_EtOH_300
Relative Abundance
Time (min)
Acai_EtOH_300 #889 RT: 7.42 AV: 1 NL: 3.79E8
T: FTMS + p ESI Full ms [70.0000-1050.0000]
132.10185
118.08617
104.10692
86.09636
m/z

Cellotetraose	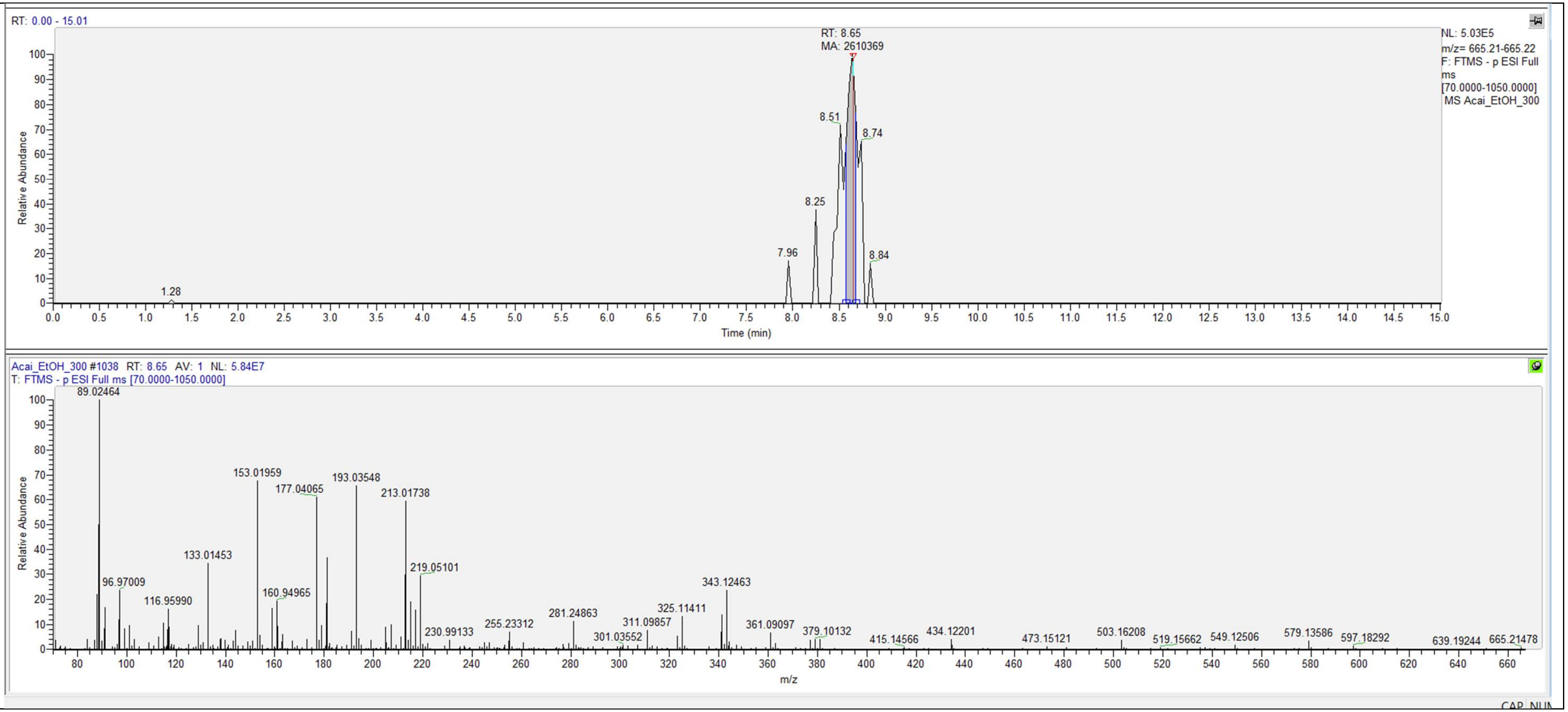

Sucrose

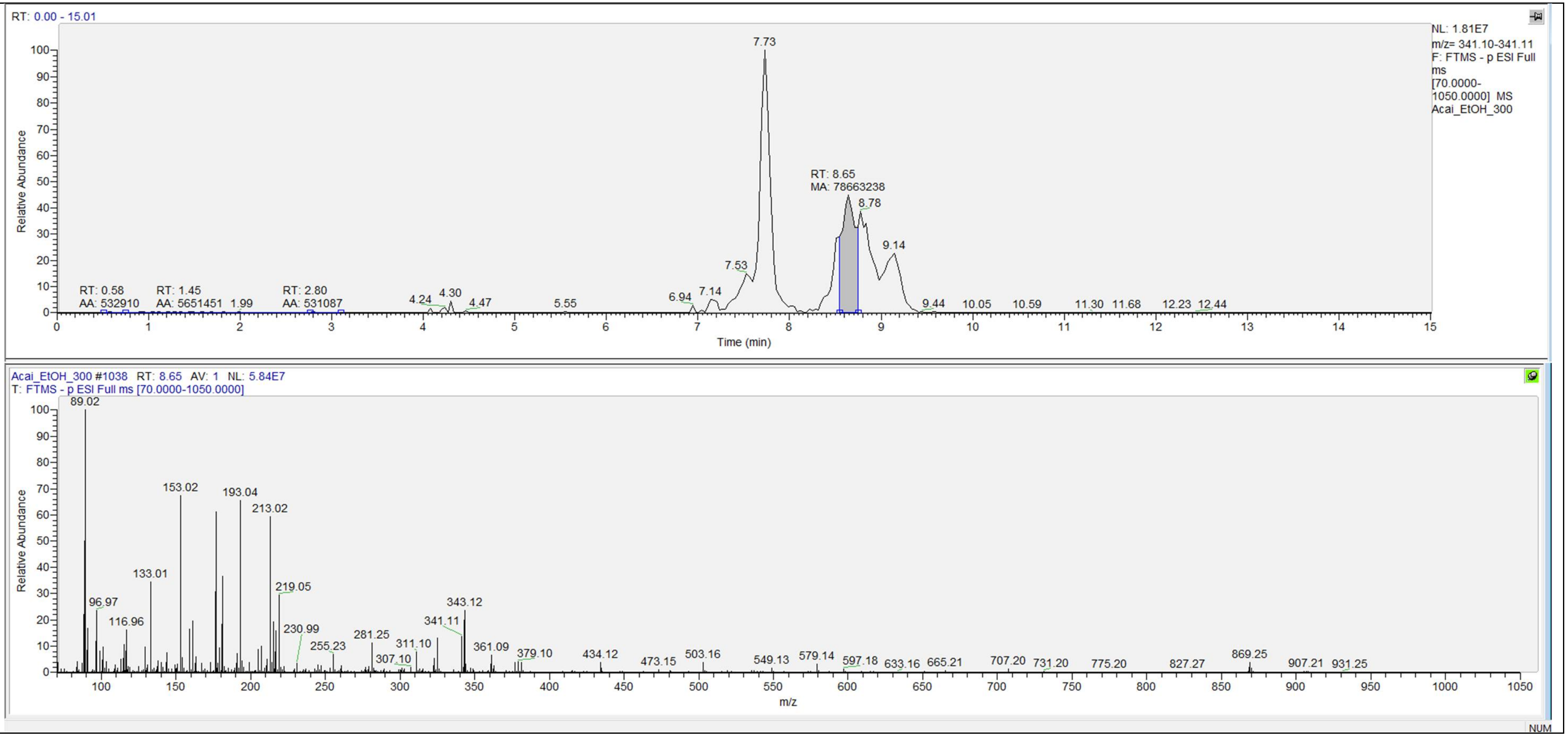
RT: 0.00 - 15.01
NL: 1.81E7
m/z= 341.10-341.11
F: FTMS - p ESI Full ms [70.0000-1050.0000] MS
Acai_EtOH_300
Relative Abundance
Time (min)
7.73
RT: 8.65
MA: 78663238
8.78
9.14
7.53
7.14
6.94
RT: 0.58
AA: 532910
RT: 1.45
AA: 5651451
1.99
RT: 2.80
AA: 531087
4.24
4.30
4.47
5.55
9.44
10.05
10.59
11.30
11.68
12.23
12.44
Acai_EtOH_300 #1038 RT: 8.65 AV: 1 NL: 5.84E7
T: FTMS - p ESI Full ms [70.0000-1050.0000]
Relative Abundance
m/z
89.02
153.02
193.04
213.02
133.01
219.05
96.97
116.96
343.12
341.11
230.99
255.23
281.25
311.10
307.10
361.09
379.10
434.12
473.15
503.16
549.13
579.14
597.18
633.16
665.21
707.20
731.20
775.20
827.27
869.25
907.21
931.25
NUM

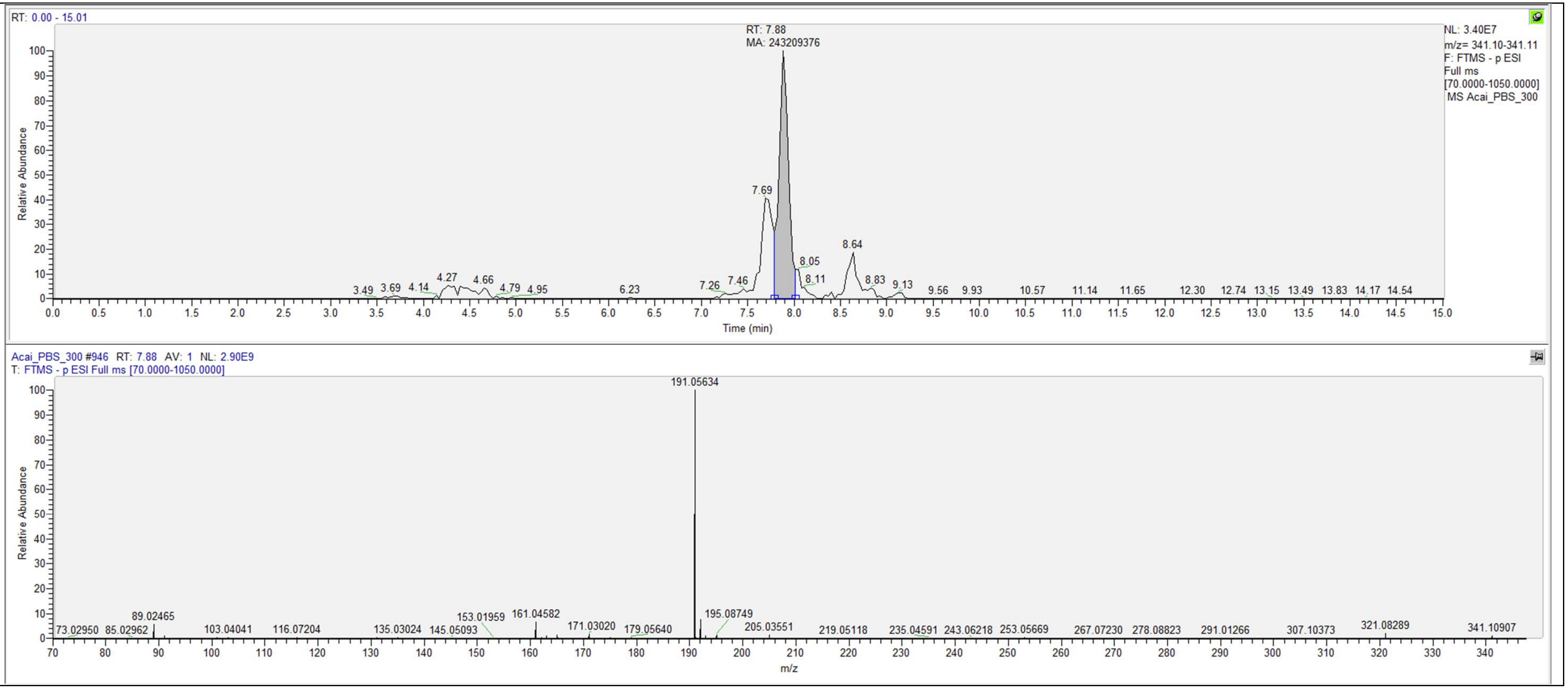

RT: 0.00 - 15.01
RT: 7.88
MA: 243209376
NL: 3.40E7
m/z= 341.10-341.11
F: FTMS - p ESI
Full ms
[70.0000-1050.0000]
MS Acai_PBS_300
Relative Abundance
Time (min)
Acai_PBS_300 #946 RT: 7.88 AV: 1 NL: 2.90E9
T: FTMS - p ESI Full ms [70.0000-1050.0000]
191.05634
Relative Abundance
m/z

Quinic acid isomer 1	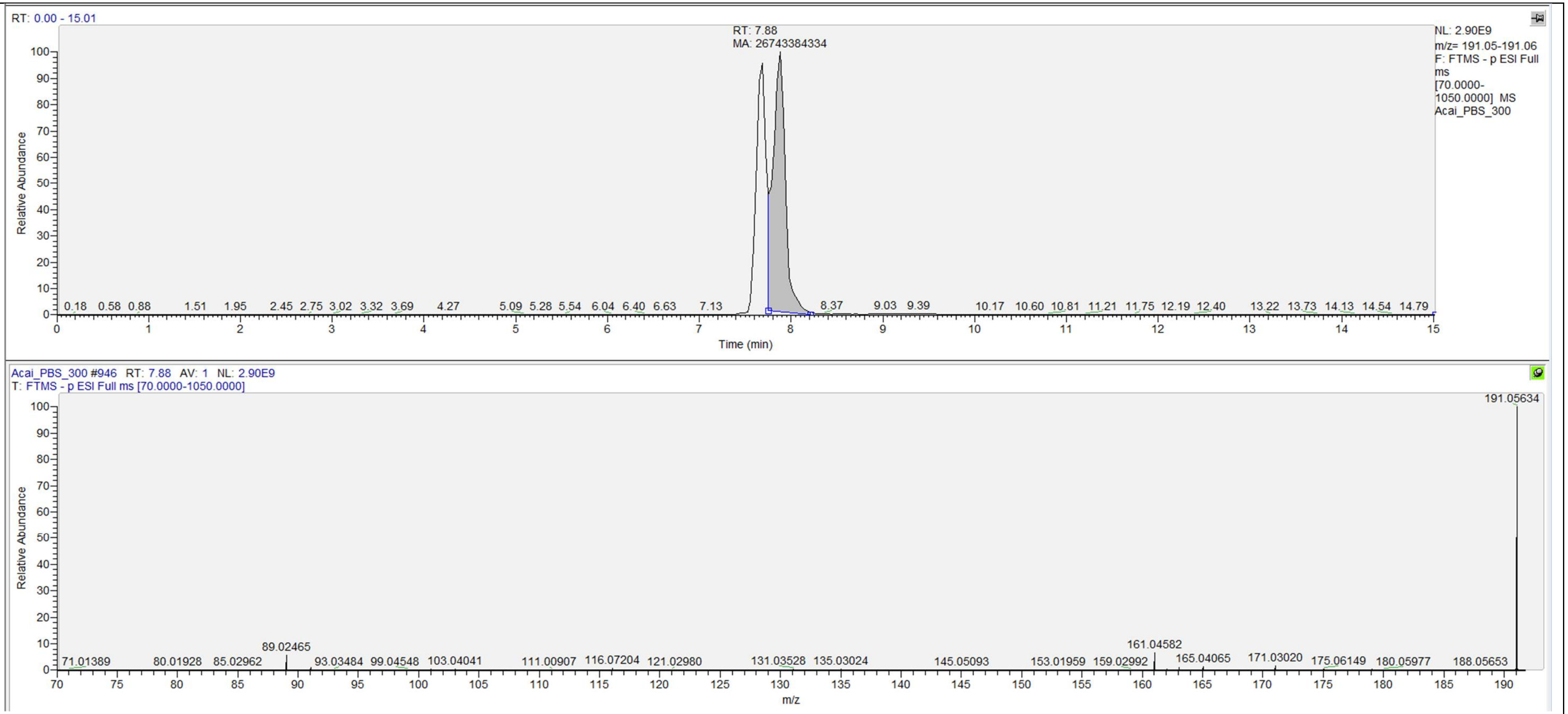

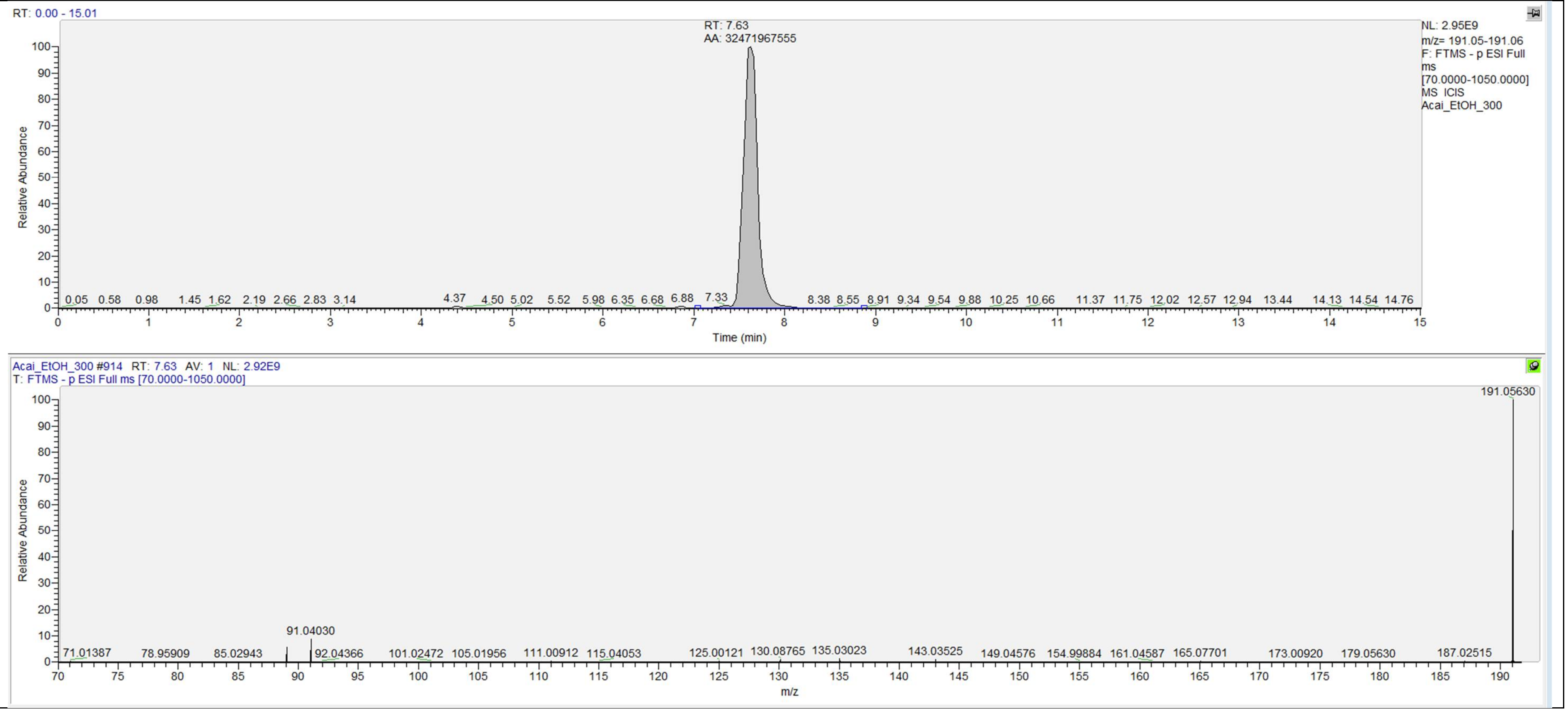
RT: 0.00 - 15.01
RT: 7.63
AA: 32471967555
NL: 2.95E9
m/z= 191.05-191.06
F: FTMS - p ESI Full ms [70.0000-1050.0000]
MS ICIS
Acai_EtOH_300
Relative Abundance
Time (min)
Acai_EtOH_300 #914 RT: 7.63 AV: 1 NL: 2.92E9
T: FTMS - p ESI Full ms [70.0000-1050.0000]
191.05630
91.04030
Relative Abundance
m/z

Ascorbic acid (Vitamin C)

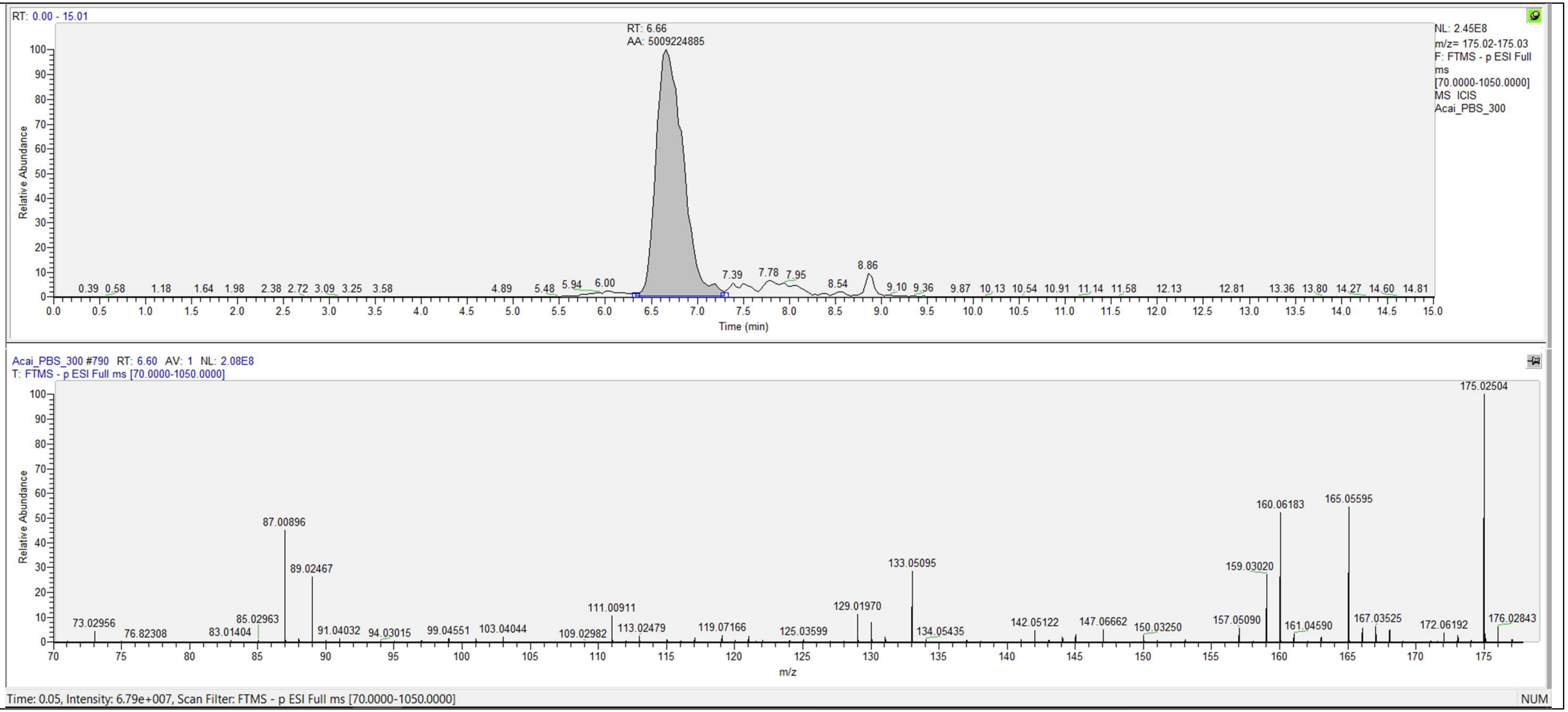

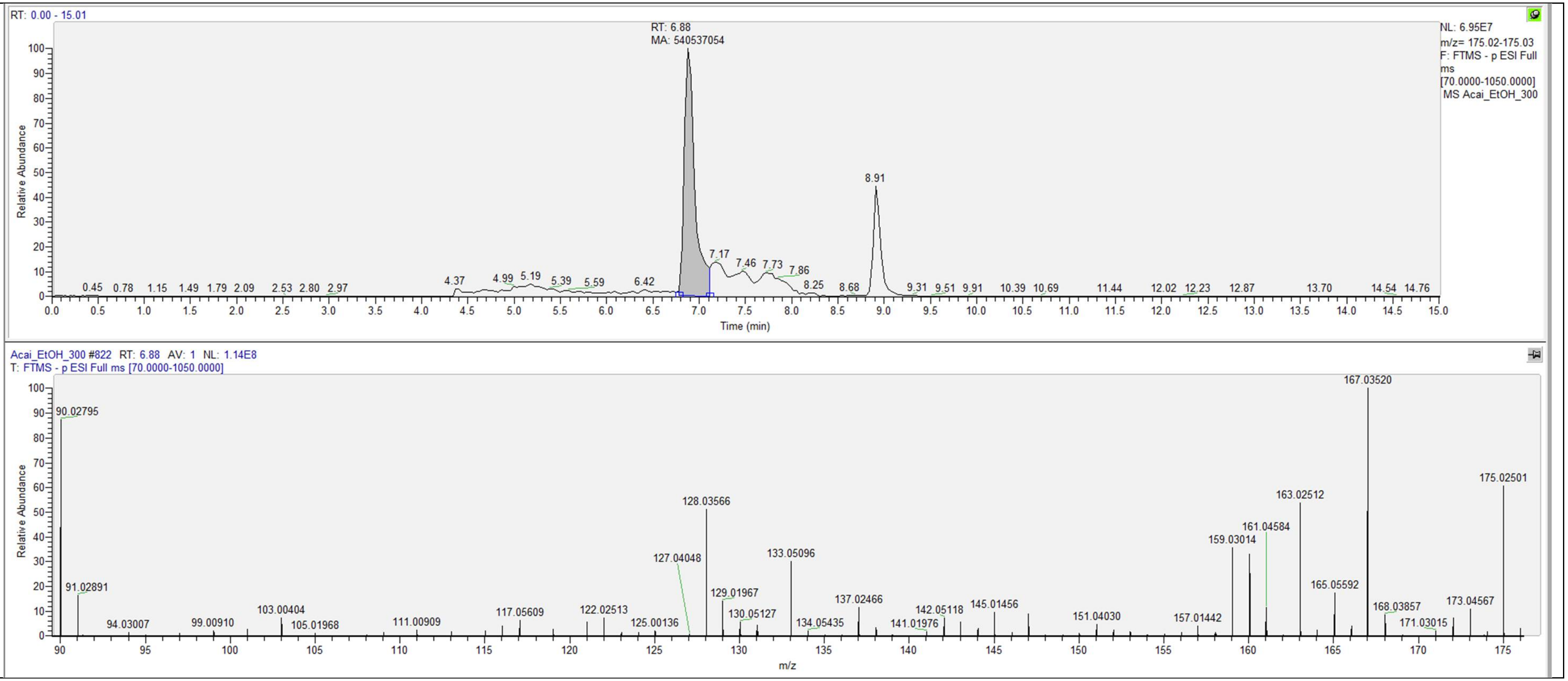
RT: 0.00 - 15.01
RT: 6.88
MA: 540537054
NL: 6.95E7
m/z= 175.02-175.03
F: FTMS - p ESI Full ms [70.0000-1050.0000]
MS Acai_EtOH_300
Relative Abundance
Time (min)
Acai_EtOH_300 #822 RT: 6.88 AV: 1 NL: 1.14E8
T: FTMS - p ESI Full ms [70.0000-1050.0000]
167.03520
175.02501
163.02512
161.04584
159.03014
128.03566
133.05096
127.04048
90.02795
91.02891
m/z
